# Reversing Temozolomide Resistance in Glioblastoma Based on Cuproptosis‐Mediated Positive Feedback Loop

**DOI:** 10.1002/advs.76677

**Published:** 2026-07-20

**Authors:** Wenjie Feng, Rui Yang, Chang Weng, Chen Li, Wanrui Shi, Yang Chen, Yang Yang, Yi Liu, Pengfei Ge, Hao Zhang

**Affiliations:** ^1^ Institute of Translational Medicine The First Hospital of Jilin University Changchun P. R. China; ^2^ Department of Neurosurgery The First Hospital of Jilin University Changchun P. R. China; ^3^ State Key Laboratory of Supramolecular Structure and Materials College of Chemistry Jilin University Changchun P. R. China

**Keywords:** cuproptosis, glioblastoma, positive feedback loop, prodrug, temozolomide resistance

## Abstract

Glioblastoma (GBM), one of the most lethal human cancers, faces a major therapeutic challenge due to drug resistance. Temozolomide (TMZ), a standard first‐line drug for clinical GBM treatment, benefits only a subset of patients, leaving an urgent need for strategies that can restore its efficacy. Herein, we propose that cuproptosis, a recently identified regulated cell death (RCD), is capable of reversing TMZ resistance in GBM. We design copper‐TMZ (Cu‐TMZ) prodrugs, assembled via the coordination of Cu^2+^ with TMZ and, in optimized formulations, co‐loaded with metformin (MET) as an AMPK activator, to exploit cuproptosis for overcoming TMZ resistance. Upon glutathione‐triggered disassembly, the prodrugs release Cu^+^ and TMZ, inducing mitochondrial proteotoxic stress and initiating a self‐amplifying cuproptosis loop characterized by ATP depletion, AMPK‐p53 activation, and glycolysis inhibition. Beyond self‐reinforcement, cuproptosis directly potentiates TMZ activity by suppressing drug efflux, impairing DNA repair, and aggravating metabolic and redox stress. Both in vitro and in vivo experiments demonstrate that Cu‐TMZ/MET prodrugs markedly restore TMZ responsiveness and suppress TMZ‐resistant GBM progression. More broadly, this study illustrates how RCD pathways can be engineered into therapeutics to convert drug resistance into vulnerability, highlighting a generalizable strategy for improving the efficacy of frontline chemotherapy in otherwise intractable cancers.

## Graphical abstract

To effectively reverse TMZ resistance, this work proposes a cuproptosis‐mediated positive feedback loop that integrates mitochondrial dysfunction, ATP depletion, AMPK‐p53 signaling, and glycolytic inhibition, ultimately amplifying cell death. This cuproptosis‐mediated feedback loop simultaneously disrupts multiple resistance pathways in GBM, including redox homeostasis, drug efflux, DNA repair, and energy metabolism, through a coherent signaling cascade.

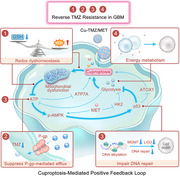

## 1 Introduction

Glioblastoma (GBM), the most common and aggressive primary brain tumor, is characterized by rapid infiltration, frequent recurrence, and extremely poor clinical outcomes, with median survival rarely exceeding 15 months despite maximal multimodal therapy [1, 2]. The current standard of care involves maximally safe surgical resection followed by radiotherapy and chemotherapy, yet complete tumor eradication is virtually impossible due to the diffuse and infiltrative growth pattern of GBM [3, 4]. Temozolomide (TMZ), the standard first‐line chemotherapeutic agent, exerts its cytotoxic effect primarily through DNA alkylation and damage, thereby improving survival in patients with residual tumor burden following surgery [5]. However, nearly half of GBM patients eventually develop resistance to TMZ, severely compromising its therapeutic efficacy. The mechanisms underlying TMZ resistance are complex and multifactorial, involving a network of interrelated cellular pathways. A major contributor is the overexpression of DNA repair systems, particularly O^6^‐methylguanine‐DNA methyltransferase (MGMT), which directly reverses TMZ‐induced alkylation of guanine residues [6]. In addition, approximately 70% of adducts occur at the N7 position of guanine (m^7^G) and 9% at the N3 position of adenine (m^3^A), both of which are primarily repaired by the base excision repair (BER) pathway [7]. Furthermore, glutathione (GSH) level in GBM tissue is approximately 26‑fold higher than that in normal brain tissue, forming a distinct tumor‑specific redox microenvironment [8]. Notably, enhanced antioxidant capacity driven by elevated GSH buffers TMZ‐induced oxidative stress, facilitating resistance [9, 10, 11]. Metabolic reprogramming, particularly the Warburg effect‐mediated reliance on aerobic glycolysis, further sustains GBM proliferation and promotes resistance [12, 13]. Another critical factor is the upregulation of ATP‐dependent efflux transporters such as P‐glycoprotein (P‐gp), which actively extrude TMZ from tumor cells, reducing intracellular drug concentrations and limiting cytotoxicity [14, 15]. Existing therapeutic strategies to overcome TMZ resistance have focused on inhibiting DNA repair systems, modulating redox balance, blocking efflux pumps, or suppressing glycolysis [10, 12, 16, 17, 18, 19, 20]. However, because resistance typically arises from the interplay of multiple pathways, single‐target approaches have attained only limited success. Thus, innovative therapeutic strategies capable of simultaneously disrupting multiple resistance mechanisms are urgently needed to improve clinical outcomes in TMZ‐resistant GBM.

Cuproptosis, a recently identified form of regulated cell death (RCD), provides a promising avenue for overcoming drug resistance in GBM [21, 22]. Unlike apoptosis, necroptosis, or ferroptosis, cuproptosis is triggered by intracellular copper overload [23, 24]. Excess copper binds to lipoylated proteins in the tricarboxylic acid (TCA) cycle, leading to protein aggregation such as dihydrolipoamide S‐acetyltransferase (DLAT), loss of iron‐sulfur (Fe‐S) cluster proteins, proteotoxic stress, and collapse of mitochondrial function [25, 26]. Because of this, cuproptosis is tightly coupled to mitochondrial metabolism. Mitochondrial dysfunction lowers ATP production, elevates the AMP/ATP ratio, and activates AMP‐activated protein kinase (AMPK), a central energy sensor [27]. AMPK, in turn, enhances the transcriptional activity of p53, which suppresses glycolysis and promotes a metabolic shift toward oxidative phosphorylation [28, 29]. Reduced glycolysis limits ATP and pyruvate supply, further sensitizing tumor cells to mitochondrial stress [30, 31, 32, 33, 34]. These interconnected events form a self‐amplifying positive feedback loop. Specifically, mitochondrial dysfunction causes ATP depletion, ATP depletion activates AMPK, AMPK upregulates p53, p53 inhibits glycolysis, and glycolysis inhibition further aggravates ATP shortage, ultimately reinforcing cuproptosis. Thus, cuproptosis not only executes cell death but also intensifies itself through tightly linked metabolic and redox feedback pathways.

Importantly, each signaling node within the cuproptosis‐mediated feedback loop also intersects with mechanisms of TMZ resistance. First, mitochondrial dysfunction and oxidative stress induced by cuproptosis sensitize TMZ‐resistant GBM cells to chemotherapy, while redox dyshomeostasis directly suppresses DNA repair enzymes such as MGMT [10, 16, 35, 36, 37]. Moreover, ATP depletion reduces the activity of P‐gp, thereby limiting drug efflux and enhancing intracellular accumulation of TMZ [38, 39]. Meanwhile, ATP depletion reduces the expression of ATP‐dependent DNA ligase III (LIG3), a key enzyme responsible for the ligation step in the BER pathway [40]. Inhibition of LIG3 leads to incomplete repair of TMZ‐induced m^7^G and m^3^A lesions, thereby enhancing TMZ‐triggered DNA damage and cytotoxicity. Furthermore, AMPK‐mediated p53 activation downregulates MGMT expression, impairing repair of TMZ‐induced DNA lesions [41, 42, 43]. In addition, p53 negatively regulates the copper chaperone antioxidant 1 (ATOX1), which delivers copper to ATPase copper transporting alpha (ATP7A), a copper‐efflux ATPase, that translocates from the Golgi to the plasma membrane to pump copper out of the cell [22, 44, 45, 46, 47]. Reduced ATOX1 expression, together with ATP depletion‐driven suppression of ATP7A activity, diminishes copper efflux and promotes cuprous ion (Cu^+^) retention, thereby sustaining intracellular copper overload. This process exacerbates cuproptosis and further amplifies TMZ sensitivity. Finally, glycolysis inhibition disrupts energy metabolism, suppresses cell proliferation, and reduces lactate production [12, 13, 19, 48]. Since lactate serves as a substrate for lactyltransferases that modify DNA repair enzymes, glycolytic blockade indirectly compromises DNA repair capacity, thereby amplifying TMZ‐induced cytotoxicity [49]. Collectively, these processes establish a therapeutic opportunity in which cuproptosis simultaneously reinforces its own execution and enhances TMZ efficacy, progressively reversing chemoresistance through the convergence of mitochondrial dysfunction, redox imbalance, ATP depletion, p53 activation, and glycolysis inhibition.

**SCHEME 1:**
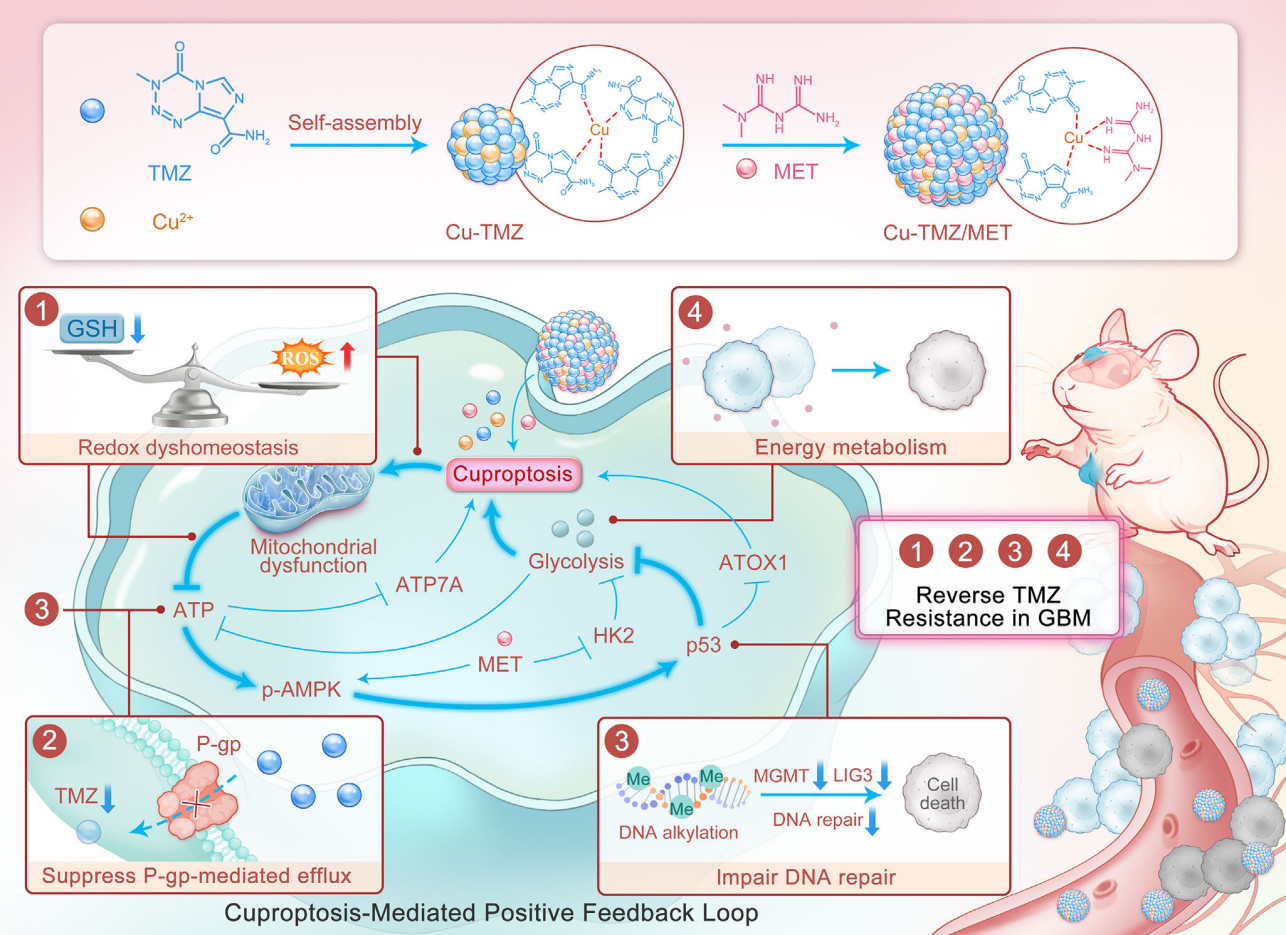
Preparation and mechanism of Cu‐TMZ/MET prodrugs. Schematic illustration of the preparation of Cu‐TMZ/MET prodrugs and the mechanism of reversing TMZ resistance based on cuproptosis‐mediated positive feedback loop. Abbreviations: temozolomide (TMZ), metformin (MET), glutathione (GSH), reactive oxygen species (ROS), adenosine triphosphate (ATP), phosphorylated AMP‐activated protein kinase (p‐AMPK), ATPase copper transporting alpha (ATP7A), antioxidant 1 (ATOX1), hexokinase‐2 (HK2), P‐glycoprotein (P‐gp), O^6^‐methylguanine‐DNA methyltransferase (MGMT), DNA ligase III (LIG3), and glioblastoma (GBM).

As a proof‐of‐concept of leveraging cuproptosis‐mediated positive feedback loop to reverse TMZ resistance in GBM, we engineered copper‐TMZ (Cu‐TMZ) prodrugs via coordination‐driven assembly of cupric ion (Cu^2+^) with TMZ (Scheme 1). In the GBM tumor microenvironment (TME), these prodrugs exhibit GSH‐responsive behavior, depleting intracellular GSH while releasing Cu^+^ and TMZ. The liberated Cu^+^ induces mitochondrial proteotoxic stress and initiates cuproptosis, while TMZ exerts its established DNA‐damaging effect. Importantly, cuproptosis further exacerbates mitochondrial dysfunction, suppresses ATP production, and activates AMPK. AMPK activation upregulates p53, shifting tumor metabolism away from glycolysis toward oxidative phosphorylation and thereby increasing susceptibility to cuproptosis. Glycolysis inhibition further aggravates ATP depletion and strengthens the feedback loop. To amplify this loop, we incorporated metformin (MET), a clinically approved antidiabetic drug and well‐characterized AMPK activator, with Cu‐TMZ to form Cu‐TMZ/MET prodrugs. MET additionally suppresses glycolysis by inhibiting hexokinase‐2 (HK2), and AMPK‐mediated p53 activation downregulates ATOX1, facilitating Cu^+^ retention. Concurrent downregulation of ATP7A further impairs copper efflux, sustaining intracellular copper overload and reinforcing cuproptosis. As a result, TMZ resistance is significantly reversed, and tumor progression is markedly suppressed in cell‐derived xenograft (CDX) models.

## 2 Results and Discussion

### 2.1 Characterization of Prodrugs

**FIGURE 1:**
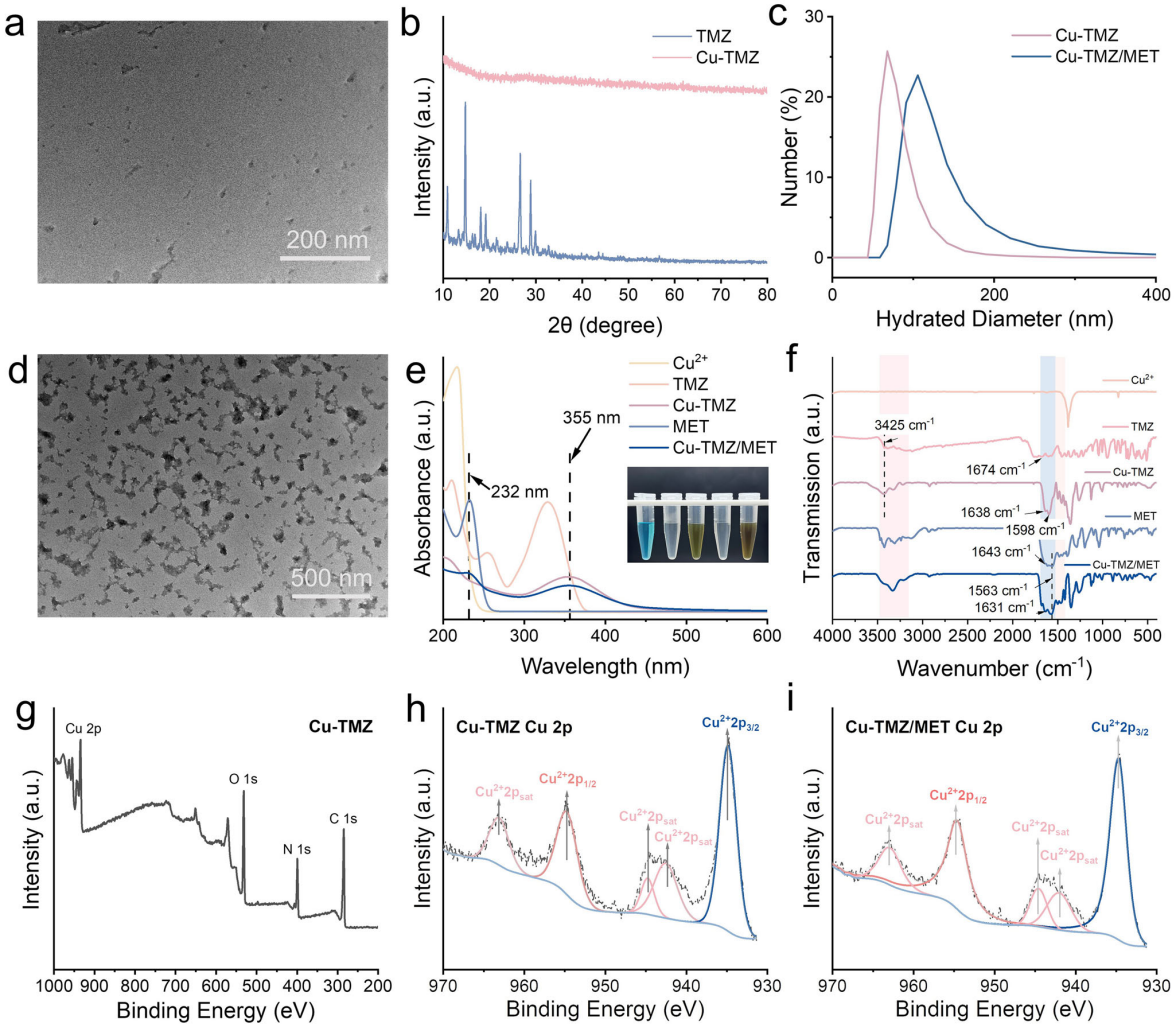
Characterization of prodrugs. (a) TEM image of Cu‐TMZ prodrugs. (b) XRD patterns of TMZ and Cu‐TMZ prodrugs. (c) Hydrated diameters of Cu‐TMZ prodrugs and Cu‐TMZ/MET prodrugs. (d) TEM image of Cu‐TMZ/MET prodrugs. (e) UV–vis absorption spectra of Cu(NO_3_)_2_, TMZ, Cu‐TMZ prodrugs, MET, and Cu‐TMZ/MET prodrugs. Inset in Figure 1e: photographs of the solutions of Cu(NO_3_)_2_, TMZ, Cu‐TMZ prodrugs, MET, and Cu‐TMZ/MET prodrugs from left to right. (f) FTIR spectra of Cu(NO_3_)_2_, TMZ, Cu‐TMZ prodrugs, MET, and Cu‐TMZ/MET prodrugs. XPS survey spectrum of Cu‐TMZ prodrugs (g), XPS Cu 2p spectrum of Cu‐TMZ prodrugs (h), and Cu‐TMZ/MET prodrugs (i).

As described in the experimental section, Cu‐TMZ prodrugs are prepared through the assembly of Cu^2+^ and TMZ. The as‐prepared Cu‐TMZ appears as a quasi‐spherical morphology with an average diameter of 10.3 nm and displays an amorphous structure (Figure 1a,b). Dynamic light scattering (DLS) measurement reveals that Cu‐TMZ possesses an average hydrodynamic diameter of 72.4 nm (Figure 1c). Comparative analysis of the UV–vis absorption spectra of TMZ and Cu‐TMZ demonstrates the emergence of a new absorption peak at ∼355 nm arising from a charge‐transfer transition from TMZ to Cu^2+^, accompanied by a pronounced tailing effect in the long‐wavelength region (Figure 1e). Visually, the Cu^2+^ solution appears light blue, and the TMZ solution is colorless, while the Cu‐TMZ prodrug appears as a colloidal dispersion with a yellowish‐green hue (Figure 1e inset). Furthermore, Fourier‐transform infrared (FTIR) spectroscopy of Cu‐TMZ exhibits characteristic bands at 3500–3100 and 1598 cm^−1^, corresponding to the N─H stretching vibration and N─H in‐plane bending vibration of TMZ, respectively. The observed redshift of the C═O stretching vibration from 1674 to 1638 cm^−1^, accompanied by the shift of C═N vibrational bands from 1400–1450 to 1450–1500 cm^−1^, provides strong evidence for the coordination between Cu^2+^ and TMZ (Figure 1f). The elemental composition of Cu‐TMZ is characterized by X‐ray photoelectron spectroscopy (XPS). The survey spectrum confirms the presence of Cu, O, N, and C (Figure 1g). The Cu 2p spectrum reveals that the copper is in the form of Cu(II) (Figure 1h). Control experiments indicate that the optimal Cu‐TMZ prodrugs, in terms of composition, morphology, and size, are identified as the preparation with 3.0 mM Cu(NO_3_)_2_, 1.0 mM TMZ, and 2.0 mM NaOH at 40 °C for 4 h (Figures S1–S4 and Table S1). Combining the results of inductively coupled plasma‐optical emission spectrometer (ICP‐OES) and elemental analysis, the optimal Cu‐TMZ prodrugs contain 40.3% Cu, 20.2% N, 15.7% C, 2.5% H, and 21.3% O. The corresponding Cu‐to‐TMZ molar ratio is calculated as 2.6:1 (Table S1). These results provide additional evidence for the successful preparation of Cu‐TMZ prodrugs.

Subsequently, MET is incorporated into Cu‐TMZ through the coordination of Cu^2+^ with the guanidino group to prepare Cu‐TMZ/MET prodrugs. UV–vis absorption spectrum reveals the appearance of a characteristic peak of MET at ∼232 nm, confirming its successful loading (Figure 1e). In comparison to Cu‐TMZ, a size increase of Cu‐TMZ/MET to 31.9 nm is revealed by transmission electron microscopy (TEM) (Figure 1d and Figure S5). DLS measurement indicates an increase in hydrodynamic diameter to 105.9 nm (Figure 1c). Further characterization by FTIR confirms the successful preparation of Cu‐TMZ/MET prodrugs, as evidenced by the appearance of C═N stretching vibration bands of the guanidino group at 1690–1500 cm^−1^ (Figure 1f). The XPS spectra clearly demonstrate that the copper remains in the form of Cu(II) after the introduction of MET (Figure 1i and Figure S6). The elemental composition of Cu‐TMZ/MET prodrugs is determined by elemental analysis and ICP‐OES measurements, showing 29.6% Cu, 28.1% N, 21.5% C, 3.8% H, and 17.0% O, and an approximate Cu‐to‐TMZ‐to‐MET mass ratio of 1:1.2:1.2 (Table S2). The corresponding parameters of Cu‑TMZ and Cu‑TMZ/MET prodrugs are summarized in Table S3. Colloidal stability is evaluated by monitoring size evolution over 7 days using DLS. Both Cu‐TMZ and Cu‐TMZ/MET exhibit slight fluctuations in size, indicating good stability (Figure S7).

To reveal the driving forces for maintaining the nanostructure, Cu‐TMZ/MET prodrugs are incubated with different interaction‐disrupting reagents (Figure S8). Upon addition of the chelating agent disodium ethylenediaminetetraacetate (EDTA), a coordination interaction disruptor, the solution immediately becomes colorless, suggesting rapid disassembly. Over time, the solution gradually turns blue, likely due to coordination between EDTA and Cu^2+^. Significant color changes and precipitation are also observed in the presence of Triton X‐100, a hydrophobic interaction disruptor, and NaCl, an electrostatic interaction disruptor, indicating disassembly. UV–vis absorption spectra and DLS measurements show that the blank group, DMSO‐treated group, and urea‐treated group maintain the spectral profiles and hydrodynamic diameters. In contrast, the NaCl‐treated group exhibits attenuated absorption at 355 nm and a tenfold increase in hydrodynamic diameter, suggesting aggregation. Both Triton X‐100 and EDTA treatments result in the disappearance of characteristic absorption peaks and alteration of hydrodynamic diameters, consistent with the TEM observations. Collectively, the formation of Cu‐TMZ/MET prodrugs is primarily driven by Cu^2+^‐mediated coordination interaction, which also involves electrostatic and hydrophobic interactions.

### 2.2 GSH Depletion and ROS Production

The TME is featured by a high GSH concentration of around 1–10 mM, which plays a critical role in maintaining redox homeostasis [50, 51]. Due to the existence of coordinated Cu^2+^, the GSH‐depletion behavior of prodrugs is investigated using O‐phthalaldehyde (OPA) as an indicator. OPA can react with GSH to generate benzothiazole derivatives, which exhibit bright blue fluorescence at 420 nm and are detectable by fluorescence spectra (Figure S9). No fluorescence signal is detected for the solutions containing OPA alone or a mixture of Cu‐TMZ/MET and GSH. Upon mixing OPA and GSH, a distinct fluorescence signal is detected, and the intensity at 420 nm gradually decreases with increasing prodrug concentration, indicating GSH depletion. The rapid disassembly in the presence of GSH and complete degradation within 2 h are further confirmed by TEM (Figure S10). To analyze the degradation products, neocuproine, a Cu^+^‐specific chelator that forms a yellow complex with Cu^+^, is used. When they are co‐incubated with GSH, a significant increase in absorbance at 455 nm is observed, confirming the release of Cu^+^ (Figure S11). This means that, accompanied by GSH depletion, the coordinated Cu^2+^ is reduced to Cu^+^ by GSH. Additionally, the cumulative release of TMZ gradually increases over time, reaching a plateau at ∼4 h, whereas negligible TMZ release is detected in the absence of GSH (Figure S12). These results demonstrate that Cu‐TMZ/MET exhibits GSH‐responsive behavior, effectively depleting GSH, undergoing disassembly, and releasing Cu^+^ along with TMZ.

In comparison to Cu^2+^, Cu^+^ exhibits significantly higher catalytic activity in the Fenton‐like reaction, enabling the conversion of H_2_O_2_ into highly reactive ·OH and exacerbating redox dyshomeostasis. To confirm this, the ·OH production ability of Cu‐TMZ/MET is investigated using terephthalic acid (TPA) and methylene blue (MB) as indicators. Experimental results show that ·OH is produced only after the simultaneous treatment with Cu‐TMZ/MET, GSH, and H_2_O_2_ (Figure S13). These results demonstrate the potential of prodrugs for triggering a Fenton‐like reaction to produce ·OH under the TME with high concentrations of GSH and H_2_O_2_, possessing the ability to induce redox dyshomeostasis (Figure S14).

### 2.3 Cuproptosis and Cuproptosis‐Mediated Positive Feedback Loop

**FIGURE 2:**
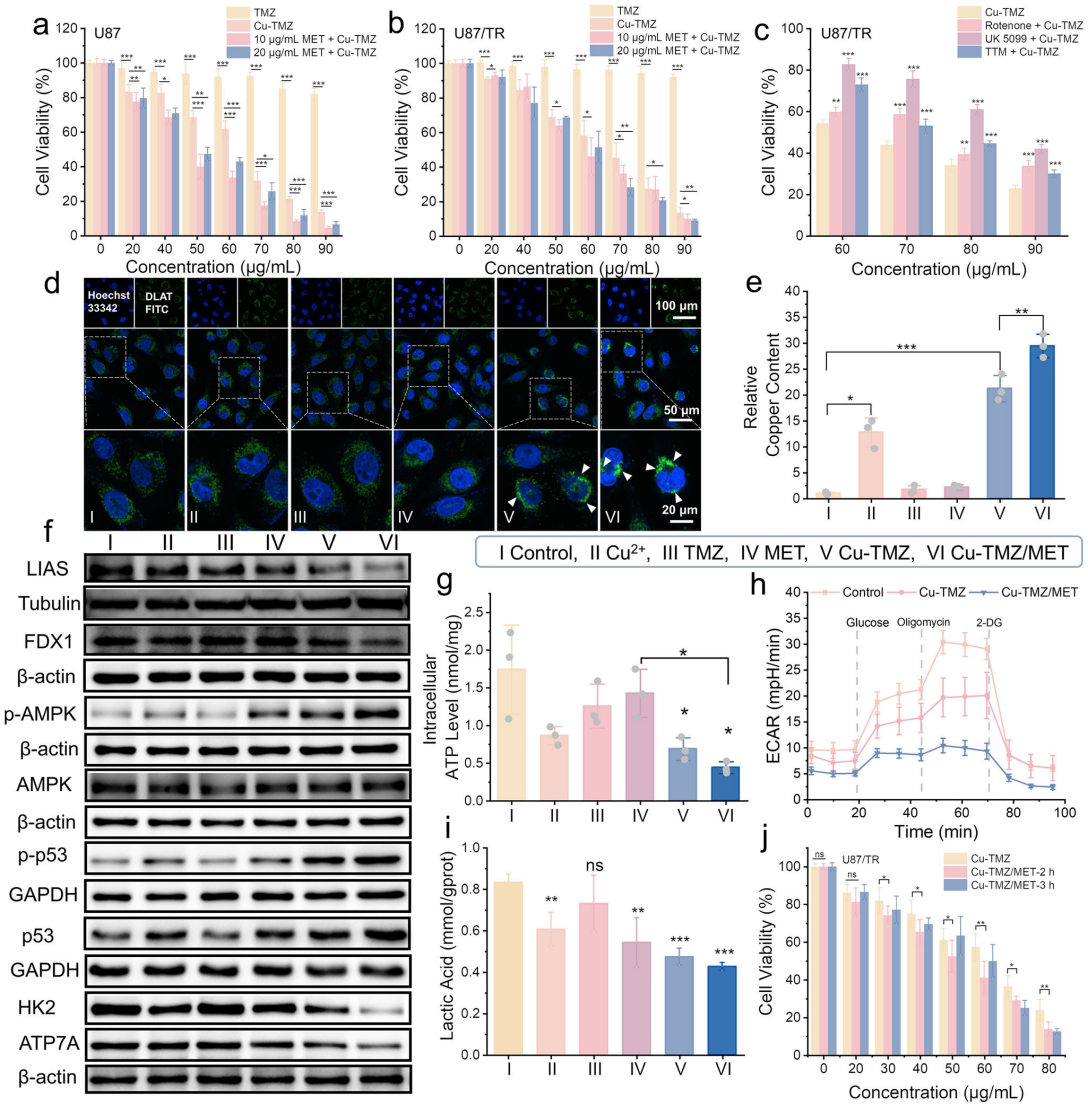
Cuproptosis and cuproptosis‐mediated positive feedback loop in vitro. Viability of U87 (a) and U87/TR (b) cells incubated with TMZ, Cu‐TMZ prodrugs, Cu‐TMZ prodrugs plus 10 or 20 µg/mL MET (n = 5). (c) Viability of U87/TR cells pretreated with the indicated compounds, including rotenone, UK 5099, or TTM, and then treated with Cu‐TMZ prodrugs. (d) The CLSM images of DLAT (pseudocolor: green) in U87/TR cells after the indicated treatments. (e) Relative copper content in U87/TR cells after indicated treatments (n = 3). (f) The expression levels of LIAS, FDX1, p‐AMPK, AMPK, p‐p53, p53, HK2, and ATP7A in U87/TR cells by WB analysis after the indicated treatments. (g) Intracellular ATP level of U87/TR cells after indicated treatments (n = 3). (h) ECAR curve of U87/TR cells after indicated treatments (n = 5). (i) Lactic acid content of U87/TR cells after indicated treatments (n = 6). (j) Viability of U87/TR cells incubated with Cu‐TMZ and Cu‐TMZ/MET prodrugs (n = 5). Cu‐TMZ/MET‐2 h and Cu‐TMZ/MET‐3 h refer to the duration of 2 and 3 h for loading MET into Cu‐TMZ prodrugs, respectively. Data are presented as mean ± SD. Statistical significance is calculated via a one‐way analysis of variance (ANOVA) test. ^*^p< 0.05, ^**^p< 0.01, and ^***^p< 0.001, ns, not significant.

The cytotoxicity of Cu‐TMZ prodrugs is evaluated using the CCK‐8 method, which shows significant killing effects against U87 GBM cells. At a concentration of 50 µg/mL, the equivalent TMZ concentration is 23.4 µg/mL, and cell viability is reduced to 68.5%, while at 90 µg/mL, cell viability is further reduced to 13.5% (Figure 2a, Figure S15, and Table S4). Even at 90 µg/mL, free TMZ still maintains 81.1% cell viability and shows a higher IC50 of 224.2 µg/mL. The markedly stronger antitumor activity of the prodrugs demonstrates that our prodrug strategy can effectively overcome the therapeutic limitations of TMZ monotherapy. Notably, the prodrugs also exhibit strong efficacy against TMZ‐resistant U87 (U87/TR) cells, with viabilities of 68.8% and 13.3% at 50 and 90 µg/mL, respectively (Figure 2b and Figure S15, and Table S5). In contrast, the treatment with an equivalent dose of TMZ alone results in a much higher survival rate (90 µg/mL, 92.2%), indicating that Cu‐TMZ prodrugs effectively reverse TMZ resistance and enhance GBM cell‐killing effect.

Since aberrant intracellular copper accumulation is known to induce cuproptosis, the potential cuproptosis‐related mechanism of Cu‐TMZ prodrugs is investigated. Previous studies have shown that as mitochondrial respiration inhibitors, rotenone and UK 5099 can suppress cuproptosis [21, 52]. Thus, U87 and U87/TR cells are pre‐treated with UK 5099 or rotenone (Figure 2c and Figure S16, and Table S6). The results reveal that the cytotoxicity of prodrugs is significantly reduced by these inhibitors, suggesting that mitochondrial respiration plays a critical role in the prodrug‐induced cell death. Furthermore, the treatment with the copper chelator tetrathiomolybdate (TTM) effectively rescues the prodrug‐induced cell death for both U87 and U87/TR cells, confirming the dependence of prodrug cytotoxicity on intracellular copper accumulation. These findings suggest that the Cu‐TMZ prodrugs may reverse TMZ resistance by inducing cuproptosis in GBM cells.

Regarding the process of cuproptosis, intracellular copper directly binds to the lipoylated proteins in mitochondria, resulting in the abnormal aggregation of DLAT and the loss of Fe‐S cluster proteins, triggering proteotoxic stress. Therefore, the DLAT oligomer, a key marker of cuproptosis, is analyzed by confocal laser scanning microscopy (CLSM) (Figure 2d). Compared to the Control group, a slight increase in DLAT oligomerization is observed in the Cu^2+^‐only treatment group, whereas more significant DLAT aggregation is detected in U87/TR cells after Cu‐TMZ treatment. Furthermore, the loss of Fe‐S cluster‐related proteins is confirmed by western blotting (WB) analysis, revealing significantly reduced protein levels of ferredoxin 1 (FDX1) and lipoic acid synthetase (LIAS) for Cu‐TMZ‐treated U87/TR cells, which provides definitive evidence of cuproptosis induction (Figure 2f).

Cuproptosis triggers severe mitochondrial dysfunction, characterized by the collapse of membrane potential and ATP depletion, due to copper‐induced proteotoxicity in the TCA cycle [53, 54, 55]. To determine whether Cu‐TMZ prodrugs induce mitochondrial dysfunction, mitochondrial membrane potential is measured using JC‐1 staining (Figure S17). While no significant changes are observed for the cells treated with Cu^2+^ or TMZ alone, the Cu‐TMZ treatment leads to a marked increase in green fluorescence (JC‐1 monomers), and a corresponding decrease in red fluorescence (JC‐1 aggregates), indicating mitochondrial depolarization and severe mitochondrial damage. Consistently, ATP production is significantly reduced in the Cu‐TMZ group, confirming mitochondrial dysfunction‐induced energy metabolism impairment (Figure 2g). This ATP depletion activates the AMPK pathway, as evidenced by the increased AMPK phosphorylation in WB analysis (Figure 2f). Activated AMPK subsequently opposes the Warburg effect and upregulates p53 expression, which promotes metabolic reprogramming from glycolysis to oxidative phosphorylation (Figure 2f). Glycolysis stress tests reveal attenuated extracellular acidification rate (ECAR) elevation in Cu‐TMZ‐treated cells, indicating partial glycolysis inhibition (Figure 2h). This metabolic shift may sensitize cuproptosis. Concurrently, these findings verify that Cu‐TMZ prodrugs induce a self‐amplifying positive feedback loop wherein cuproptosis‐triggered mitochondrial dysfunction and reduced ATP levels activate AMPK signaling, upregulate p53, and inhibit glycolysis, ultimately reinforcing cellular sensitivity to cuproptosis. Moreover, glycolysis inhibition further reduces ATP levels, amplifying the cuproptosis‐mediated positive feedback loop.

Although MET alone exhibits negligible cytotoxicity, the addition of MET significantly enhances the toxicity of Cu‐TMZ toward U87 cells and U87/TR cells (Figure 2a,b and Figure S15 and Tables S4 and S5). This result suggests that the incorporation of MET may allow a moderate reduction in TMZ dosage while maintaining comparable therapeutic efficacy, which strengthens the potential of TMZ‐mediated GBM therapy, implying that MET may cooperatively boost the cuproptosis‐mediated positive feedback loop. On this basis, an optimized formulation is developed by loading MET into Cu‐TMZ to produce Cu‐TMZ/MET prodrugs, which exhibit superior cytotoxicity against U87 and U87/TR cells compared to Cu‐TMZ (Figure 2j and Figure S18, and Tables S7 and S8). The loading of MET on Cu‐TMZ is confirmed by the UV–vis absorption spectrum according to the characteristic peak at ∼232 nm, which also indicates the highest loading efficiency after a 2 h duration (Figure S18). Thus, the Cu‐TMZ/MET prodrugs prepared with the aforementioned 2 h formulation are adopted for further investigations. Notably, both Cu‐TMZ and Cu‐TMZ/MET prodrugs exhibit excellent selectivity, with significantly lower toxicity toward normal brain‐derived endothelial (bEnd.3) cells and L929 mouse fibroblast cells but higher toxicity toward U87/TR cells (Figure S19). Such selectivity is attributed to the GSH‐responsive release behavior of prodrugs, given the relatively high GSH levels in tumor cells. This different GSH concentration is critical for our prodrug design, as it enables tumor‐selective activation, thereby ensuring favorable antitumor efficacy while minimizing potential toxicity toward normal brain tissue in vivo.

Localization analysis is conducted to investigate the cellular internalization and endosomal escape capability of Cu‐TMZ/MET. FITC is loaded on Cu‐TMZ/MET prodrugs for intracellular observation. It is shown that they demonstrate efficient cellular internalization, as evidenced by significantly enhanced green fluorescence within cells. The presence of discrete fluorescent puncta further indicates partial lysosomal escape. Both effective internalization and lysosomal escape contribute to improved therapeutic efficacy of Cu‐TMZ/MET (Figure S20). To further validate the potent cytotoxic effect of the treatment, calcein‐AM/PI staining is performed. The Cu‐TMZ/MET‐treated group exhibits the highest proportion of PI‐positive (red fluorescence) cells, confirming its effective killing capability against TMZ‐resistant GBM (Figure S21).

Since MET is an AMPK activator, the prodrugs may further promote the cuproptosis‐mediated positive feedback loop after loading MET. Compared to Cu‐TMZ, Cu‐TMZ/MET prodrugs exhibit increased AMPK phosphorylation levels, supporting the role of MET in AMPK activation (Figure 2f). In addition, inhibition of AMPK using an AMPK inhibitor, dorsomorphin, significantly rescues Cu‐TMZ/MET‐induced cell death (Figure S22), which clearly demonstrates that the observed effects of MET are primarily mediated through AMPK activation. Downstream of AMPK activation, p53 expression is upregulated in the Cu‐TMZ/MET‐treated group (Figure 2f), which contributes to glycolysis inhibition, mitochondrial metabolic reprogramming, and an enhanced TCA cycle, ultimately sensitizing cells to cuproptosis. Additionally, MET suppresses the expression of HK2, a key glycolytic enzyme, further disrupting glycolysis (Figure 2f). This glycolysis inhibition is confirmed by decreased ATP levels (Figure 2g), reduced lactate production (Figure 2i), and attenuated ECAR elevation (Figure 2h) in Cu‐TMZ/MET‐treated cells. The impaired glycolytic flux leads to ATP depletion, resulting in the downregulation of ATP7A. WB analysis reveals ATP7A suppression in the Cu‐TMZ group, with more significant suppression in the Cu‐TMZ/MET group, indicating that MET enhances intracellular copper retention and potentiates cuproptosis (Figure 2f). Moreover, p53‐mediated downregulation of ATOX1 further blocks copper efflux, reinforcing copper accumulation (Figure S23). Inductively coupled plasma‐mass spectrometry (ICP‐MS) analysis confirms that the combination of copper ions and TMZ in Cu‐TMZ substantially raises intracellular copper content, while MET further enhances this accumulation (Figure 2e). Furthermore, as evidenced by reduced levels of Fe‐S cluster‐related proteins (FDX1 and LIAS) (Figure 2f), increased DLAT oligomerization (Figure 2d), and further mitochondrial dysfunction shown by the collapse of mitochondrial membrane potential (Figure S17), cuproptosis is exacerbated in the Cu‐TMZ/MET group. These findings collectively demonstrate that the loading of MET into Cu‐TMZ enhances the cuproptosis‐mediated positive feedback loop, improving therapeutic efficacy against TMZ‐resistant GBM.

### 2.4 Reversing TMZ Resistance by Cuproptosis‐Mediated Positive Feedback Loop

**FIGURE 3:**
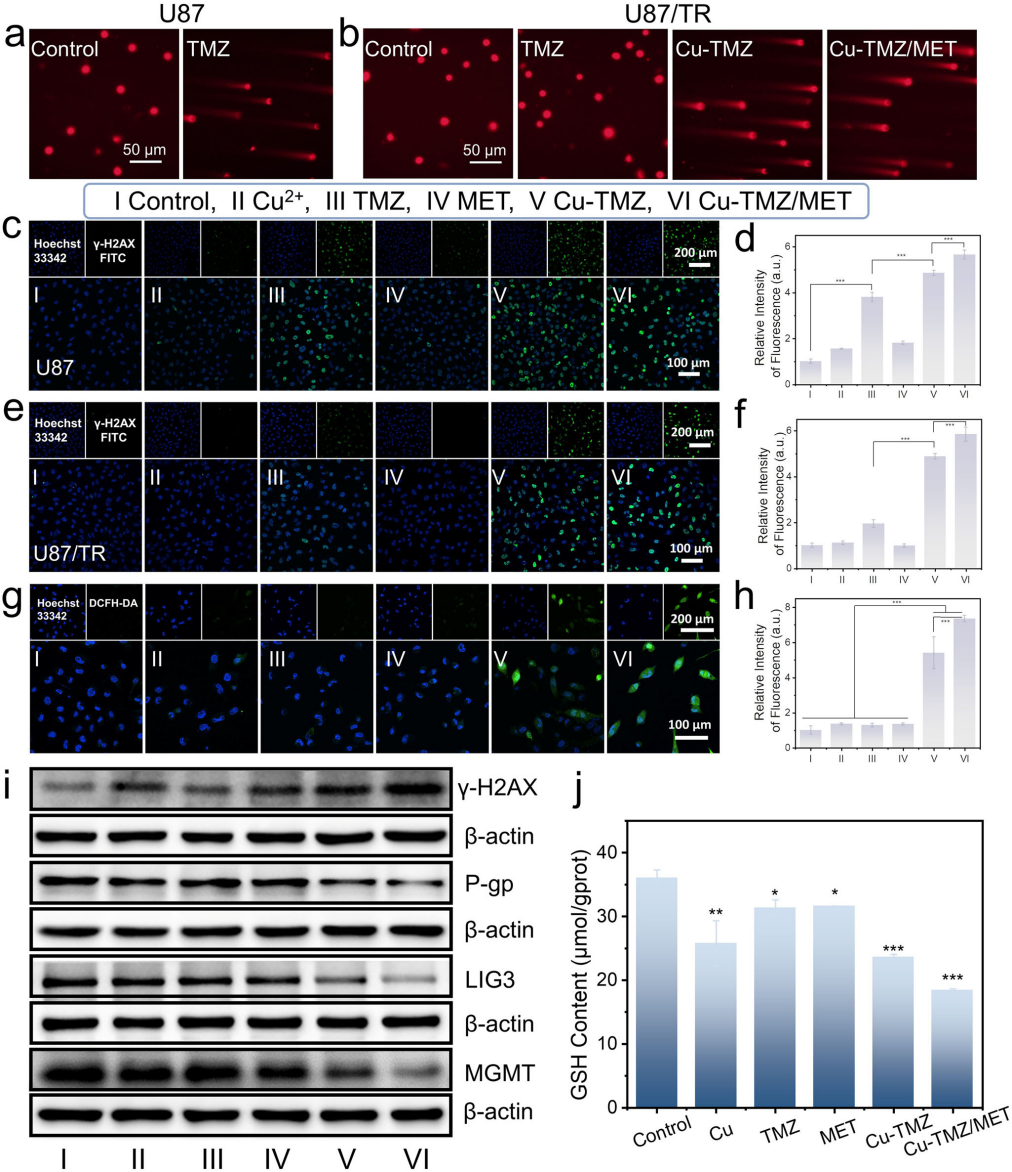
Reversing TMZ resistance in vitro. Neutral comet assay of U87 (a) and U87/TR (b) cells after indicated treatments. The CLSM images of γ‐H2AX (pseudocolor: green) in U87 (c) and U87/TR (e) cells after the indicated treatments. The quantitative analysis of U87 (d) and U87/TR (f) cells stained with FITC‐labeled secondary antibody representing the level of γ‐H2AX (n = 3). The CLSM images (g) and quantitative analysis (h) of U87/TR cells after staining with DCFH‐DA in different groups (n = 3). (i) The expression levels of γ‐H2AX, P‐gp, LIG3, and MGMT in U87/TR cells by WB analysis after indicated treatments. (j) Intracellular GSH content of U87/TR cells after indicated treatments (n = 4). Data are presented as mean ± SD. Statistical significance is calculated via a one‐way ANOVA test. ^*^p< 0.05, ^**^p< 0.01, and ^***^p< 0.001.

It is well established that TMZ exerts its cytotoxic effects on GBM by inducing DNA damage. For detecting DNA double‐strand breaks, the comet tail in the neutral comet assay is sensitive evidence. As shown in Figure 3a, treatment of U87 cells with TMZ induces comet tails, revealing DNA damage. The percentage of tail DNA increases from 5.3% in Control cells to 37.2% after TMZ treatment (Figure S24a). However, when U87/TR cells are treated with TMZ, no significant comet tail is observed, confirming the absence of DNA damage in the resistant cells (Figure 3b). Upon treatment with Cu‐TMZ, a substantial number of cells exhibit comet tails, accompanied by an increase in tail DNA to 80.0% (Figure S24b). Importantly, this effect is even more pronounced with Cu‐TMZ/MET, as reflected by a notably higher tail DNA percentage of approximately 94.0%, indicating enhanced DNA damage. Moreover, immunofluorescence staining of γ‐H2AX, a widely recognized biomarker of DNA double‐strand breaks, demonstrates significantly enhanced green fluorescence in TMZ‐treated U87 cells compared to the Control group (Figure 3c,d). While the DNA damage signal is attenuated for U87/TR cells, indicative of the TMZ‐resistant mechanism (Figure 3e,f). Notably, treatment with Cu‐TMZ increases both the intensity and proportion of γ‐H2AX‐positive signals, which are further enhanced using Cu‐TMZ/MET. These findings are corroborated by WB analysis of U87/TR cells (Figure 3i), confirming that Cu‐TMZ and Cu‐TMZ/MET potentiate TMZ‐induced DNA damage and particularly reverse TMZ resistance in GBM.

To further clarify the mechanism of our prodrugs for reversing TMZ resistance, cell cycle distribution in U87/TR cells is analyzed by flow cytometry (Figure S25). Treatment with Cu‐TMZ prodrugs induces pronounced G2/M phase arrest, which is further enhanced by Cu‐TMZ/MET prodrugs, indicating that our prodrugs strongly disrupt cell cycle progression in TMZ‐resistant cells. Correspondingly, WB analysis shows significantly elevated levels of p21 and p‐CDK1, along with reduced Cyclin B1 expression, molecularly confirming the induction of G2/M arrest (Figure S26). Such abnormal G2/M cell cycle progression demonstrates that our cuproptosis nanoprodrugs can effectively reverse TMZ resistance and restore chemosensitivity of resistant GBM.

Previous reports suggest that mitochondrial dysfunction and elevated oxidative stress can sensitize resistant GBM cells to TMZ [10, 35, 36]. Since Cu‐TMZ and Cu‐TMZ/MET can cause mitochondrial dysfunction (Figure S17 and Figure 2g), their capability to disrupt redox homeostasis is subsequently evaluated. As revealed by Figure 3j, a significant depletion of GSH in Cu‐TMZ‐treated cells is found, which is further depleted in Cu‐TMZ/MET‐treated cells, consistent with enhanced cuproptosis. ROS detection using the DCFH‐DA probe shows minimal fluorescence in the Control group [56], while Cu‐TMZ or Cu‐TMZ/MET treatments progressively intensify green fluorescence, indicating ROS induction (Figure 3g,h). These results align with in vitro validation (Figures S9 and S13), demonstrating that prodrugs disrupt intracellular redox homeostasis through the cuproptosis‐mediated positive feedback loop, thereby reversing TMZ resistance.

Additionally, the downregulation of ATP levels can suppress P‐gp expression, reduce drug efflux, and reverse TMZ resistance [38, 39]. WB analysis of U87/TR cells confirms significant P‐gp reduction in both Cu‐TMZ and Cu‐TMZ/MET groups (Figure 3i). This supports the conclusion that prodrugs effectively deplete ATP and inhibit P‐gp via the cuproptosis‐mediated positive feedback loop, restoring TMZ sensitivity. Furthermore, cuproptosis causes intracellular ATP depletion, which reduces the expression of ATP‐dependent LIG3 (Figure 3i), a key enzyme responsible for the ligation step in the BER pathway. Inhibition of LIG3 leads to incomplete repair of TMZ‐induced m^7^G and m^3^A lesions, thereby enhancing TMZ‐triggered DNA damage and cytotoxicity.

Meanwhile, MGMT, a critical DNA repair protein, contributes substantially to TMZ resistance in GBM. The accumulation of p53 protein has been shown to suppress MGMT expression. WB analysis demonstrates that U87/TR cells exhibit downregulated p53 and phosphorylated p53 (p‐p53) expression accompanied by upregulated MGMT levels compared with parental U87 cells, explaining the intrinsic TMZ resistance of resistant GBM cells (Figure S27). Figure 3i demonstrates marked MGMT downregulation in prodrug‐treated cells, validating that cuproptosis‐mediated p53 upregulation inhibits MGMT, thereby attenuating TMZ resistance. Notably, T98G cells are well‐known to be highly TMZ‐resistant. However, they carry mutant p53. The expression levels of p53 and p‐p53 in T98G cells are examined (Figure S28). It is found that T98G cells show high p53 expression but inactive p‐p53, making it difficult to activate the p53‐dependent signaling pathway. Although the treatment alters the expression levels of p53, p‐p53, and MGMT, these effects are far less prominent than those observed in U87/TR cells (Figure S29 and Figures 2f and 3i). Similarly, mild changes in cell cycle‐related regulatory proteins are detected in T98G cells, which are much weaker than the robust responses observed in U87/TR cells (Figures S26 and S29). Accordingly, the ability to reverse TMZ resistance is significantly attenuated in T98G cells. In contrast, treatment with our Cu‐TMZ/MET prodrugs at a concentration of 60 µg/mL reduces the cell viability of A172 cells to 44.2%, a value significantly lower than the 60.1% viability for Cu‐TMZ prodrugs (Figure S30). This result supports the applicability of our prodrugs to GBM models harboring wild‐type p53. These results indicate that the cuproptosis‐mediated positive feedback loop and the ability to reverse TMZ resistance are largely dependent on functional p53.

Moreover, GBM chemoresistance is closely associated with glycolysis and HK2 expression. As indicated in Figure 2f–i, prodrugs effectively suppress HK2 expression and disrupt glycolysis through the cuproptosis‐mediated positive feedback loop mechanism, ultimately enhancing TMZ sensitivity and reversing TMZ resistance.

The above discussion demonstrates that TMZ resistance can be reversed by addressing multiple resistance mechanisms via the cuproptosis‐mediated feedback loop combining mitochondrial dysfunction, redox dyshomeostasis, ATP level downregulation, p53 activation, and glycolysis inhibition.

### 2.5 Transcription Analysis in Reversing TMZ Resistance by Cuproptosis‐Mediated Positive Feedback Loop

**FIGURE 4:**
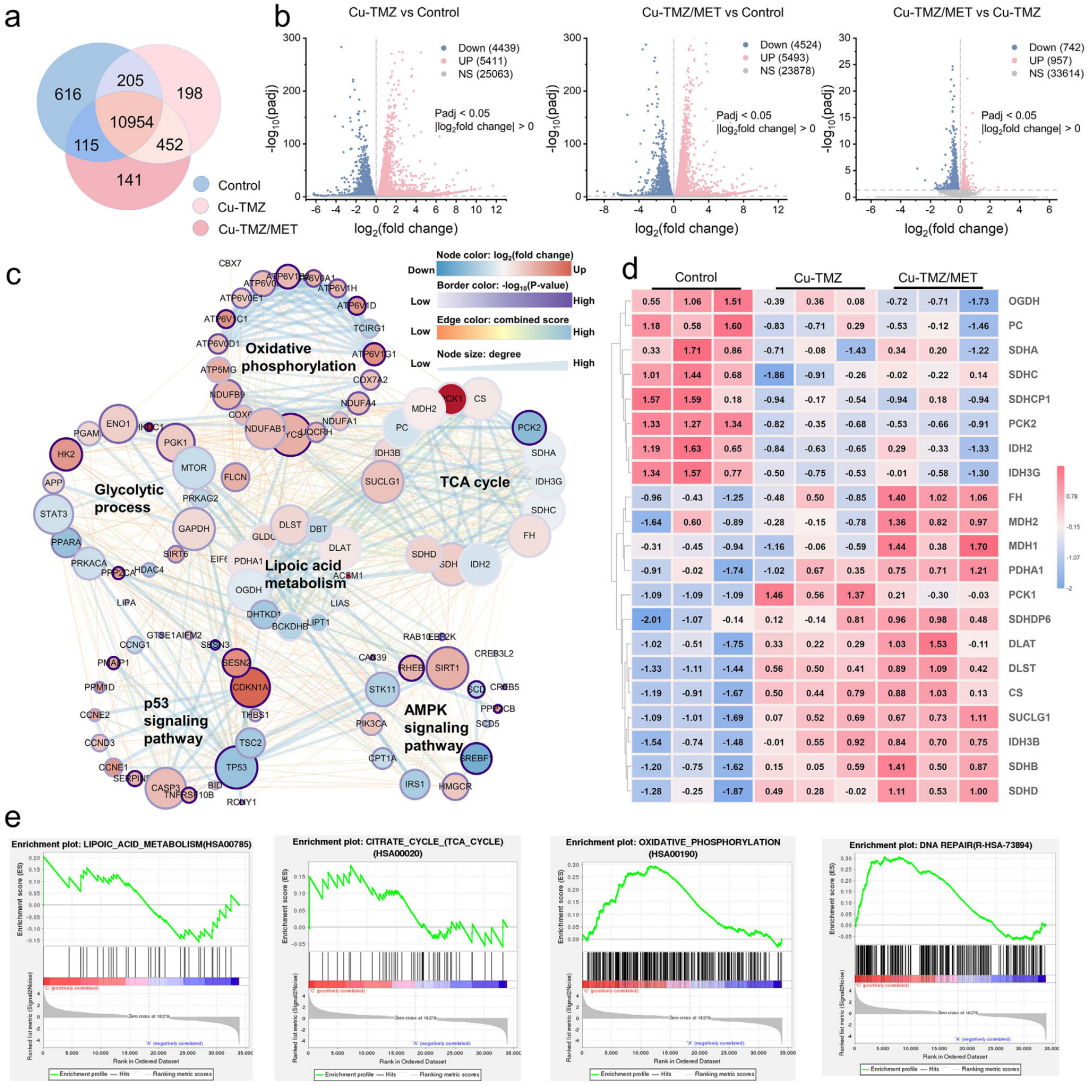
Transcription analysis. (a) A Venn diagram reveals the number of genes transcribed in Control, Cu‐TMZ, and Cu‐TMZ/MET prodrug groups. (b) Volcano plot of the distributions of differentially expressed genes after Cu‐TMZ or Cu‐TMZ/MET prodrug treatment (|log_2_fold change| > 0, padj < 0.05). No differentially expressed genes are denoted as NS. (c) The PPI of differentially expressed genes (Cu‐TMZ/MET vs Control) in the AMPK signaling pathway, p53 signaling pathway, glycolytic process, oxidative phosphorylation, TCA cycle, and lipoic acid metabolism in the STRING database. (d) Heat map analysis of differentially expressed genes in the TCA cycle pathway after Cu‐TMZ or Cu‐TMZ/MET prodrug treatment. Each grid displays the row z‐score value (n = 3). (e) GSEA analysis shows that the gene sets of the lipoic acid metabolism, TCA cycle, oxidative phosphorylation, and DNA repair pathway are enriched in Cu‐TMZ/MET prodrug‐treated cells.

To explore the pathway mechanism, gene expression analysis of prodrug‐treated U87/TR cells is performed using RNA‐sequencing. The transcriptional profile of 12681 genes is first analyzed (Figure 4a). Among these genes, 198 genes are transcribed only in cells treated with Cu‐TMZ, and 141 genes are transcribed only in cells treated with Cu‐TMZ/MET. Furthermore, compared to the Control group, the volcano plot illustrates that 5411 genes are upregulated (red dots), and 4439 genes are downregulated (blue dots) in cells treated with Cu‐TMZ, with the thresholds of padj < 0.05 and |log_2_fold change| > 0 (Figure 4b). However, 5493 genes are upregulated, and 4524 genes are downregulated in cells treated with Cu‐TMZ/MET. Moreover, compared to cells treated with Cu‐TMZ, cells treated with Cu‐TMZ/MET have 957 upregulated genes and 742 downregulated genes. Table S9 shows the top 50 differentially expressed genes for each contrast. As for the thresholds of |log_2_fold change| > 2 and *p* value < 0.05 (Figure S31), 1763 genes are upregulated, and 297 genes are downregulated in the Cu‐TMZ group. In addition, 1421 genes are upregulated, and 423 genes are downregulated in the Cu‐TMZ/MET group. Compared to the Cu‐TMZ group, cells treated with Cu‐TMZ/MET still have 66 upregulated genes and 193 downregulated genes. The Kyoto Encyclopedia of Genes and Genomes (KEGG) pathway analysis, Gene Ontology (GO) analysis, and Reactome analysis are conducted to examine the potential biological roles of the differentially expressed genes. Gene set enrichment analysis (GSEA), heat map, and protein–protein interaction (PPI) analysis of differentially expressed genes demonstrate that the cuproptosis‐related pathways, including the TCA cycle, lipoic acid metabolism, and mitochondrial iron sulfur cluster biogenesis, are enriched (Figure 4c–e and Figures S32–S34), contributing to the lipoylation of DLAT and the loss of Fe‐S cluster proteins. Subsequently, regulation of mitochondrial membrane potential and mitochondrial ATP synthesis‐coupled electron transport are enriched, indicating mitochondrial dysfunction and ATP production may be affected (Figures S35 and S36). After that, the AMPK signaling pathway is enriched (Figure S37). Activated AMPK subsequently opposes the Warburg effect and upregulates the p53 signaling pathway (Figure S38), which promotes metabolic reprogramming from glycolysis to oxidative phosphorylation. Figure 4e and Figures S39 and S40 show that the glycolytic process is enriched, and the oxidative phosphorylation pathway is significantly upregulated, which strengthens cuproptosis. The PPI analysis in these pathways shows close liaisons between the AMPK signaling pathway, the p53 signaling pathway, the glycolytic process, oxidative phosphorylation, the TCA cycle, and lipoic acid metabolism, highlighting the importance of the cuproptosis‐mediated self‐amplifying positive feedback loop (Figure 4c). Together, these analyses demonstrate that Cu‐TMZ induces a self‐amplifying positive feedback loop and Cu‐TMZ/MET strengthens the loop.

Chemoresistance in GBM is closely linked to redox homeostasis dysregulation. Enrichment analysis reveals significant alterations in chemical carcinogenesis‐ROS (Figure S41) and the GSH metabolism process (Figure S42), indicating redox dyshomeostasis. Additionally, the downregulation of ATP levels suppresses P‐gp expression, thereby reducing drug efflux and reversing TMZ resistance. This is further supported by the downregulation of the ABC transporters pathway (Figure S43). Metabolic adaptations, particularly the Warburg effect‐driven glycolytic dependency, sustain GBM proliferation and contribute to resistance. Metabolic reprogramming through the modulation of the glycolytic process can reverse TMZ resistance (Figure S39). Moreover, ATP depletion reduces the expression of LIG3, leading to incomplete repair of TMZ‐induced m^7^G and m^3^A lesions, thereby enhancing TMZ‐triggered DNA damage and cytotoxicity. Meanwhile, activation of the p53 signaling pathway suppresses MGMT expression, resulting in impaired repair of O^6^‐methylguanine and consequently exacerbating DNA alkylation damage. These mechanisms are supported by the significant upregulation of DNA damage bypass and DNA repair pathways (Figure 4e and Figures S44–S46). The PPI analysis in these pathways shows close liaisons between redox dyshomeostasis, mitochondrial dysfunction, glycolytic process, ABC transporters, p53 signaling pathway, and DNA repair (Figure S47). All these results demonstrate that Cu‐TMZ and Cu‐TMZ/MET prodrugs can reverse TMZ resistance and amplify DNA damage through a cuproptosis‐mediated positive feedback loop.

### 2.6 Therapeutic Evaluation in TMZ‐Resistant Subcutaneous GBM

**FIGURE 5:**
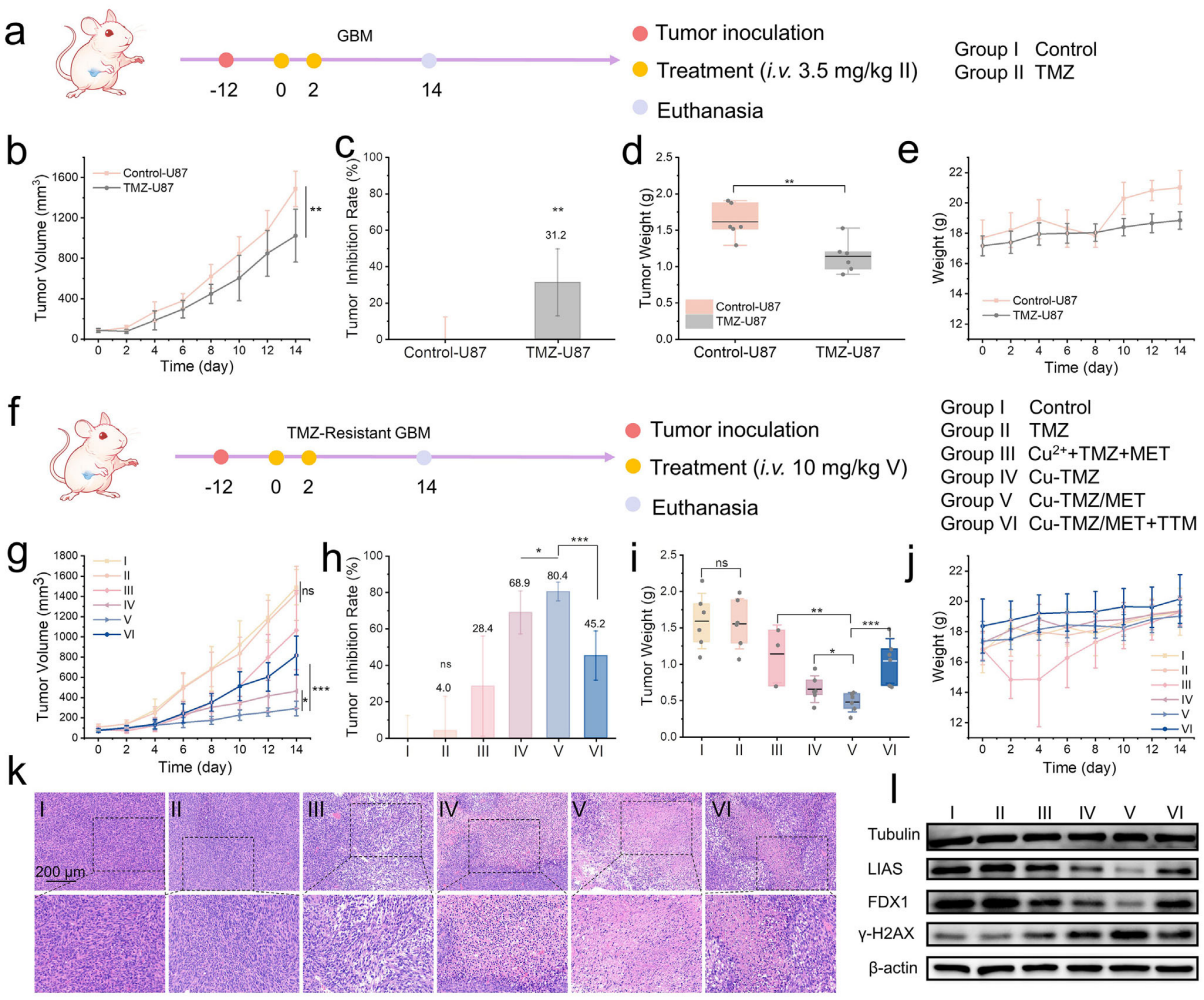
Therapeutic evaluation in TMZ‐resistant subcutaneous GBM. (a) The schematic illustration of U87 GBM‐bearing mice and the group setting. Animal grouping and data detection are randomized and blinded. The average tumor volume (b), average tumor inhibition (c), average tumor weight (d), and average body weight (e) of U87 GBM‐bearing mice in groups I and II (n = 6). (f) The schematic illustration of U87/TR GBM‐bearing mice and the group setting. The average tumor volume (g), average tumor inhibition (h), average tumor weight (i), and average body weight (j) of U87/TR GBM‐bearing mice in groups I–VI (n = 6). H&E‐stained tumor slice (k) of U87/TR bearing mice in groups I–VI. (l) The expression levels of LIAS, FDX1, and γ‐H2AX in tumor tissue by WB analysis in groups I–VI. Data are presented as mean ± SD in b, d, e, g, i, and j. Data are presented as mean ± confidence intervals (CI) in c and h. Statistical significance is calculated via a one‐way ANOVA test. ^*^p< 0.05, ^**^p< 0.01, and ^***^p< 0.001, ns, not significant.

To evaluate the in vivo therapeutic efficacy of prodrugs against TMZ‐resistant GBM, U87 and U87/TR GBM models are established in BALB/c Nude mice (Figure 5a,f). At 12 days post‐inoculation of U87/TR cells, mice are randomly divided into six groups: (I) Control, (II) TMZ, (III) Cu^2+^+TMZ+MET, (IV) Cu‐TMZ, (V) Cu‐TMZ/MET, and (VI) Cu‐TMZ/MET+TTM. Mice with U87 GBM models are randomly divided into two groups: (I) Control and (II) TMZ. All treatments are administered via tail vein injection. Several rationales support this unified intravenous delivery strategy. First, to control a single variable and ensure experimental rigor, both free TMZ and the nanoprodrugs are administered intravenously, although TMZ is conventionally administered orally. The use of a uniform delivery route minimizes confounding variables and allows an unbiased comparison of therapeutic efficacy. Second, free TMZ can degrade rapidly in vivo, which may weaken its antitumor activity after either intravenous or oral administration. In contrast, our prodrug possesses a stable nanostructure that resists premature breakdown in circulation and disassembles responsively inside tumor cells with high GSH levels to release the drugs, thereby explaining its enhanced antitumor potency. Third, oral delivery introduces additional confounding factors, such as gastrointestinal barriers, which may hinder an accurate potency comparison between free TMZ and the prodrug. Taken together, consistent intravenous administration is selected to enable a reliable comparative assessment.

Following the different treatments, tumor growth and body weight are monitored throughout the therapeutic intervention. Body weight of mice shows an overall increasing trend in groups I and II (Figure 5e,j). The tumor growth results demonstrate that the therapeutic efficacy of TMZ is significantly limited in U87/TR GBM, with a tumor inhibition rate of only 4.0%, compared to 31.2% in U87 GBM (Figure 5b–d,g–i and Figures S48 and S49). Notably, partial restoration of tumor suppression (28.4% inhibition) is achieved by treatment with the mixture of Cu^2+^, TMZ, and MET, suggesting that resistance to TMZ is sensitized through the induction of cuproptosis. However, severe biosafety concerns arise with this mixture, as the treatment group shows only 50% survival accompanied by precipitous weight loss (Figure 5j and Figure S49). In contrast, Cu‐TMZ induces robust tumor suppression in U87/TR GBM, with an inhibition rate of 68.9%. Cu‐TMZ/MET demonstrates even greater efficacy, achieving a tumor inhibition rate of 80.4%. Strikingly, co‐treatment with the copper chelator TTM in Group VI diminishes tumor suppression with the inhibition rate of 45.2%, further underscoring the pivotal role of the cuproptosis‐mediated positive feedback in reversing GBM chemoresistance. Moreover, no substantial body weight fluctuations are observed throughout the prodrug treatment period, demonstrating the excellent biosafety profile of the system (Figure 5j).

Histopathological analyses of GBM tissues at the experimental endpoint demonstrate that Cu‐TMZ/MET exhibits the most pronounced necrotic regions (Figure 5k), underscoring the superior anti‐TMZ‐resistant GBM efficacy. WB analysis confirms robust induction of cuproptosis‐related protein expression, with distinct DLAT oligomerization observed (Figure S50). Furthermore, Cu‐TMZ/MET treatment leads to significant downregulation of LIAS and FDX1, providing definitive evidence of the cuproptosis pathway activation (Figure 5l). Importantly, they strongly upregulate γ‐H2AX expression, establishing their ability to reverse GBM chemoresistance via the cuproptosis‐mediated positive feedback loop (Figure 5l).

### 2.7 Biodistribution and Therapeutic Evaluation in Orthotopic GBM

It is known that the blood–brain barrier (BBB) is typically disrupted and evolves into the blood–brain tumor barrier (BTB) during tumor progression, which exhibits increased permeability, thereby enabling the potential delivery of Cu‐TMZ/MET [57]. To investigate the accumulation capability in GBM, IR780‐labeled Cu‐TMZ/MET is intravenously administered to mice bearing orthotopic GBM. Fluorescence imaging reveals time‐dependent accumulation of prodrugs in the brain region, demonstrating excellent GBM‐targeting potential for treating orthotopic GBM (Figure S51). Moreover, the half‐life of Cu‐TMZ/MET is estimated at 2.7 h (Figure S52). The relatively long half‐life guarantees the efficacy for treating orthotopic GBM.

**FIGURE 6:**
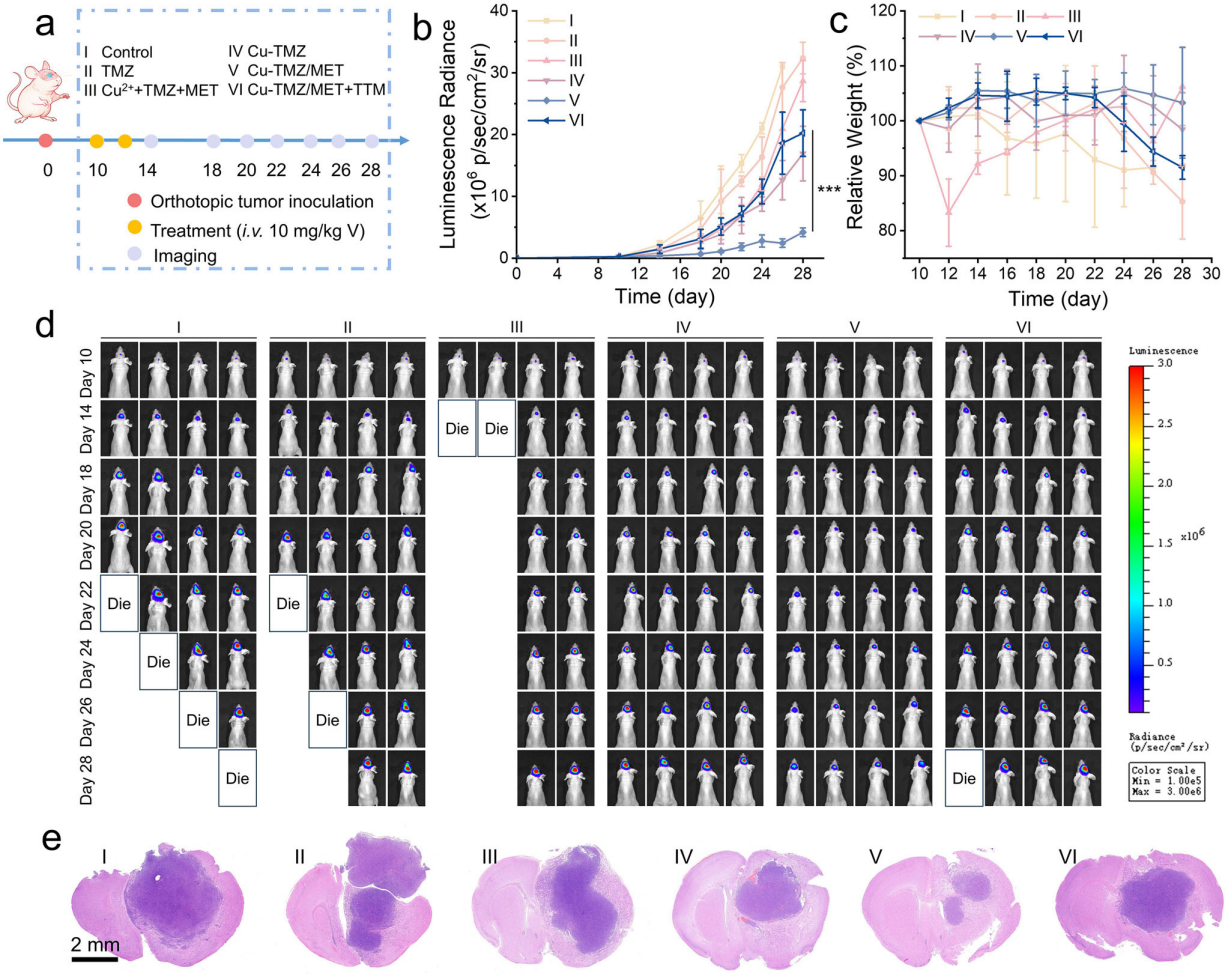
Antitumor efficacy of prodrugs in the orthotopic GBM model. (a) The schematic illustration of orthotopic GBM‐Luc‐bearing mice and the group setting. (b) IVIS bioluminescence intensity curve of GBM‐Luc‐bearing mice in groups I–VI (n = 4). (c) Relative body weight of GBM‐Luc‐bearing mice in groups I–VI (n = 4). (d) IVIS bioluminescence images of GBM‐Luc‐bearing mice in groups I–VI. (e) Representative whole‐brain H&E staining images of mice in groups I–VI. Data are presented as mean ± SD. Two‐way repeated measures ANOVA is used for Figure 6b statistical analysis. ^***^p< 0.001.

For therapeutic evaluation, an orthotopic GBM model is established by inoculating U87‐Luc cells (Figure 6a). The successful modeling of tumor‐bearing BALB/c Nude mice is confirmed by the In Vivo Imaging System (IVIS) on Day 10 after inoculation. Then, mice are randomly divided into six groups: (I) Control, (II) TMZ, (III) Cu^2+^+TMZ+MET, (IV) Cu‐TMZ, (V) Cu‐TMZ/MET, and (VI) Cu‐TMZ/MET+TTM. Following different treatments via tail vein injection, tumor growth is monitored by bioluminescence imaging throughout the therapeutic intervention. It shows a rapid tumor growth of mice in the Control group, and all mice die by Day 28, while the TMZ and the mixture administration group exhibit limited inhibitory effects (Figure 6b–d and Figure S53). The Cu‐TMZ group demonstrates obvious tumor suppression (41.5% of the control bioluminescence intensity on Day 26), with the Cu‐TMZ/MET group showing the most pronounced inhibition. On the 28th day, tumor‐bearing mice are euthanized, and brain tissues in each group are collected for histopathological analyses (Figure 6e). Whole‐brain H&E‐stained sections reveal that the tumor area in the Cu‐TMZ/MET group is the smallest, with tumor burden following the trend: Control > TMZ > Cu^2+^+TMZ+MET > Cu‐TMZ > Cu‐TMZ/MET, while Cu‐TMZ/MET+TTM > Cu‐TMZ/MET, confirming the superior anti‐tumor efficacy of prodrugs via the cuproptosis‐mediated positive feedback loop against orthotopic GBM.

### 2.8 Biosafety

To systematically evaluate the biosafety of prodrugs, the hemolysis assay, H&E staining of major organs, and serum biochemical analyses are performed. As shown in Figure S54, the hemolysis rate remains lower than 1% within the concentration range of 0 to 500 µg/mL, indicating good blood compatibility for intravenous injection. In addition, H&E staining of major organs, including heart, lungs, liver, spleen, and kidneys, reveals no significant histological abnormalities in the Cu‐TMZ and Cu‐TMZ/MET groups (Figure S55). Notably, no obvious histological damage is observed after intravenous administration of the prodrugs in brain tissue (Figure S56). Furthermore, the indices of liver function (ALT, AST, TBIL, DBIL, ALB, ALP, γ‐GT, and TBA) and kidney function (UREA, CREA, and UA) are in the normal range (Figure S57). In addition, no substantial body weight fluctuations are found throughout the prodrug treatment period in both subcutaneous and orthotopic GBM models (Figures 5j and 6c). All these results demonstrate the excellent biosafety profile of our prodrugs.

## 3 Conclusion

In summary, a cuproptosis‐mediated positive feedback loop is proposed to reverse TMZ resistance in GBM. Through the coordination‐driven assembly of Cu^2+^ and TMZ, a Cu‐TMZ prodrug is engineered. Regarding the positive feedback loop, the accumulation of copper facilitated by prodrugs induces mitochondrial proteotoxic stress, thus triggering cuproptosis. Cuproptosis initiates mitochondrial dysfunction, which suppresses ATP production and subsequently activates AMPK. After that, AMPK activation upregulates p53, shifting GBM metabolism from glycolysis toward oxidative phosphorylation. This metabolic reprogramming enhances cellular susceptibility to cuproptosis, while glycolysis inhibition further depletes ATP, reinforcing the cuproptosis‐mediated feedback loop. To strengthen the feedback loop, MET, an AMPK activator, is loaded into the prodrugs to prepare Cu‐TMZ/MET prodrugs. MET additionally suppresses glycolysis by inhibiting HK2 expression, while AMPK‐mediated p53 activation downregulates ATOX1, promoting intracellular Cu^+^ retention and sensitizing to cuproptosis. Thus, the TMZ resistance in GBM is significantly reversed in subcutaneous and orthotopic CDX models. Our work demonstrates that GBM therapy resistance can be reversed via a cuproptosis‐mediated feedback loop combining mitochondrial dysfunction, redox dyshomeostasis, ATP level downregulation, p53 activation, and glycolysis inhibition. This cuproptosis‐mediated feedback loop strategy has potential to simultaneously address multiple resistance mechanisms in chemotherapy‐resistant GBM, with great promise for clinical applications.

Our Cu‐TMZ/MET prodrugs construct a self‐amplifying cuproptosis feedback loop to reverse TMZ resistance, and this feedback loop relies on functional p53. For patients with p53‐wild‐type GBM, the prodrug may activate this positive feedback loop and exert potent tumor‐killing effects. Nevertheless, this study still has limitations. All mechanistic results are derived from p53‐wild‐type cell lines, and we have not yet validated the prodrug performance in patient‐derived GBM cells carrying different p53 genotypes. GBM exhibits prominent interpatient heterogeneity, and p53‐mutant tumor cells may fail to fully activate the AMPK‐p53 feedback axis and may therefore display weaker therapeutic sensitivity. Even so, cuproptosis‐inducing prodrugs may still reverse TMZ resistance independently of p53 through mitochondrial dysfunction, redox dyshomeostasis, ATP depletion, and glycolysis suppression, which may form alternative feedback loops that bypass defective p53 signaling. In future work, we will investigate this mechanism in p53‐mutant tumor models and verify the therapeutic efficacy of our cuproptosis‐inducing prodrugs using patient‐derived primary cells.

## Author Contributions

**Wenjie Feng**: Conceptualization, Investigation, Data curation, Funding acquisition, Visualization, Writing – original draft, Writing – review & editing; **Rui Yang**: Data curation, Formal analysis, Investigation, Visualization; **Chang Weng**: Investigation, Data curation; **Chen Li**: Investigation, Methodology, Visualization; **Wanrui Shi**: Methodology, Visualization; **Yang Chen**: Investigation, Methodology; **Yang Yang**: Investigation, Visualization; **Yi Liu**: Data curation, Funding acquisition, Supervision, Writing – review & editing; **Pengfei Ge**: Funding acquisition, Methodology, Supervision, Writing – review & editing; **Hao Zhang**: Conceptualization, Funding acquisition, Supervision, Project administration, Writing – review & editing.

## Ethics Approvals

All animal studies were performed in strict adherence to the Laboratory Animal Welfare Guidelines established by the First Hospital of Jilin University, with protocols reviewed and approved by the Institutional Animal Ethics Committee (Ethical Approval Number: 20240448).

## Conflicts of Interest

The authors declare no conflicts of interest.

## Supporting information




**Supporting File**: advs76677‐sup‐0001‐SuppMat.pdf.

## Data Availability

The data that support the findings of this study are available from the corresponding author upon reasonable request.

## References

[1] M. Weller , P. Y. Wen , S. M. Chang , et al., “Glioma,” Nature Reviews Disease Primers 10 (2024): 33, 10.1038/s41572-024-00516-y. 38724526

[2] D. N. Louis , A. Perry , P. Wesseling , et al., “The 2021 WHO Classification Of Tumors Of The Central Nervous System: A Summary,” Neuro‐Oncology 23 (2021): 1231–1251, 10.1093/neuonc/noab106. 34185076 PMC8328013

[3] M. J. van den Bent , M. Geurts , P. J. French , et al., “Primary Brain Tumours In Adults,” Lancet 402 (2023): 1564. 37738997 10.1016/S0140-6736(23)01054-1

[4] A. C. Tan , D. M. Ashley , G. Y. López , M. Malinzak , H. S. Friedman , and M. Khasraw , “Management of Glioblastoma: State of the Art And Future Directions,” CA: A Cancer Journal for Clinicians 70 (2020): 299. 32478924 10.3322/caac.21613

[5] R. Stupp , S. Taillibert , A. Kanner , et al., “Effect of Tumor‐Treating Fields Plus Maintenance Temozolomide vs Maintenance Temozolomide Alone on Survival in Patients With Glioblastoma,” JAMA 318 (2017): 2306, 10.1001/jama.2017.18718. 29260225 PMC5820703

[6] H. L. Shao , J. Chung , K. Lee , et al., “Chip‐Based Analysis of Exosomal mRNA Mediating Drug Resistance In Glioblastoma,” Nature Communications 6 (2015): 6999, 10.1038/ncomms7999. PMC4430127 25959588

[7] A.‐M. Barciszewska , D. Gurda , P. Glodowicz , S. Nowak , and M. Z. Naskręt‐Barciszewska , “A New Epigenetic Mechanism Of Temozolomide Action In Glioma Cells,” PLoS ONE 10 (2015): 0136669, 10.1371/journal.pone.0136669. PMC4550362 26309255

[8] Z. Li , S. Jiang , J. Wang , et al., “Peptide‐Drug Conjugates Repolarize Glioblastoma‐Associated Macrophages To Resensitize Chemo‐Immunotherapy Of Glioblastoma,” Science Advances 11 (2025): adr8841, 10.1126/sciadv.adr8841. PMC11740939 39823328

[9] X. Jin , S. Kang , S. Tanaka , and S. Park , “Monitoring The Glutathione Redox Reaction In Living Human Cells By Combining Metabolic Labeling With Heteronuclear NMR,” Angewandte Chemie International Edition 55 (2016): 7939–7942, 10.1002/anie.201601026. 27174844

[10] Y. Yan , S. Zhou , X. Chen , et al., “Suppression of ITPKB Degradation by Trim25 Confers TMZ Resistance in Glioblastoma Through ROS Homeostasis,” Signal Transduction and Targeted Therapy 9 (2024): 58, 10.1038/s41392-024-01763-x. 38438346 PMC10912509

[11] Z. Lu , X.‐Y. Wang , K.‐Y. He , et al., “CHIP‐Mediated Ubiquitin Degradation of BCAT1 Regulates Glioma Cell Proliferation And Temozolomide Sensitivity,” Cell Death & Disease 15 (2024): 538, 10.1038/s41419-024-06938-6. 39075053 PMC11286746

[12] Y. Zhang , H. Ma , L. Li , et al., “Dual‐Targeted Novel Temozolomide Nanocapsules Encapsulating siPKM2 Inhibit Aerobic Glycolysis to Sensitize Glioblastoma to Chemotherapy,” Advanced Materials 36 (2024): 2400502, 10.1002/adma.202400502. 38651254

[13] A. Wolf , S. Agnihotri , J. Micallef , et al., “Hexokinase 2 is a Key Mediator Of Aerobic Glycolysis And Promotes Tumor Growth In Human Glioblastoma Multiforme,” Journal of Experimental Medicine 208 (2011): 313–326, 10.1084/jem.20101470. 21242296 PMC3039857

[14] J. L. Munoz , N. D. Walker , K. W. Scotto , and P. Rameshwar , “Temozolomide Competes for P‐Glycoprotein And Contributes To Chemoresistance In Glioblastoma Cells,” Cancer Letters 367 (2015): 69–75, 10.1016/j.canlet.2015.07.013. 26208431

[15] T. I. Janjua , Y. X. Cao , A. Ahmed‐Cox , et al., “Efficient Delivery Of Temozolomide Using Ultrasmall Large‐Pore Silica Nanoparticles For Glioblastoma,” Journal of Controlled Release 357 (2023): 161–174, 10.1016/j.jconrel.2023.03.040. 36965857

[16] H. Chen , S. Zhang , Q. Fang , et al., “Biomimetic Nanosonosensitizers Combined With Noninvasive Ultrasound Actuation To Reverse Drug Resistance And Sonodynamic‐Enhanced Chemotherapy Against Orthotopic Glioblastoma,” ACS Nano 17 (2022): 421–436, 10.1021/acsnano.2c08861. 36573683

[17] Y. Wang , Y. Jiang , D. Wei , et al., “Nanoparticle‐Mediated Convection‐Enhanced Delivery of a DNA Intercalator To Gliomas Circumvents Temozolomide Resistance,” Nature Biomedical Engineering 5 (2021): 1048–1058, 10.1038/s41551-021-00728-7. PMC8497438 34045730

[18] Q. Xu , X. Hu , I. Ullah , et al., “Biomimetic Hybrid PROTAC Nanovesicles Block Multiple DNA Repair Pathways To Overcome Temozolomide Resistance Against Orthotopic Glioblastoma,” Advanced Materials 37 (2025): 2504253, 10.1002/adma.202504253. 40347032

[19] Q. Yue , Z. Wang , Y. Shen , et al., “Histone H3K9 Lactylation Confers Temozolomide Resistance In Glioblastoma via LUC7L2‐Mediated MLH1 Intron Retention,” Advanced Sciences 11 (2024): 2309290. 10.1002/advs.202309290 PMC11109612 38477507

[20] W. Dong , A. Fekete , X. Chen , et al., “A designer peptide Against the EAG2–Kvβ2 Potassium Channel Targets The Interaction Of Cancer Cells And Neurons To Treat Glioblastoma,” Nature Cancer 4 (2023): 1418–1436, 10.1038/s43018-023-00626-8. 37697045

[21] P. Tsvetkov , S. Coy , B. Petrova , et al., “Copper Induces Cell Death By Targeting Lipoylated TCA Cycle Proteins,” Science 375 (2022): 1254–1261, 10.1126/science.abf0529. 35298263 PMC9273333

[22] D. Tang , G. Kroemer , and R. Kang , “Targeting Cuproplasia And Cuproptosis In Cancer,” Nature Reviews Clinical Oncology 21 (2024): 370–388, 10.1038/s41571-024-00876-0. 38486054

[23] K. Hadian and B. R. Stockwell , “The Therapeutic Potential Of Targeting Regulated Non‐Apoptotic Cell Death,” Nature Reviews Drug Discovery 22 (2023): 723–742, 10.1038/s41573-023-00749-8. 37550363

[24] J. Y. Yuan and D. Ofengeim , “A Guide To Cell Death Pathways,” Nature Reviews Molecular Cell Biology 25 (2024): 379–395, 10.1038/s41580-023-00689-6. 38110635

[25] S. R. Li , L. L. Bu , and L. L. Cai , “Cuproptosis: Lipoylated TCA Cycle Proteins‐Mediated Novel Cell Death Pathway,” Signal Transduction and Targeted Therapy 7 (2022): 158, 10.1038/s41392-022-01014-x. 35562341 PMC9106713

[26] G. Q. Zhu , Y. L. Xie , J. R. Wang , et al., “Multifunctional Copper‐Phenolic Nanopills Achieve Comprehensive Polyamines Depletion to Provoke Enhanced Pyroptosis and Cuproptosis for Cancer Immunotherapy,” Advanced Materials 36 (2024): 2409066, 10.1002/adma.202409066. 39285820

[27] S. Herzig and R. J. Shaw , “AMPK: Guardian Of Metabolism And Mitochondrial Homeostasis,” Nature Reviews Molecular Cell Biology 19 (2018): 121–135, 10.1038/nrm.2017.95. 28974774 PMC5780224

[28] R. G. Jones , D. R. Plas , S. Kubek , et al., “AMP‐Activated Protein Kinase Induces a p53‐Dependent Metabolic Checkpoint,” Molecular Cell 18 (2005): 283–293, 10.1016/j.molcel.2005.03.027. 15866171

[29] C. Xiong , H. Ling , Q. Hao , and X. Zhou , “Cuproptosis: P53‐Regulated Metabolic Cell Death?,” Cell Death & Differentiation 30 (2023): 876–884, 10.1038/s41418-023-01125-0. 36755067 PMC10070433

[30] Q. Q. Qiao , S. F. Hu , and X. Wang , “The Regulatory Roles And Clinical Significance Of Glycolysis In Tumor,” Cancer Communications 44 (2024): 761–786, 10.1002/cac2.12549. 38851859 PMC11260772

[31] H. Zafar , J. Zhang , F. Raza , et al., “Biomimetic Gold Nanocages Incorporating Copper‐Human Serum Albumin For Tumor Immunotherapy *Via* Cuproptosis‐Lactate Regulation,” Journal of Controlled Release 372 (2024): 446–466, 10.1016/j.jconrel.2024.06.059. 38917953

[32] X. Wu , S. Zhang , M. Feng , et al., “Glutathione Induced In Situ Activation of Dual‐Locked Cuproptosis Nanoamplifier With Glycolysis Metabolism Inhibition to Boost Cancer Immunotherapy,” Advanced Healthcare Materials 14 (2024): 2403380, 10.1002/adhm.202403380. 39686828

[33] Y. Wang , X. Yao , Y. Lu , et al., “A PROTAC‐Based Cuproptosis Sensitizer in Lung Cancer Therapy,” Advanced Materials 37 (2025): 2501435, 10.1002/adma.202501435. 40495637

[34] Y. Hu , H. Chen , B. Chen , et al., “Charge and Size‐Variable Biodegradable Nanocomposites for Self‐Reinforcing CDT, PTT, and Chemotherapy: Augmented Cuproptosis and Ferroptosis Against Tumor Hypoxia via Glycolysis/Redox Dual Disruption,” Advanced Functional Materials 35 (2025): 2503038, 10.1002/adfm.202503038.

[35] Y. Chang , J. Chang , J. Ji , et al., “Novel Thioredoxin Reductase 1 Inhibitor BS1801 Relieves Treatment Resistance And Triggers Endoplasmic Reticulum Stress By Elevating Reactive Oxygen Species In Glioma,” Redox Biology 86 (2025): 103827, 10.1016/j.redox.2025.103827. 40914135 PMC12446627

[36] Y. Bi , H. Li , D. Yi , et al., “β‐Catenin Contributes to Cordycepin‐Induced MGMT Inhibition And Reduction Of Temozolomide Resistance In Glioma Cells By Increasing Intracellular Reactive Oxygen Species,” Cancer Letters 435 (2018): 66–79, 10.1016/j.canlet.2018.07.040. 30081068

[37] Y. Liu , H. Zhao , R. Niu , et al., “Single‐Site Nanozyme with Exposed Unsaturated Cu‐O_2_ Sites For Tumor Therapy By Coordinating Innate Immunity And Vasculature Normalization,” Chem 11 (2025): 102297.

[38] X. Shao , C. Qu , G. Song , et al., “NIR‐II Photothermal Activation of TRPV1 Channels for Intracellular Magnesium Regulation by Porous Pd@Pt Core–Shell Nanostructure to Reverse Tumor Multidrug Resistance,” Advanced Functional Materials 33 (2023): 2306585, 10.1002/adfm.202306585.

[39] J. Dong , Z. Qin , W.‐D. Zhang , et al., “Medicinal Chemistry Strategies to Discover P‐Glycoprotein Inhibitors: An Update,” Drug Resistance Update 49 (2020): 100681. 10.1016/j.drup.2020.100681 32014648

[40] I. V. Martin and S. A. Macneill , “ATP‐Dependent DNA Ligases,” Genome Biology 3 (2002): reviews3005.1, 10.1186/gb-2002-3-4-reviews3005. 11983065 PMC139351

[41] G. C. Bobustuc , C. H. Baker , A. Limaye , et al., “Levetiracetam Enhances p53‐Mediated MGMT Inhibition And Sensitizes Glioblastoma Cells To Temozolomide,” Neuro‐Oncology 12 (2010): 917–927, 10.1093/neuonc/noq044. 20525765 PMC2940696

[42] A. Natsume , D. Ishii , T. Wakabayashi , et al., “IFN‐β Down‐Regulates The Expression of DNA Repair Gene MGMT and Sensitizes Resistant Glioma Cells To Temozolomide,” Cancer Research 65 (2005): 7573–7579, 10.1158/0008-5472.CAN-05-0036. 16140920

[43] G. Thomas , E. Uta , and K. Bernd , “p53 is Involved in Regulation of the DNA Repair Gene O^6^‐methylguanine‐DNA Methyltransferase (MGMT) by DNA Damaging Agents,” Oncogene 17 (1998): 845. 9780001 10.1038/sj.onc.1202000

[44] M. Guan , K. Cheng , X.‐T. Xie , et al., “Regulating Copper Homeostasis Of Tumor Cells To Promote Cuproptosis For Enhancing Breast Cancer Immunotherapy,” Nature Communications 15 (2024): 10060, 10.1038/s41467-024-54469-7. PMC11579316 39567558

[45] M. Hamza , S. Wang , H. Wu , et al., “Targeting Copper Homeostasis: Akkermansia‐Derived OMVs Co‐Deliver Atox1 siRNA and Elesclomol For Cancer Therapy,” Acta Pharmaceutica Sinica B 15 (2025): 2640–2654, 10.1016/j.apsb.2025.03.014. 40487636 PMC12145039

[46] R. P. Planalp , S. Tsymbal , A. Refeld , V. Zatsepin , and O. Kuchur , “The p53 Protein is a Suppressor of Atox1 Copper Chaperon In Tumor Cells Under Genotoxic Effects,” PLoS ONE 18 (2023): 0295944. 10.1371/journal.pone.0295944 PMC10735018 38127999

[47] X. Z. Xu , H. Zhou , R. X. Hong , et al., “A Self‐Accelerating ‘Copper Bomb’ Strategy Activated Innate And Adaptive Immune Response Against Triple‐Negative Breast Cancer,” Bioactive Materials 49 (2025): 193. 40130080 10.1016/j.bioactmat.2025.02.019 PMC11931225

[48] S. Wu , R. Cao , B. Tao , et al., “Pyruvate Facilitates FACT‐Mediated γ H2AX Loading to Chromatin and Promotes the Radiation Resistance of Glioblastoma,” Advanced Science 9 (2022): 2104055, 10.1002/advs.202104055. 35048565 PMC8922107

[49] J. Chen , Z. Huang , Y. Chen , et al., “Lactate and Lactylation in Cancer,” Signal Transduction and Targeted Therapy 10 (2025): 38, 10.1038/s41392-024-02082-x. 39934144 PMC11814237

[50] Y. He , Y. Nie , G. Cheng , L. Xie , Y. Shen , and Z. Gu , “Viral Mimicking Ternary Polyplexes: A Reduction‐Controlled Hierarchical Unpacking Vector for Gene Delivery,” Advanced Materials 26 (2014): 1534–1540, 10.1002/adma.201304592. 24757715

[51] F. Liu , L. Lin , Y. Zhang , et al., “A Tumor‐Microenvironment‐Activated Nanozyme‐Mediated Theranostic Nanoreactor for Imaging‐Guided Combined Tumor Therapy,” Advanced Materials 31 (2019): 1902885, 10.1002/adma.201902885. 31423690

[52] H. Yan , P. C. Xu , H. Ma , et al., “Enzyme‐Triggered Transcytosis Of Drug Carrier System For Deep Penetration Into Hepatoma Tumors,” Biomaterials 301 (2023): 122213, 10.1016/j.biomaterials.2023.122213. 37385137

[53] C. Zhang , T. Huang , and L. Li , “Targeting Cuproptosis For Cancer Therapy: Mechanistic Insights And Clinical Perspectives,” Journal of Hematology & Oncology 17 (2024): 68, 10.1186/s13045-024-01589-8. 39152464 PMC11328505

[54] X. Zhan , H. Xiao , X. Zhang , et al., “A Metallic Metabolic Nano‐Regulator Reprograms the PKM2/HIF‐1α/DLAT Axis To Amplify Tumor‐Specific Cuproptosis,” Biomaterials 325 (2026): 123626, 10.1016/j.biomaterials.2025.123626. 40815897

[55] L. Yang , Z. Y. Zhao , B. S. Tian , et al., “A Singular Plasmonic‐Thermoelectric Hollow Nanostructure Inducing Apoptosis And Cuproptosis For Catalytic Cancer Therapy,” Nature Communications 15 (2024): 7499, 10.1038/s41467-024-51772-1. PMC11362521 39209877

[56] X. Y. Ma , Y. M. Liu , S. K. Li , K. Ogino , R. R. Xing , and X. H. Yan , “Multicomponent Coassembled Nanodrugs Based On Ovalbumin, Pheophorbide a and Zn^2+^ for In Vitro Photodynamic Therapy,” Supramolecular Materials 1 (2022): 100010.

[57] P. S. Steeg , “The Blood–Tumour Barrier In Cancer Biology And Therapy,” Nature Reviews Clinical Oncology 18 (2021): 696–714, 10.1038/s41571-021-00529-6. 34253912

