# Supplementary material for: Reversing Temozolomide Resistance in Glioblastoma Based on Cuproptosis‐Mediated Positive Feedback Loop
Source: Adv Sci (Weinh). 2026 Jul 20;13(57):e76677. doi: 10.1002/advs.76677 (PMC13383701; doi:10.1002/advs.76677)

Supporting Information

Reversing Temozolomide Resistance in Glioblastoma Based on Cuproptosis-Mediated Positive Feedback Loop

Wenjie Feng, Rui Yang, Chang Weng, Chen Li, Wanrui Shi, Yang Chen, Yang Yang, Yi Liu, Pengfei Ge,* and Hao Zhang**

Experimental Section

Materials. Copper(II) nitrate trihydrate ($Cu(NO_3)_2·3H_2O$), temozolomide (TMZ), metformin (MET), disodium ethylenediaminetetraacetate (EDTA), dimethyl sulfoxide (DMSO), urea, sodium chloride (NaCl), glutathione (GSH), ortho-phthaldialdehyde (OPA), neocuproine, methylene blue (MB), chelator tetrathiomolybdate (TTM), dorsomorphin, IR780, and D-luciferin potassium salt were purchased from Aladdin Biochemical Technology Co., Ltd. Terephthalic acid (TPA) was bought from Alfa Aesar. DMEM medium was sourced from Cytia. Fetal bovine serum (FBS) was purchased from Clark Bioscience. Triton X-100 and ATP7A polyclonal antibody were purchased from Beijing Solarbio Science & Technology Co., Ltd. CCK-8 was bought from Yeasen Biotechnology (Shanghai) Co., Ltd. Rotenone and UK 5099 were purchased from Selleck Chemicals. DLAT polyclonal antibody, FDX1 polyclonal antibody, LIAS polyclonal antibody, AMPKα polyclonal antibody, antioxidant 1 (ATOX1) polyclonal antibody, Cyclin B1 polyclonal antibody, p21 polyclonal antibody, P glycoprotein (P-gp) polyclonal antibody, DNA ligase III (LIG3) polyclonal antibody, O^6-methylguanine-DNA methyltransferase (MGMT) polyclonal antibody, alpha tubulin polyclonal antibody, and hexokinase-2 (HK2) polyclonal antibody were purchased from Proteintech Group, Inc. Phospho-AMPKα (Thr172) (40H9) rabbit monoclonal antibody (#2535), phospho-CDK1 (Tyr15) (10A11) rabbit monoclonal antibody, and phospho-histone H2AX (Ser139) (20E3) rabbit monoclonal antibody (#9718) were purchased from Cell Signaling Technology. The lyso-tracker red, ATP assay kit, and JC-1 mitochondrial membrane potential assay kit were provided by Beyotime Biotechnology. Glycolysis stress test kit (103020-100), XFe24 FluxPak, Seahorse XF DMEM medium, and glutamine solution were purchased from Agilent. Lactic acid assay kit, and reduced GSH assay kit were bought from Nanjing Jiancheng Bioengineering Institute. 4% paraformaldehyde, and SuperRed/GelRed were purchased from Biosharp Life Sciences.

Characterization. Transmission electron microscopy (TEM) imaging was conducted on a JEOL JEM-2100F system. UV-vis absorption spectra were obtained using a Shimadzu 2600 spectrophotometer. Dynamic light scattering (DLS) measurements were performed with a Malvern Zetasizer Nano ZS. FTIR spectra were acquired on a Bruker IFS80V spectrometer. X-ray photoelectron spectroscopy (XPS) spectra were measured by using an X-ray photoelectron spectrometer (AXIS SUPRA+). Copper quantification was carried out by inductively coupled plasma-optical emission spectrometry (ICP-OES) with an Agilent 725, while inductively coupled plasma-mass spectrometry (ICP-MS) was executed on an iCAP Qc. Glycolysis stress tests were assessed using an Agilent Seahorse XF24 Analyzer. Multi-wavelength absorbance detection was enabled by an Infinite M Nano microplate reader (Tecan Austria GmbH).

Confocal laser scanning microscopy (CLSM) was conducted with a Nikon AX system.

Preparation of Cu-TMZ prodrugs. Cu-TMZ prodrugs were prepared through the self-assembly between Cu^{2+} and TMZ. 3 mM $Cu(NO_3)_2 \cdot 3H_2O$, 1 mM TMZ, and 2 mM NaOH were dissolved in ultrapure water. After continuous stirring at 40 °C for 4 h, the Cu-TMZ prodrugs were collected *via* centrifugation, washed, and re-suspended in ultrapure water for subsequent applications.

Preparation of Cu-TMZ/MET prodrugs. 0.6 mL of MET (20 mg/mL) and 2 mL of Cu-TMZ prodrugs (3 mg/mL) were added in 28 mL ultrapure water. After continuous stirring at 850 rpm/min for 2 h, the Cu-TMZ/MET prodrugs were collected *via* centrifugation, washed, and re-suspended in ultrapure water for subsequent applications.

Assembly driving forces. A 100 μL Cu-TMZ/MET prodrug solution was separately combined with 1 mL of solutions containing DMSO, urea, NaCl, Triton X-100, or EDTA. The mixtures were incubated for 10 h under static conditions. Subsequently, the colloidal stability was visually assessed, followed by characterization using UV-vis absorption spectroscopy, DLS, and TEM.

GSH depletion ability analysis. To detect the GSH depletion ability of Cu-TMZ/MET prodrugs, OPA was employed as a fluorescent indicator. Prodrugs at different concentrations (0, 10, 20, 30, 40, 50, 60, 70, and 80 μg/mL) were incubated in GSH solution. GSH depletion was detected according to the change of photoluminescence (PL) intensity by using the fluorescence spectrometer (λ_{ex} = 350 nm; λ_{em} = 420 nm).

Analyzing the release behavior of Cu^+ and TMZ. To investigate the release behavior of TMZ, prodrugs were incubated in dialysis bags with or without 10 mM GSH under continuous agitation. At predetermined time points, 3 mL of the supernatant was sampled and replaced with an equal volume of fresh medium. The release of TMZ was quantified by measuring the absorbance of the supernatant at 254 nm using UV-vis absorption spectroscopy, with concentrations calculated against a pre-established standard curve. For Cu^+, the release behavior was monitored indirectly using neocuproine as a probe, with changes analyzed by UV-vis absorption spectroscopy.

Generation of ·OH analysis. 60 μg/mL prodrugs were pre-incubated with 2 mM GSH in solution at 37 °C for 2 h. ·OH generation was assessed using two independent methods: (i) TPA fluorescence assay. The pre-treated mixture was supplemented with 100 μM H_2O_2 and 20 μM TPA. ·OH production was quantified by monitoring the PL emission spectrum (λ_{ex} = 310 nm) of 2-hydroxyterephthalic acid, the oxidative product of TPA. (ii) MB decay assay. The reaction system was initiated by adding 100 μM H_2O_2 and 20 μL 1% MB to the mixture. ·OH-mediated

MB degradation was tracked *via* UV-vis absorption spectroscopy by measuring the decay of the characteristic MB peak at 664 nm.

Cell culture. U87 cells, A172 cells, L929 cells, and bEnd.3 cells were cultured in complete DMEM (10% FBS and 1% penicillin-streptomycin) at 37 °C with 5% CO_2. TMZ-resistant (U87/TR) cells were established by treating parental U87 cells with increasing dosages of TMZ until they were resistant to TMZ as previously described[1].

Cell cytotoxicity analysis. Cytotoxicity assay was conducted using CCK-8 method. Briefly, U87 cells, U87/TR cells, A172 cells, bEnd.3 cells, or L929 cells were seeded in 96-well plates and adhered for 24 h. And then, they were incubated with different concentrations of Cu-TMZ or Cu-TMZ/MET prodrugs for another 24 h. Following the incubation, cell viability was analyzed using the CCK-8 assay *via* microplate reader. To evaluate the cytotoxicity of prodrugs on U87 cells or U87/TR cells in the presence of cuproptosis inhibitors or copper ion chelator, rotenone, UK 5099 or TTM was pre-introduced to cells 6 h prior to treatment. To evaluate the cytotoxicity of Cu-TMZ prodrugs on U87 cells or U87/TR cells in the presence of AMPK activator, MET was added for co-incubation. As for the cytotoxicity of Cu-TMZ/MET prodrugs on U87/TR cells in the presence of AMPK inhibitor, dorsomorphin was added for co-incubation.

Cellular uptake and localization assay. ^{FITC}Cu-TMZ/MET prodrugs were prepared first. FITC and Cu-TMZ/MET prodrugs were mixed in an aqueous solution at a mass ratio of 1:4, stirred for 24 h in dark, and then the FITC-labeled prodrugs were collected *via* centrifugation, washed, and re-suspended in ultrapure water for subsequent applications. The successful establishment of ^{FITC}Cu-TMZ/MET prodrugs was confirmed by analyzing fluorescence spectra and UV-vis absorption spectra. U87/TR cells were seeded in confocal dishes and adhered for 24 h. And then, they were incubated with 0 μg/mL or 60 μg/mL ^{FITC}Cu-TMZ/MET prodrugs for 24 h. Subsequently, the cells were washed three times, and incubated with 75 nM lyso-tracker red working solution and 1X Hoechst 33342 at 37 °C for 15 min. Finally, they were washed and studied for CLSM (Hoechst 33342: λex = 405 nm, λem = 429-474 nm; FITC: λex = 488 nm, λem = 499-530 nm; lyso-tracker red: λex = 561 nm, λem = 475-600 nm).

Live and dead cell analysis. U87/TR cells were seeded in confocal dishes and adhered for 24 h. And then, they were incubated with Cu^{2+}, TMZ, MET, Cu-TMZ prodrugs, or Cu-TMZ/MET prodrugs at an equivalent dosage of 60 μg/mL Cu-TMZ/MET for another 24 h. 60 μg/mL Cu-TMZ/MET contains 21.3 μg/mL, 0.11 mM TMZ. Subsequently, a calcein-AM/PI working solution was added into the dishes, and the U87/TR cells were incubated with working solution for 30 min at 37 °C. Finally, the cells were washed and studied on CLSM (calcein AM: λex = 488 nm, λem = 501-551 nm; PI: λex = 561 nm, λem = 587-723 nm).

Intracellular expressions of DLAT, ATOX1, and γ-H2AX analyzed by immunofluorescent cell staining. U87 or U87/TR cells were seeded in confocal dishes and adhered for 24 h. And then, they were incubated with Cu^{2+}, TMZ, MET, Cu-TMZ prodrugs, or Cu-TMZ/MET prodrugs at an equivalent dosage of 60 μg/mL Cu-TMZ/MET for another 24 h. Subsequently, the cells were washed and fixed with 4% paraformaldehyde. After that, they were permeabilized by using permeabilization buffer. Following another wash, the U87 or U87/TR cells were blocked with a blocking buffer. Then, they were treated with DLAT, ATOX1 polyclonal antibody or phospho-histone H2AX monoclonal antibody overnight at 4 °C. After washing, the U87 or U87/TR cells were incubated with an IgG H&L/FITC-conjugated secondary antibody for 1 h in dark. The U87 or U87/TR cells were washed again and then stained with 1X Hoechst 33342. Finally, the cells were washed and studied on CLSM (Hoechst 33342: λex = 405 nm, λem = 429-474 nm; FITC labeled DLAT: λex = 488 nm, λem = 505-544 nm; FITC labeled ATOX1: λex = 488 nm, λem = 499-530 nm; FITC labeled γ-H2AX: λex = 488 nm, λem = 499-530 nm).

Intracellular expressions of LIAS, FDX1, p-AMPK, AMPK, p-p53, p53, HK2, ATP7A, γ-H2AX, Cyclin B1, p21, p-CDK1, LIG3, MGMT, and P-gp analyzed by western blotting. U87, U87/TR, or T98G cells were seeded in 6-well plates and adhered for 24 h. And then, they were incubated with Cu^{2+}, TMZ, MET, Cu-TMZ prodrugs, or Cu-TMZ/MET prodrugs at an equivalent dosage of 60 μg/mL Cu-TMZ/MET for another 24 h. Subsequently, cells were harvested and lysed. The protein supernatant was collected by centrifugation. Subsequently, proteins were separated using gel electrophoresis and then they were transferred onto PVDF membranes. The PVDF membranes were sectioned according to the molecular weight indications provided by the colored protein standard. Subsequently, the membranes were blocked and incubated with primary antibodies against β-actin, LIAS, FDX1, p-AMPK, AMPK, p-p53, p53, HK2, ATP7A, γ-H2AX, Cyclin B1, p21, p-CDK1, LIG3, MGMT, P-gp, GAPDH, and alpha tubulin overnight at 4 °C. Finally, secondary antibody was used, and protein bands were visualized using an ECL kit. Bands were normalized to β-actin on the same membrane as the internal reference. In cases where target proteins overlapped with β-actin in molecular weight, alpha tubulin or GAPDH was used instead.

Intracellular copper content analysis. U87/TR cells were seeded in 15 cm dishes and adhered for 24 h. And then, they were incubated with Cu^{2+}, TMZ, MET, Cu-TMZ prodrugs, or Cu-TMZ/MET prodrugs at an equivalent dosage of 60 μg/mL Cu-TMZ/MET for another 24 h. Following three PBS washes, cells were subjected to acid digestion. The digested solution was membrane-filtered, and intracellular copper content was quantified using ICP-MS.

Intracellular JC-1 mitochondrial membrane potential analysis. U87/TR cells were seeded in confocal dishes and adhered for 24 h. And then, they were incubated with Cu^{2+}, TMZ, MET, Cu-TMZ prodrugs, or Cu-TMZ/MET prodrugs at an equivalent dosage of 60 μg/mL Cu-TMZ/MET for another 24 h. Subsequently, JC-1 working solution was added into the dishes, and the U87/TR cells were incubated with working solution at 37 °C for 20 min. And then, the cells were washed again and then stained with 1X Hoechst 33342. Finally, the cells were washed and studied on CLSM (Hoechst 33342: λex = 405 nm, λem = 429-474 nm; JC-1 Monomers: λex = 488 nm, λem = 499-551 nm; JC-1 Aggregates: λex = 561 nm, λem = 571-625 nm).

Intracellular ATP level analysis. U87/TR cells were seeded in confocal dishes and adhered for 24 h. And then, they were incubated with Cu^{2+}, TMZ, MET, Cu-TMZ prodrugs, or Cu-TMZ/MET prodrugs at an equivalent dosage of 60 μg/mL Cu-TMZ/MET for another 24 h. Subsequently, U87/TR cells were harvested and lysed. And then, the supernatant of each group was collected by centrifugation and the ATP level was studied according to the ATP assay kit.

Intracellular lactic acid content analysis. U87/TR cells were seeded in 6-well plates and adhered for 24 h. And then, they were incubated with Cu^{2+}, TMZ, MET, Cu-TMZ prodrugs, or Cu-TMZ/MET prodrugs at an equivalent dosage of 60 μg/mL Cu-TMZ/MET for another 24 h. Subsequently, U87/TR cells were harvested and lysed. And then, the supernatant of each group was collected by centrifugation and the GSH content was studied according to the lactic acid assay kit.

Extracellular acidification rate analysis. U87/TR cells were seeded in Seahorse XF24 cell culture microplate and adhered for 24 h. And then, they were incubated with PBS, Cu-TMZ prodrugs, or Cu-TMZ/MET prodrugs at an equivalent dosage of 60 μg/mL Cu-TMZ/MET for another 24 h. Subsequently, extracellular acidification rate (ECAR) was measured using the Seahorse XFe24 analyzer.

Intracellular ROS generation analysis. U87/TR cells were seeded in confocal dishes and adhered for 24 h. And then, they were incubated with Cu^{2+}, TMZ, MET, Cu-TMZ prodrugs, or Cu-TMZ/MET prodrugs at an equivalent dosage of 60 μg/mL Cu-TMZ/MET for another 24 h. Subsequently, DCFH-DA was added into the dishes, and the U87/TR cells were incubated with DCFH-DA at 37 °C for 20 min. And then, the cells were washed again and then stained with 1X Hoechst 33342. Finally, the cells were washed and studied on CLSM (Hoechst 33342: λex = 405 nm, λem = 429-474 nm; DCFH-DA: λex = 488 nm, λem = 510-552 nm).

Intracellular GSH content analysis. U87/TR cells were seeded in 6-well plates and adhered for 24 h. And then, they were incubated with Cu^{2+}, TMZ, MET, Cu-TMZ prodrugs, or Cu-TMZ/MET prodrugs at an equivalent dosage of 60 μg/mL Cu-TMZ/MET for another 24 h.

Subsequently, U87/TR cells were harvested and lysed. And then, the supernatant of each group was collected by centrifugation and the GSH content was studied according to the reduced GSH assay kit.

Neutral comet assay. U87 or U87/TR cells were seeded in 6-well plates and adhered for 24 h. And then, they were incubated with TMZ, Cu-TMZ prodrugs, or Cu-TMZ/MET prodrugs at an equivalent dosage of 60 μg/mL Cu-TMZ/MET for another 24 h. Subsequently, harvested U87 or U87/TR cells were suspended in 1 mL low-melting-point agarose (LMPA), and 80 μL aliquots were layered onto comet slides pre-coated with 1% regular agarose. After coverslip placement and gelling at 4 °C for 10 min, coverslips were removed, and slides were sealed with an 80 μL LMPA overlay. Slides were lysed (4 °C, 1 h, dark) in buffer containing 2.5 M NaCl, 100 mM Na_2EDTA, 10 mM Tris, 10% DMSO, and 1% Triton X-100, rinsed in TBE buffer (0.445 M Tris-borate, 0.01 M EDTA), and electrophoresed (25 V, 20 min) in TBE. Post-electrophoresis washes included 0.9% NaCl (2 min) and neutralization with 0.4 M Tris (pH 7.5). DNA was stained with SuperRed/GelRed for 20 min and analyzed *via* fluorescence microscopy. For the comet assay quantification, tail DNA percentage was determined using ImageJ and OpenComet 1.3.1 software. Three independent experiments were performed, with approximately 50 cells evaluated per experiment.

Cell cycle distribution analysis. U87/TR cells were seeded in 6-well plates and adhered for 24 h. And then, they were incubated with PBS, Cu-TMZ prodrugs, or Cu-TMZ/MET prodrugs at an equivalent dosage of 60 μg/mL Cu-TMZ/MET for another 24 h. After washing with PBS, the cells were harvested. Following fixation and PI staining, cell cycle distribution was detected by flow cytometry.

Transcriptome RNA sequencing (RNA-Seq) analysis. U87/TR cells were seeded in 6-well plates and adhered for 24 h. And then, they were incubated with PBS, Cu-TMZ prodrugs, or Cu-TMZ/MET prodrugs at an equivalent dosage of 60 μg/mL Cu-TMZ/MET for another 24 h. After washing with PBS, the cells were harvested, and the total RNA was extracted using TRIzol. The Kyoto Encyclopedia of Genes and Genomes (KEGG) pathway analysis, Gene Ontology (GO) analysis and Reactome analysis were conducted to examine the potential biological roles of differentially expressed genes. Gene set enrichment analysis (GSEA), heat maps and protein-protein interaction (PPI) analysis were used to analysis differentially expressed genes.

Heat map clustering was performed using the pheatmap package in R. The heat map was constructed based on the union of differentially expressed genes from all comparative groups, using FPKM values of these genes across all samples. Briefly, pairwise distances between genes

were calculated based on their relative expression levels represented as log_2(ratios), and hierarchical clustering was performed by iteratively computing relative distances and grouping genes into distinct subclusters according to their expression similarity. Genes with similar expression patterns were thus grouped together. Color intensity in each grid of the heat map represents row z-score normalized values rather than raw gene expression levels. All expression data were normalized and centered as follows: expression values were first transformed as log_2(FPKM + 1), followed by z-score normalization.

Animals. Female BALB/c Nude mice (4 weeks) were purchased from Beijing Vital River Laboratory Animal Technology. All animal studies were performed in strict adherence to the Laboratory Animal Welfare Guidelines established by the First Hospital of Jilin University, with protocols reviewed and approved by the Institutional Animal Ethics Committee (Ethical Approval Number: 20240448). All animal experiments were performed in a randomized and blinded manner. Animals were randomly assigned to different groups.

***In vivo* antitumor efficacy on subcutaneous GBM models.** U87 and U87/TR GBM models were established on BALB/c Nude mice. 4×10^6 U87 or U87/TR cells were inoculated in the right axilla of mice. At 12 days post-inoculation of U87/TR cells, mice were randomly divided into six groups (n = 6): (I) Control, (II) TMZ, (III) Cu^{2+}+TMZ+MET, (IV) Cu-TMZ, (V) Cu-TMZ/MET, and (VI) Cu-TMZ/MET+TTM. Mice with U87 GBM models were randomly divided into two groups: (I) Control, and (II) TMZ. Cu-TMZ/MET prodrugs (10 mg/kg, i.v.) were administered to Group V and Group VI in U87/TR bearing models, while other groups received individual components at doses corresponding to their respective proportions in 10 mg/kg of Cu-TMZ/MET prodrugs. TTM (0.16 mM, 50 μL) was injected into tumor issues. The length and width of tumor and the body weight of mice were recorded every two days. After 14 days, the tumor and major organs including heart, lungs, liver, spleen, and kidneys were collected in each group for hematoxylin and eosin (H&E) staining. Blood was used to study liver and kidney function indexes. To comply with ethical standards for tumor burden limits, experimental animals were sacrificed when the measured tumor volume exceeded size of 1500 mm^3.

Orthotopic GBM models. U87-Luc orthotopic GBM models were constructed in female BALB/c Nude mice. A total of 1×10^5 U87-Luc cells suspended in 5 μL PBS were injected into the striatum of mice (2 mm posterior to coronal suture and 1 mm lateral to the sagittal suture, and to a depth of 2.6 mm). After inoculation with U87-Luc cells, the successful establishment of the orthotopic GBM models was confirmed by bioluminescent imaging using an IVIS imaging system.

Preparation of ^{IR780}Cu-TMZ/MET prodrugs. IR780 and Cu-TMZ/MET prodrugs were mixed in an aqueous solution at a mass ratio of 1:4, stirred for 24 h in dark. The ^{IR780}Cu-TMZ/MET prodrugs were collected *via* centrifugation, washed, and re-suspended in ultrapure water for subsequent applications. The successful establishment of ^{IR780}Cu-TMZ/MET prodrugs was confirmed by analyzing UV-vis absorption spectra, fluorescence spectra, and NIR-II fluorescence imaging.

***In vivo* pharmacokinetics analysis.** U87-Luc orthotopic GBM models were constructed as described above. ^{IR780}Cu-TMZ/MET prodrugs were intravenously injected. A two-dimensional InGaAs array (Raptor Photonics) was used to collect NIR fluorescence signals. The mice were anesthetized using isoflurane and imaged (λex = 808 nm, λem > 1000 nm) at 0, 1, 2, 4, 6, 8, 10, and 24 h post-injection. The brain and major organs including heart, lungs, liver, kidneys, and spleen of mice were collected and imaged at 24 h. Furthermore, blood samples from the orbital venous plexus in mice were collected into anticoagulant tubes. Blood circulation profiles were drawn by recording the IR780 fluorescence intensity of the blood samples at 0, 0.5, 1, 1.5, 2.5, 4, and 5 h post-injection.

***In vivo* antitumor efficacy on orthotopic GBM models.** U87-Luc orthotopic GBM models were constructed as described above. At 10 days post-inoculation of U87-Luc cells, mice were randomly divided into six groups (n = 4): (I) Control, (II) TMZ, (III) Cu^{2+}+TMZ+MET, (IV) Cu-TMZ, (V) Cu-TMZ/MET, and (VI) Cu-TMZ/MET+TTM. Cu-TMZ/MET prodrugs (10 mg/kg, i.v.) were administered to Group V and Group VI in orthotopic GBM models, while other groups received individual components at doses corresponding to their respective proportions in 10 mg/kg of Cu-TMZ/MET prodrugs. TTM (0.16 mM, 50 μL) was injected in Group VI. The growth of GBM tumors was monitored using IVIS for bioluminescence imaging. The IVIS Lumina XR system (Caliper Life Sciences) was used to capture bioluminescence signals. Bioluminescence imaging parameters: D-luciferin potassium salt (150 mg/kg) was intraperitoneally injected into mice, followed by a 10 min biodistribution period before signal capture. Bioluminescence images were acquired with an exposure time of 60 s, f-stop = 1, binning = 4, open emission filter, blocked excitation filter, and a field of view of 7.5. The body weight of mice was recorded. Bioluminescence imaging instrument: At the termination of the experiment, the brain tissues were fixed in 4% paraformaldehyde for H&E staining, and whole-slide scanning was conducted to compare the tumor area among different groups.

Hemolysis assay. Cu-TMZ and Cu-TMZ/MET prodrugs at various concentrations (0, 50, 100, 200, and 500 μg/mL) were incubated with 1% red blood cell suspensions in PBS at 37 °C for 1 h. Ultrapure water was used as the positive control. After incubation, the supernatant was

collected, and the absorbance at 540 nm was measured to calculate the hemolysis rate.

The immunofluorescence image quantification. Images were analyzed in a blinded manner using ImageJ. A consistent intensity threshold was applied to all images within the same experiment. At least three random fields of view were captured per group, and approximately 50 cells were quantified per condition. Relative fluorescence intensity was calculated with the control group set as 1.

Statistical analysis. Raw functional data were normalized to corresponding control groups for batch correction. No extreme outliers were observed across all datasets, and all measured values were retained for subsequent statistical analysis. Quantitative results represent mean values ± standard deviation from independent experiments (n indicated in figure legends). All statistical tests were two-sided, and the predefined significance level was set as $\alpha = 0.05$. Statistical analyses employed one-way or two-way analysis of variance (ANOVA). When significant variation was detected ($p < 0.05$), Tukey's or Bonferroni's post-hoc multiple comparison test was applied to determine intergroup differences (significance thresholds: $^*p < 0.05$, $^{**}p < 0.01$, and $^{***}p < 0.001$). All statistical calculations and plotting were conducted using OriginPro.

Data availability. The main data to support the results of this study are presented in the Manuscript and the Supporting Information. Transcriptome RNA-seq data generated in this study can be found in the Gene Expression Omnibus (GEO). GEO submission number is GSE311150.

REFERENCE

[1] Z. C. Wang, C. Li, Z. Zhang, S. Lu, Y. M. Liu, P. Qi, X. Chen, Y. B. Wang, W. J. Feng, C. L. Pan, Q. X. Wang, Z. L. Ji, Y. Yu, M. H. Piao, G. F. Chi, P. F. Ge, Targeting PPARα activation sensitizes glioblastoma cells to temozolomide and reverses acquired resistance by inhibiting H3K18 lactylation. *Acta Pharmacol. Sin.* **2025**, *46*, 3071.

Supplementary Figures

Figure S1. Adjustment of the molar feed ratio of $Cu(NO_3)_2$ and TMZ in the preparation of Cu-TMZ prodrugs. TEM images (a) and size distribution (b) of Cu-TMZ prodrugs prepared with a fixed TMZ concentration of 1.0 mM, NaOH concentration of 2.0 mM, temperature of 25 °C, reaction time of 24 h, and different $Cu(NO_3)_2$ concentrations of 2.0 mM, 3.0 mM, and 4.0 mM.

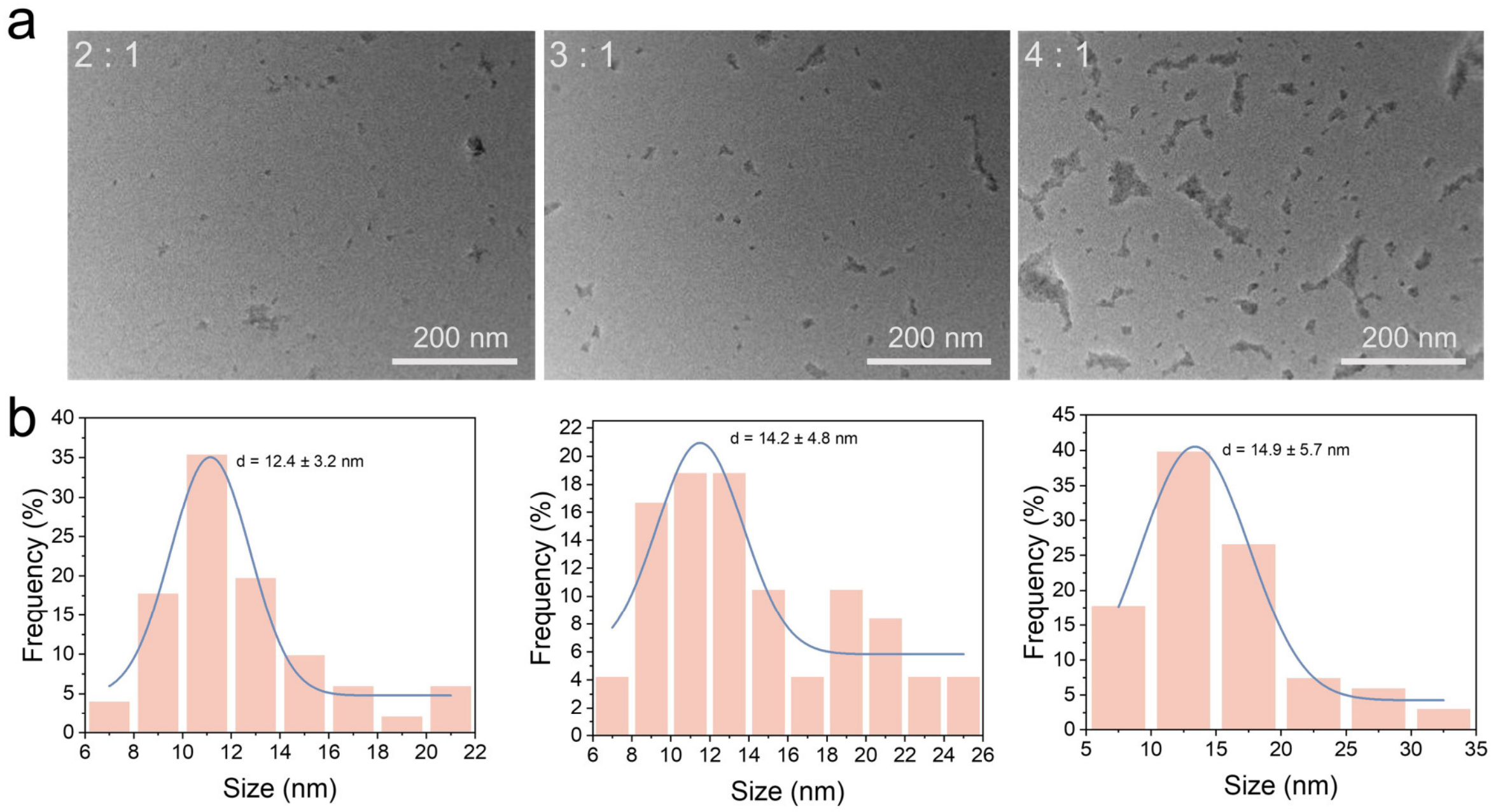

Figure S2. Adjustment of the concentration of NaOH in the preparation of Cu-TMZ prodrugs. TEM images (a) and size distribution (b) of Cu-TMZ prodrugs prepared with a fixed $Cu(NO_3)_2$-to-TMZ molar feed ratio of 3:1, temperature of 25 °C, reaction time of 24 h, and different NaOH concentrations of 0.5 mM, 1.0 mM, and 3.0 mM. Please note that the condition of 2.0 mM NaOH is identical to the condition of $Cu(NO_3)_2$-to-TMZ feed ratio of 3:1 in Figure S1. For details on the 2.0 mM NaOH condition, refer to Figure S1.

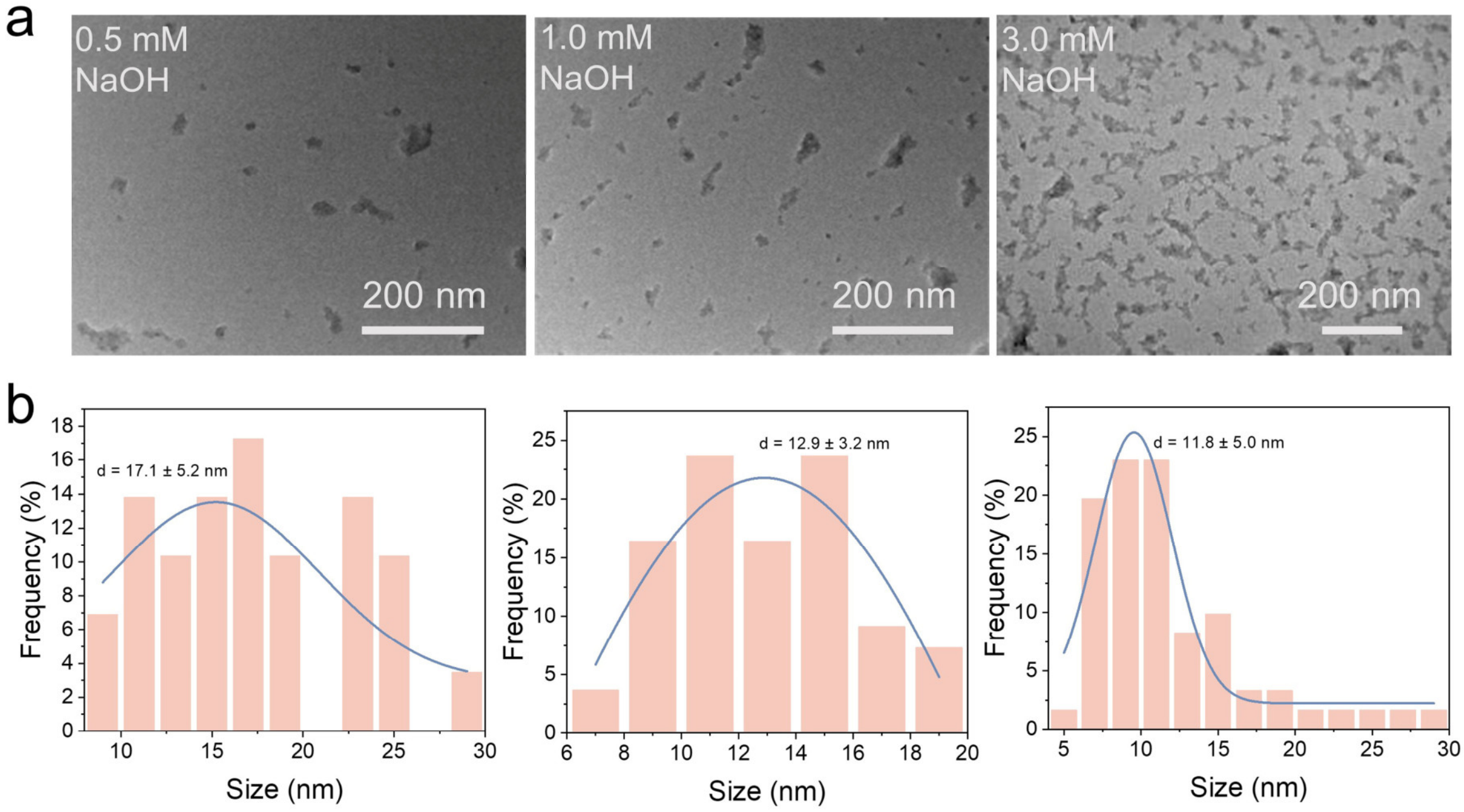

Figure S3. Adjustment of the temperature in the preparation of Cu-TMZ prodrugs. Polydispersity index (PDI) (a), TEM images (b) and size distribution (c) of Cu-TMZ prodrugs prepared with a fixed $Cu(NO_3)_2$-to-TMZ molar feed ratio of 3:1, NaOH concentration of 2.0 mM, reaction time of 6 h, and different temperature of 25 °C, 40 °C, and 60 °C.

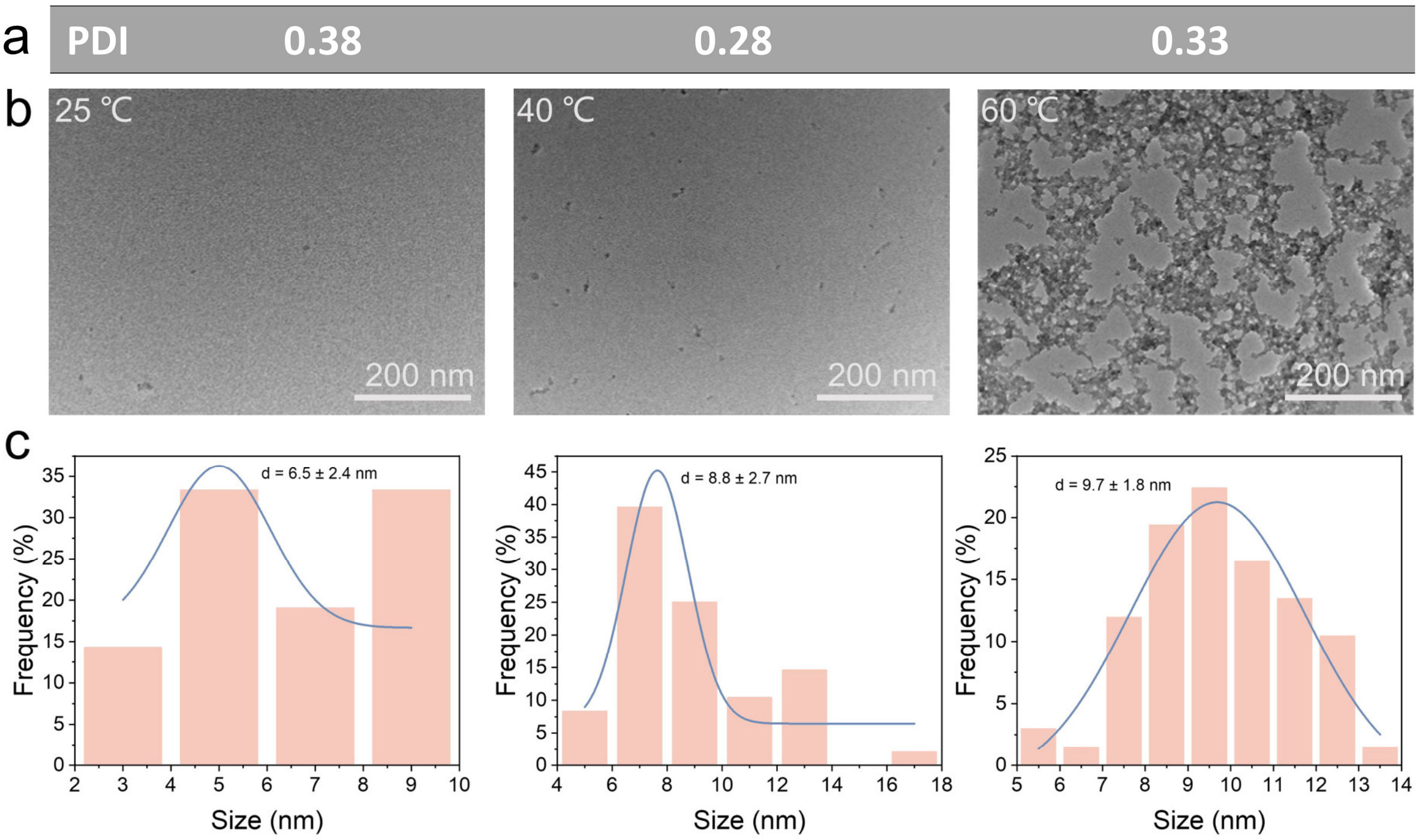

Figure S4. Adjustment of the reaction time in the preparation of Cu-TMZ prodrugs. PDI (a), TEM images (b) and size distribution (c) of Cu-TMZ prodrugs prepared with a fixed $Cu(NO_3)_2$-to-TMZ molar feed ratio of 3:1, NaOH concentration of 2.0 mM, and reaction temperature of 40 °C and different reaction time of 4 h, 10 h, and 24 h. Please note that the condition of 6 h is identical to the condition of 40 °C in Figure S3. For details on the 6 h condition, refer to Figure S3.

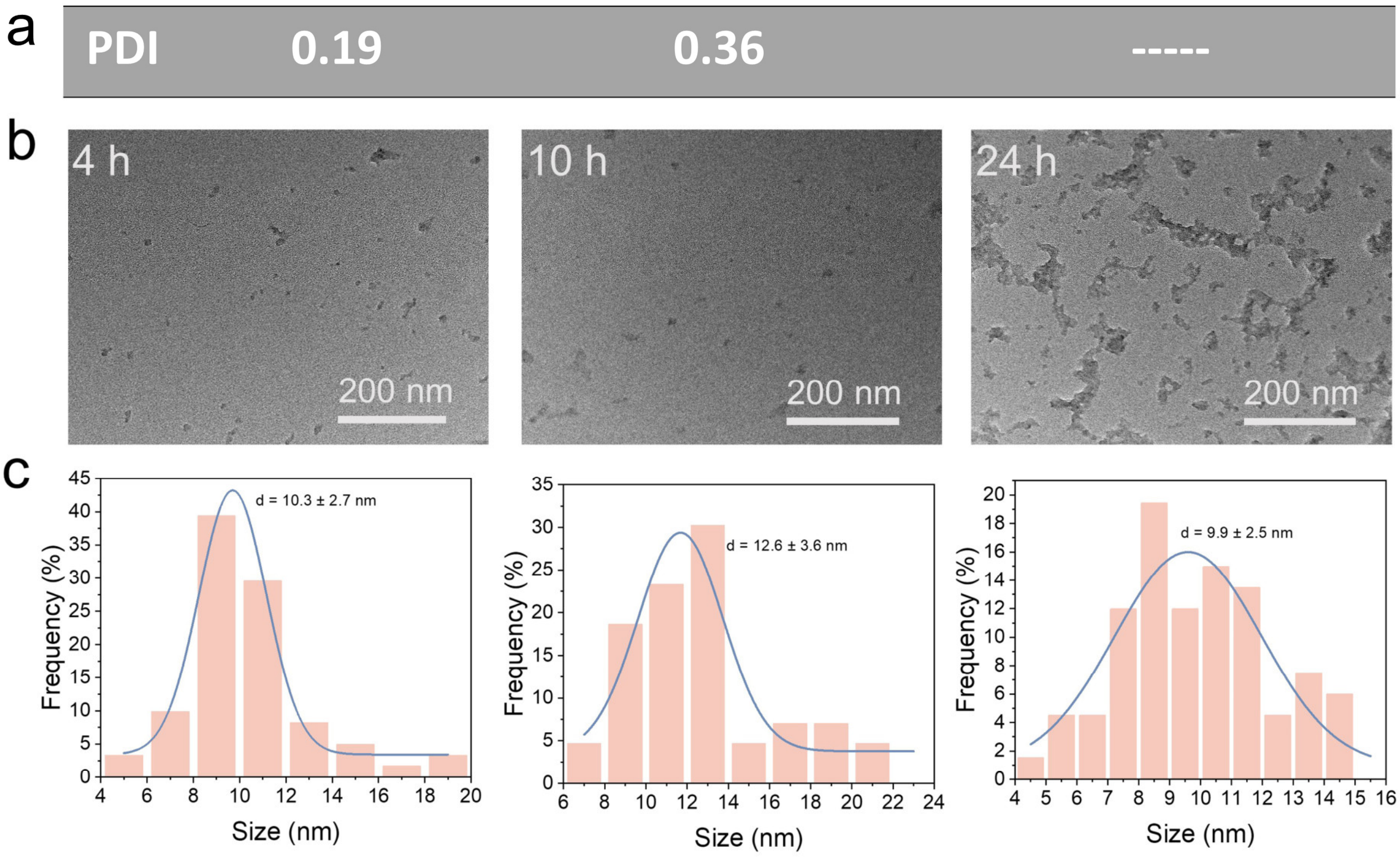

Table S1. Control of Cu-to-TMZ ratio in the as-prepared Cu-TMZ prodrugs by adjusting the molar feed ratio of $Cu(NO_3)_2$-to-TMZ during preparation.

Prodrug	$Cu(NO_3)_2$ concentration (mM)	TMZ concentration (mM)	Molar feed ratio (Cu : TMZ)	Component	Mass ratio	Molar ratio	N (%)	C (%)	H (%)	O (%)	Cu (%)
Cu-TMZ	1.0	1.0	1:1	Cu	1	2.3	21.5	16.8	2.8	20.9	38.0
				TMZ	1.3	1					
	2.0	1.0	2:1	Cu	1	2.4	21.3	16.5	2.6	20.3	39.3
				TMZ	1.3	1					
	3.0	1.0	3:1	Cu	1	2.6	20.2	15.7	2.5	21.3	40.3
				TMZ	1.2	1					
	4.0	1.0	4:1	Cu	1	2.2	21.1	16.2	2.5	25.6	34.6
				TMZ	1.4	1					
	5.0	1.0	5:1	Cu	1	2.1	20.6	13.9	2.3	30.0	33.2
				TMZ	1.4	1					
	0.5	1.0	1:2	Cu	1	0.8	29.4	23.3	3.3	25.5	20.5
				TMZ	3.8	1					
	0.33	1.0	1:3	Cu	1	0.9	28.7	23.5	3.4	24.8	30.0
				TMZ	3.3	1					
	0.25	1.0	1:4	Cu	1	0.7	28.6	23.5	3.3	28.4	30.5
				TMZ	4.1	1					

Figure S5. TEM size distribution of Cu-TMZ/MET prodrugs.

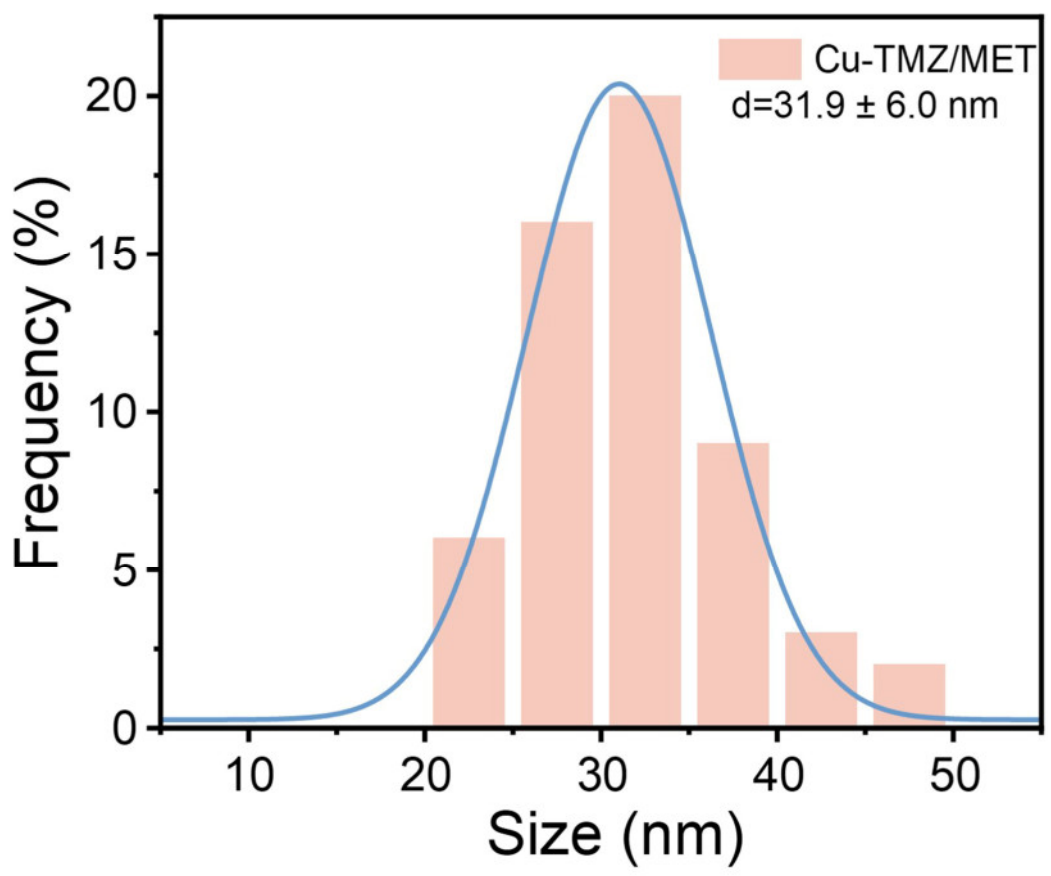

Figure S6. The XPS survey spectrum of Cu-TMZ/MET prodrugs.

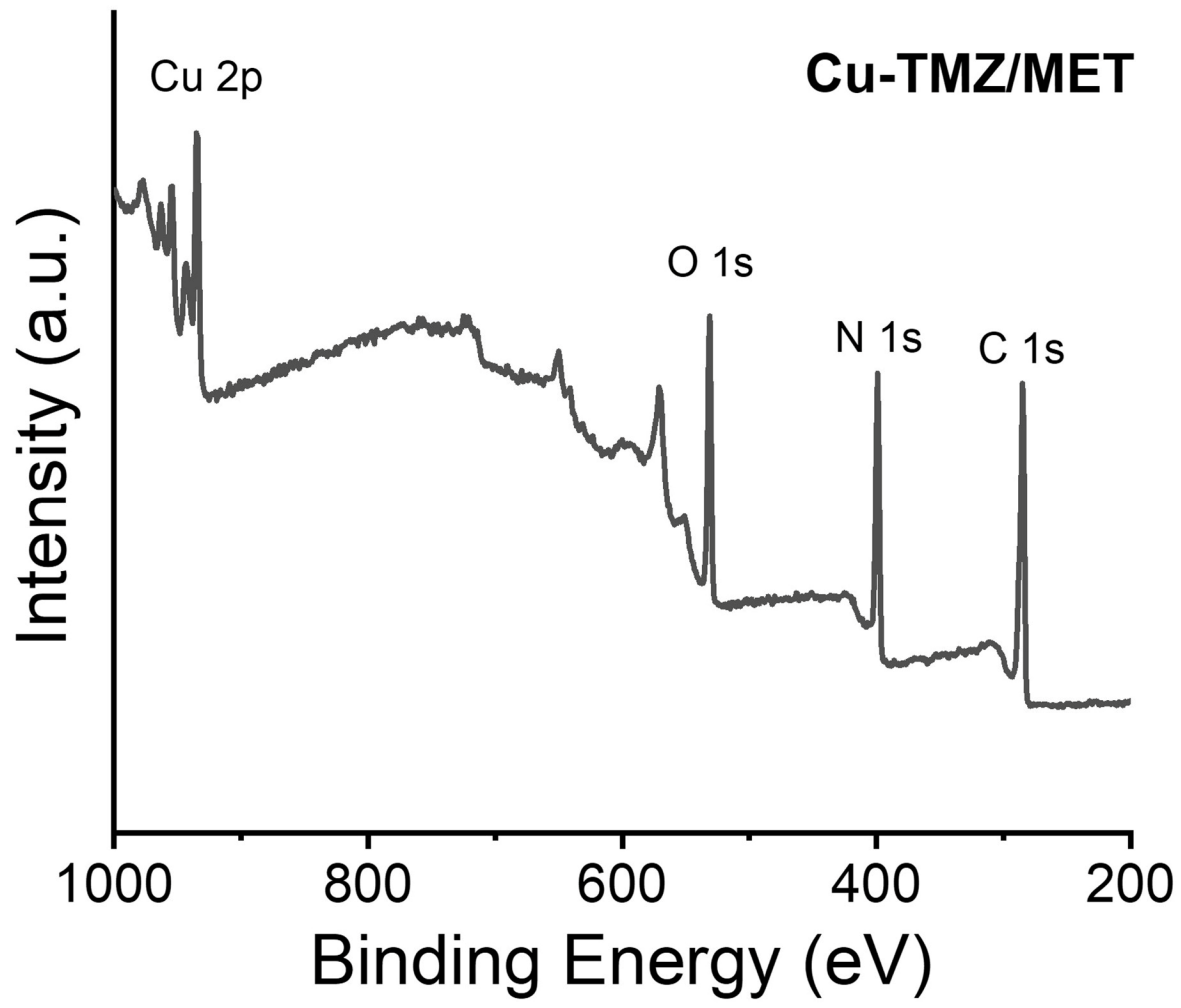

Table S2. Control of Cu-TMZ to MET ratio in the as-prepared Cu-TMZ/MET prodrugs by adjusting the mass feed ratio of Cu-TMZ to MET during preparation.

Prodrug	Cu-TMZ concentration (mg/mL)	MET concentration (mg/mL)	Mass feed ratio (Cu-TMZ : MET)	Component	Mass ratio	Molar ratio	N (%)	C (%)	H (%)	O (%)	Cu (%)
Cu-TMZ/MET	0.2	0.03	6:1	Cu	1	2.6	20.9	17.8	3.2	24.0	34.1
				TMZ	1.2	1					
				MET	0.7	0.9					
	0.2	0.1	2:1	Cu	1	2.6	23.4	18.9	3.3	16.3	38.0
				TMZ	1.2	1					
				MET	0.4	0.6					
	0.2	0.2	1:1	Cu	1	2.6	25.5	20.1	3.6	13.7	37.1
				TMZ	1.2	1					
				MET	0.5	0.6					
	0.2	0.4	1:2	Cu	1	2.6	28.1	21.5	3.8	17.0	29.6
				TMZ	1.2	1					
				MET	1.2	1.5					
	0.2	0.6	1:3	Cu	1	2.6	29.1	22.2	3.8	15.0	29.9
				TMZ	1.2	1					
				MET	1.1	1.5					
	0.2	10	1:5	Cu	1	2.6	29.5	22.8	3.9	17.1	26.6
				TMZ	1.2	1					
				MET	1.6	2.0					
	0.2	20	1:10	Cu	1	2.6	32.0	25.9	5.2	16.1	20.8
				TMZ	1.2	1					
				MET	2.6	3.4					

Table S3. Summaried parameters of Cu-TMZ and Cu-TMZ/MET prodrugs.

Prodrugs	Cu-TMZ	Cu-TMZ/MET
TEM mean size (nm)	10.3	31.9
Hydrodynamic diameter (nm)	72.4	105.9
Polydispersity index	0.19	0.23
Zeta potential (mV)	45.6	33.1
Cu:TMZ mass ratio	1:1.2	1:1.2
MET mass content	--	34.9%
Copper oxidation state	Cu(II)	Cu(II)

Figure S7. Temporal evolution of hydrated diameters of Cu-TMZ (a) and Cu-TMZ/MET (b) prodrugs (n=3). Data are presented as mean ± SD.

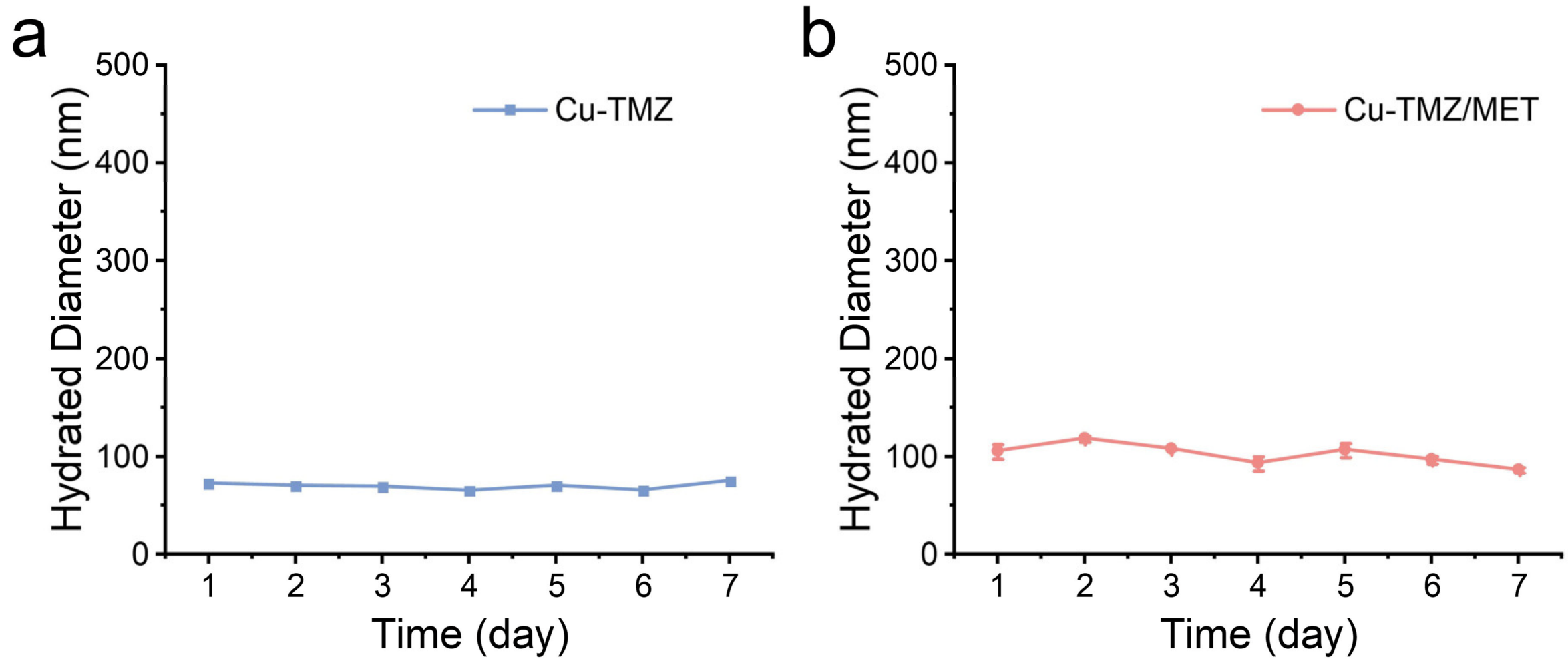

Figure S8. UV-vis absorption spectra (a), hydrated diameters (b), TEM images and corresponding photographs (c) of Cu-TMZ/MET prodrug solutions treated with different disrupting agents.

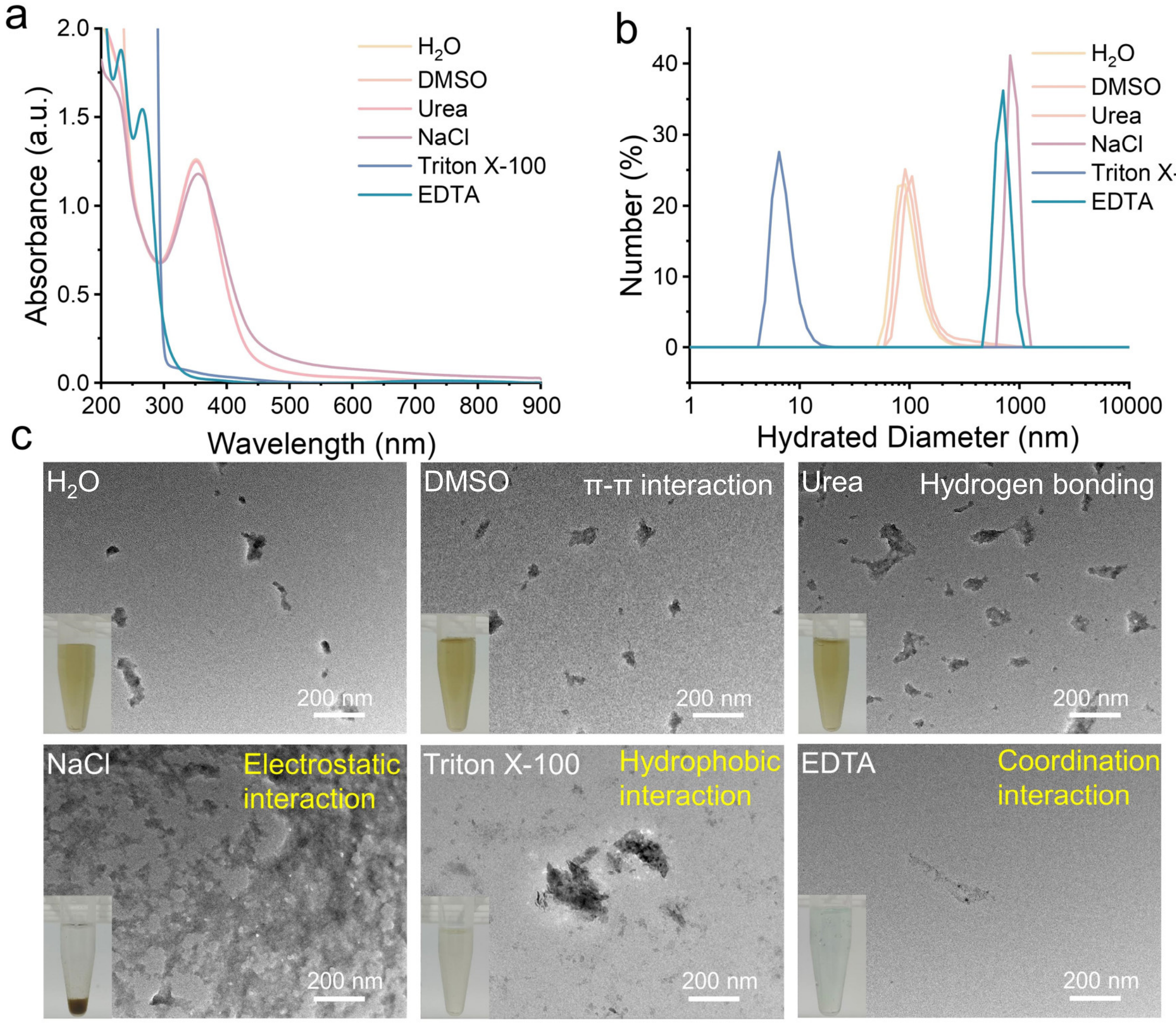

Figure S9. GSH depletion ability of prodrugs *via* detecting the fluorescence of OPA.

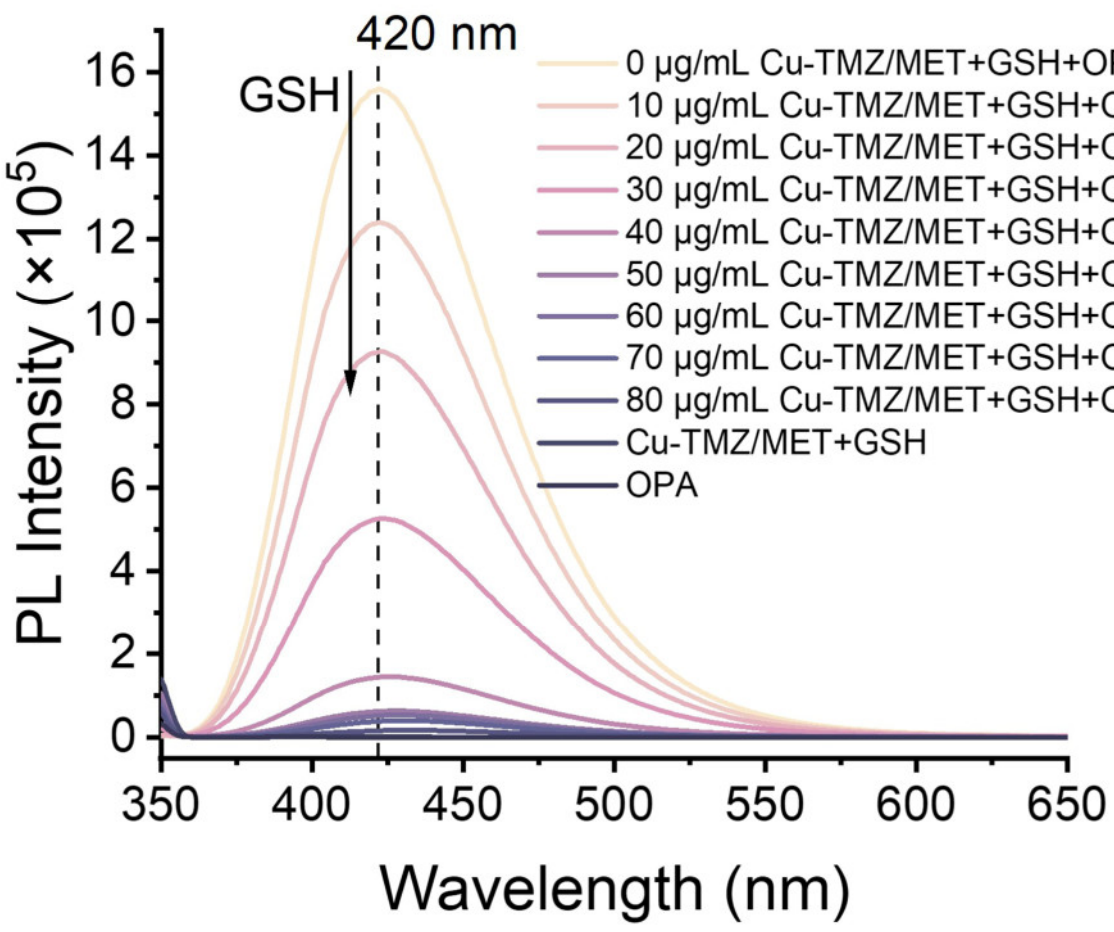

Figure S10. TEM images of Cu-TMZ/MET prodrugs incubated with GSH at 0 min, 5 min, 30 min, and 2 h.

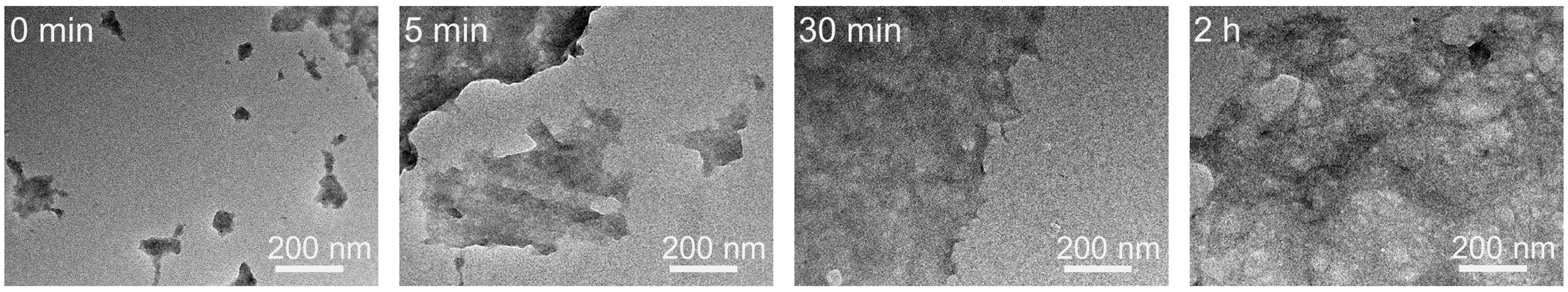

Figure S11. UV-vis absorption spectra of the products of disassembled prodrugs measured by Cu^{+} indicator neocuproine.

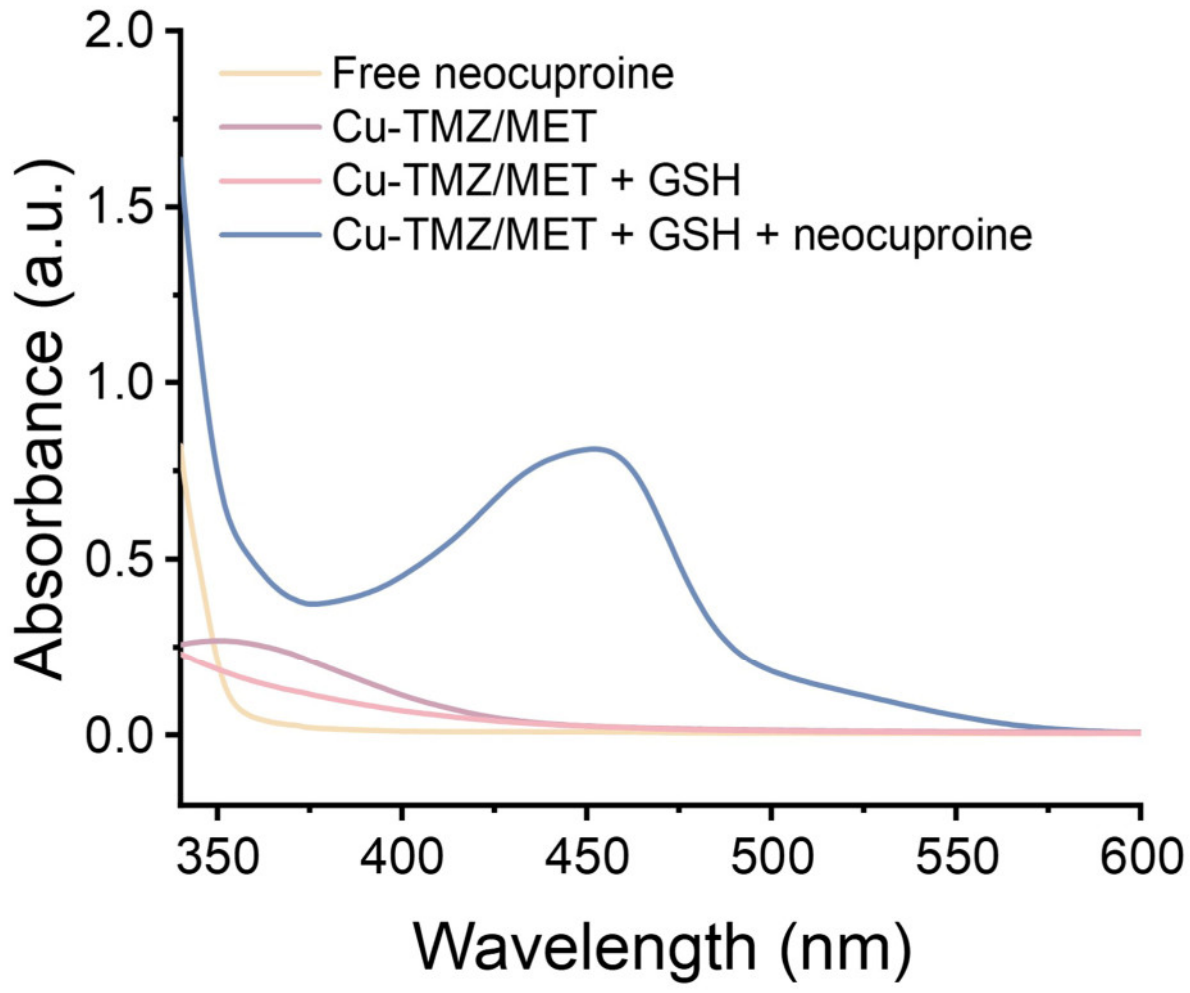

Figure S12. UV-vis absorption spectra of different concentrations of TMZ (a) and the standard absorption curve of TMZ (b). (c) Accumulative TMZ release of prodrugs with or without GSH (n=3). Data are presented as mean ± SD.

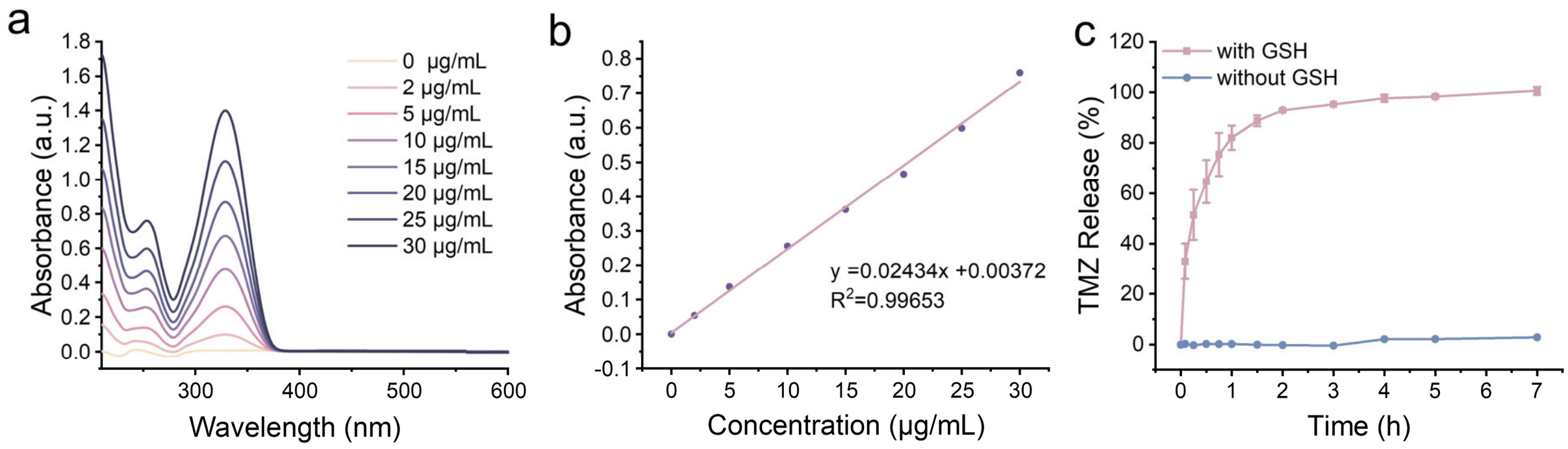

Figure S13. ·OH production ability of prodrugs. (a) The fluorescence spectra of TPA in different groups. (b) The UV-vis absorption spectra of MB in different groups.

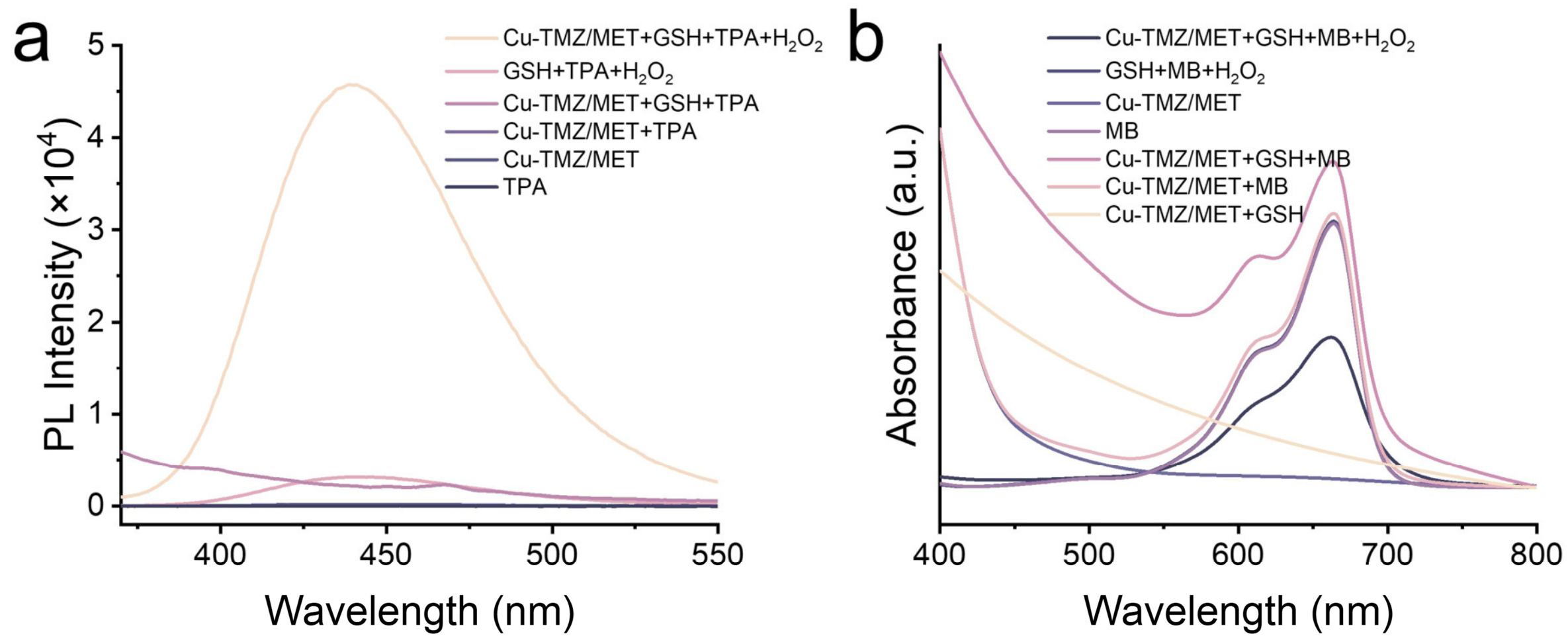

Figure S14. Illustration of GSH depletion and Fenton-like reaction of prodrugs.

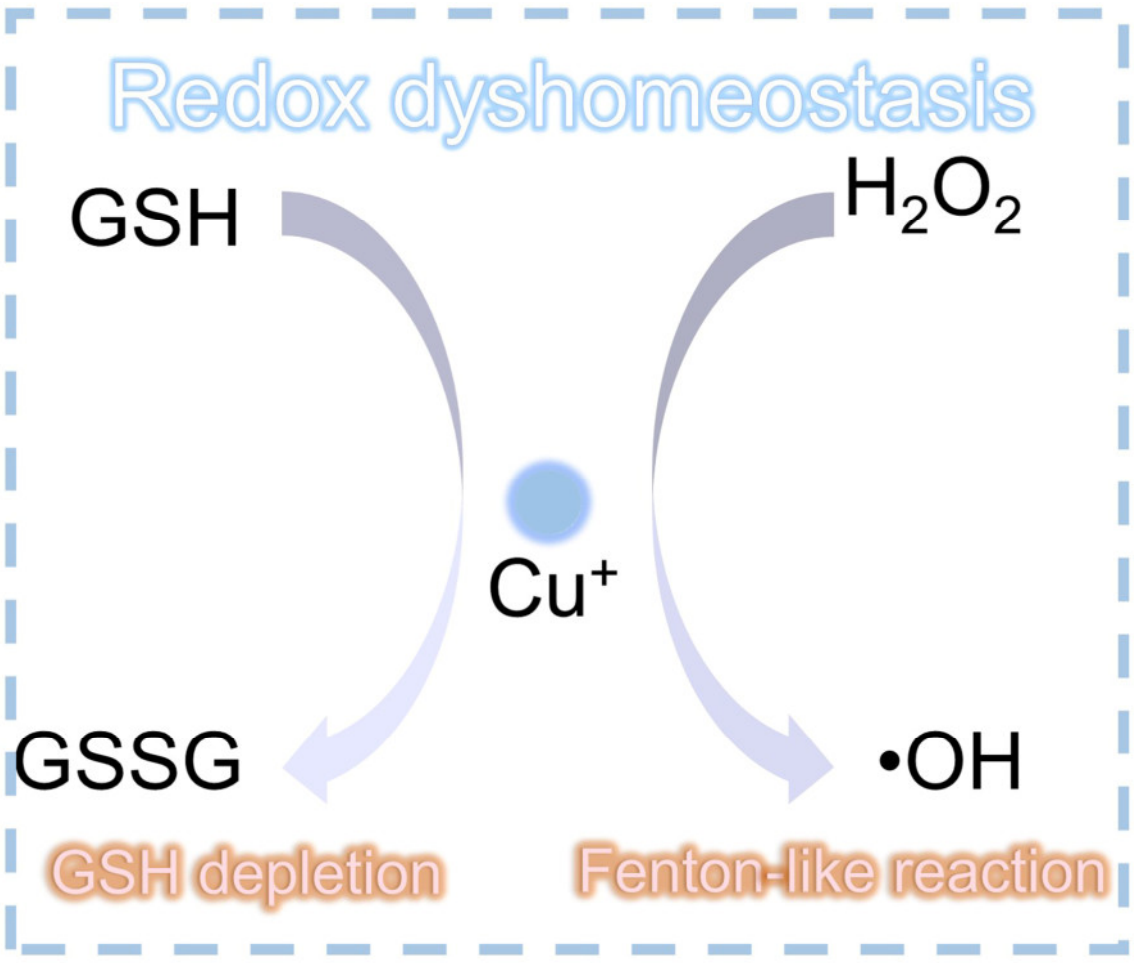

Figure S15. Fitted cell viability curves, as well as corresponding IC50 values and AUC values of U87 (a) and U87/TR (b) cells incubated with TMZ, Cu-TMZ prodrugs, and Cu-TMZ prodrugs plus 10 μg/mL or 20 μg/mL MET, derived from the data in Figure 2a and 2b (n=5). Cell viability (c), fitted cell viability curves, as well as corresponding IC50 value and AUC value (d) of U87 cells after incubation with TMZ at concentrations ranging from 0 to 300 μg/mL. Data are presented as mean ± SD. Statistical significances are calculated *via* one-way analysis of ANOVA test. ***$p < 0.001$.

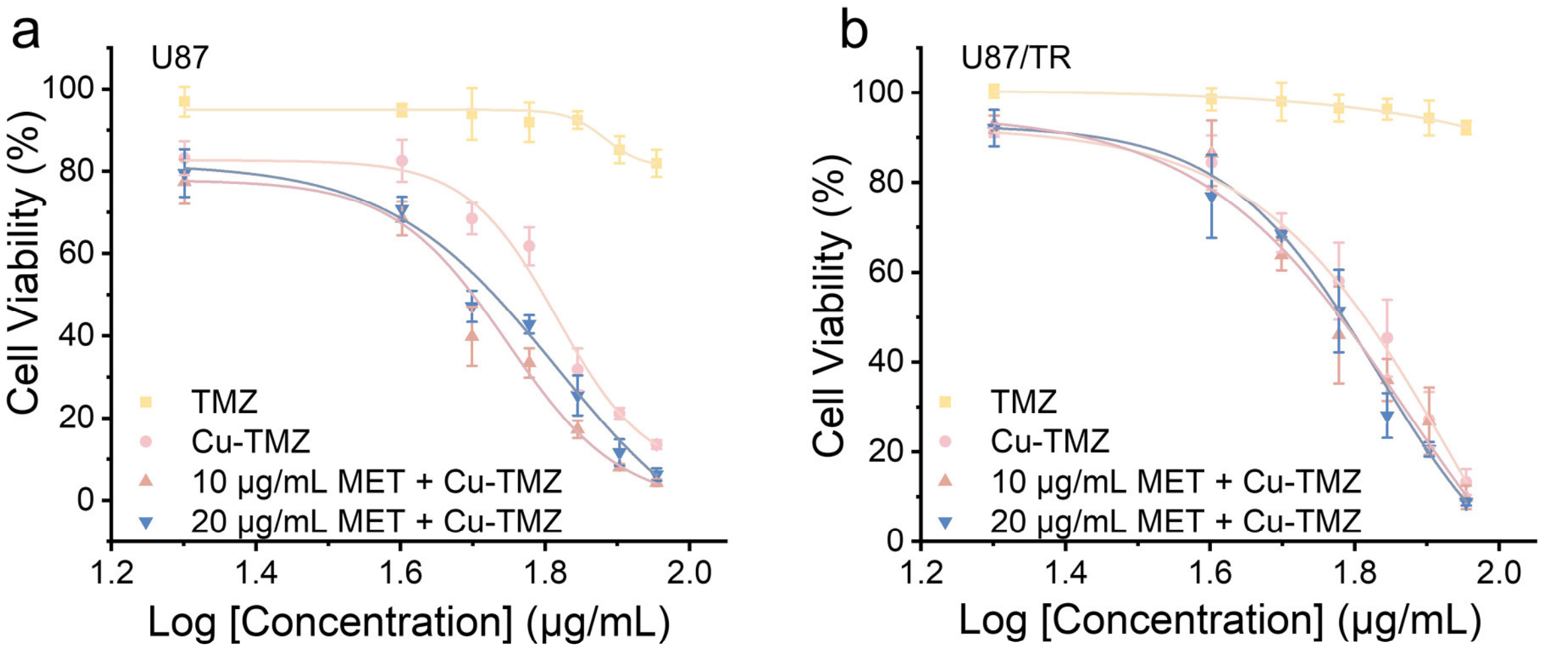

U87	TMZ	Cu-TMZ	10 μg/mL MET + Cu-TMZ	20 μg/mL MET + Cu-TMZ
IC50 (μg/mL)	N/A	62.8	50.1	53.7
AUC	61.0	42.9	34.1	36.9

U87/TR	TMZ	Cu-TMZ	10 μg/mL MET + Cu-TMZ	20 μg/mL MET + Cu-TMZ
IC50 (μg/mL)	N/A	64.6	60.3	60.7
AUC	64.0	45.3	43.4	44.0

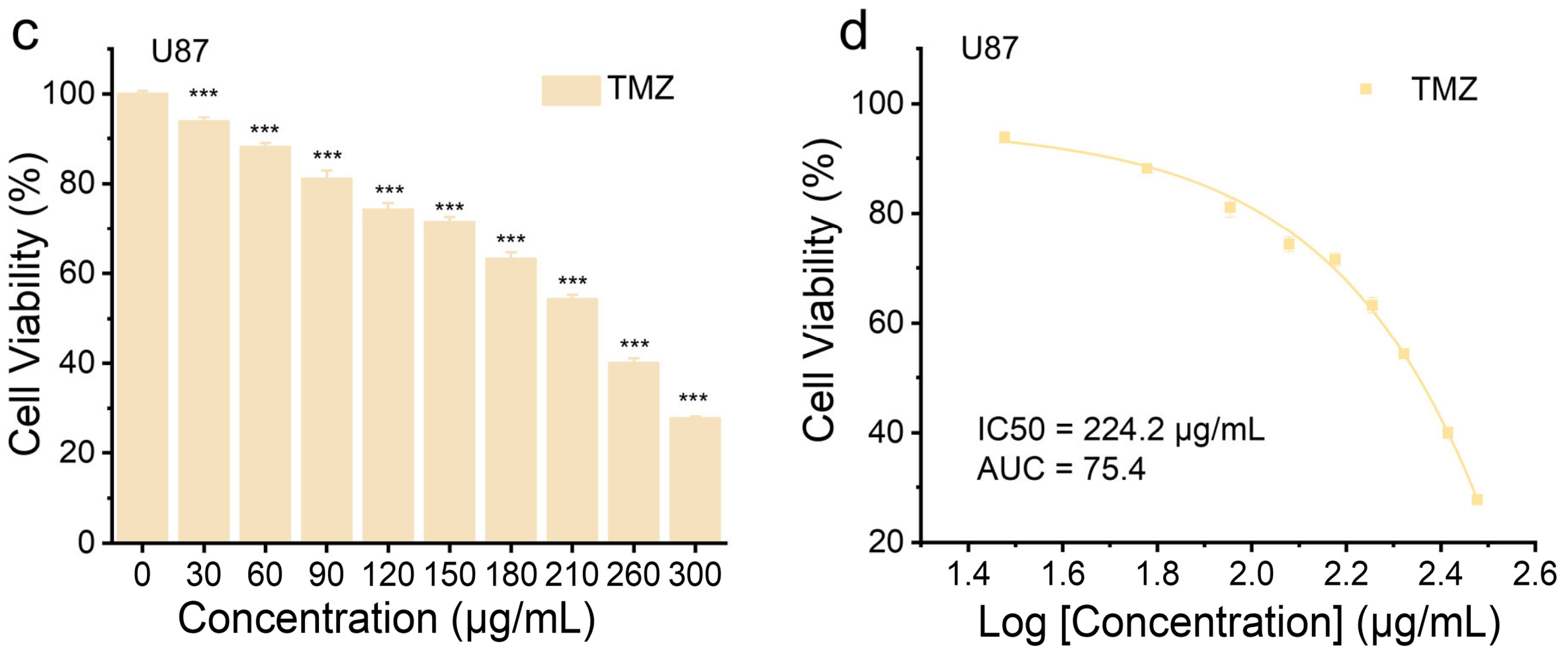

Table S4. Statistical analysis of U87 cells incubated with Cu-TMZ prodrugs, and Cu-TMZ prodrugs plus 10 μg/mL or 20 μg/mL MET, derived from the data in Figure 2a (n=5). Statistical significances are calculated *via* one-way analysis of ANOVA test. **p* < 0.05, ***p* < 0.01, and ****p* < 0.001, ns, not significant.

U87		Cu-TMZ								10 μg/mL MET + Cu-TMZ								20 μg/mL MET + Cu-TMZ							
	Concentration (μg/mL)	0	20	40	50	60	70	80	90	0	20	40	50	60	70	80	90	0	20	40	50	60	70	80	90
Cu-TMZ	0		***	***	***	***	***	***	***																
	20			ns	ns	***	***	***	***																
	40				ns	***	***	***	***																
	50					***	***	***	***																
	60						*	***	***																
	70							***	***																
	80								***																
10 μg/mL MET + Cu-TMZ	0										*	***	***	***	***	***	***								
	20											***	***	***	***	***	***								
	40												**	***	***	***	***								
	50													***	***	***	***								
	60														ns	***	***								
	70															***	***								
	80																***								
20 μg/mL MET + Cu-TMZ	0																		**	***	***	***	***	***	***
	20																			***	***	***	***	***	***
	40																				**	***	***	***	***
	50																					***	***	***	***
	60																						*	***	***
	70																							***	***
	80																								***

Table S5. Statistical analysis of U87/TR cells incubated with Cu-TMZ prodrugs, and Cu-TMZ prodrugs plus 10 μg/mL or 20 μg/mL MET, derived from the data in Figure 2b (n=5). Statistical significances are calculated *via* one-way analysis of ANOVA test. **p* < 0.05, ***p* < 0.01, and ****p* < 0.001.

U87/TR		Cu-TMZ								10 μg/mL MET + Cu-TMZ								20 μg/mL MET + Cu-TMZ							
	Concentration (μg/mL)	0	20	40	50	60	70	80	90	0	20	40	50	60	70	80	90	0	20	40	50	60	70	80	90
Cu-TMZ	0		***	***	***	***	***	***	***																
	20			*	***	***	***	***	***																
	40				***	***	***	***	***																
	50					*	***	***	***																
	60						*	***	***																
	70							**	***																
	80								***																
10 μg/mL MET + Cu-TMZ	0										***	**	***	***	***	***	***								
	20											*	***	***	***	***	***								
	40												***	***	***	***	***								
	50													**	***	***	***								
	60														*	**	***								
	70															*	***								
	80																***								
20 μg/mL MET + Cu-TMZ	0																		**	***	***	***	***	***	***
	20																			**	***	***	***	***	***
	40																				*	**	***	***	***
	50																					**	***	***	***
	60																						***	***	***
	70																							**	***
	80																								***

Figure S16. Viability and statistical analysis of U87 cells pretreated with the indicated compounds, including rotenone, UK 5099, or TTM, and then treated with Cu-TMZ prodrugs (n=5). Data are presented as mean ± SD. Statistical significances are calculated *via* one-way ANOVA test. **p* < 0.05, ***p* < 0.01, and ****p* < 0.001, ns, not significant.

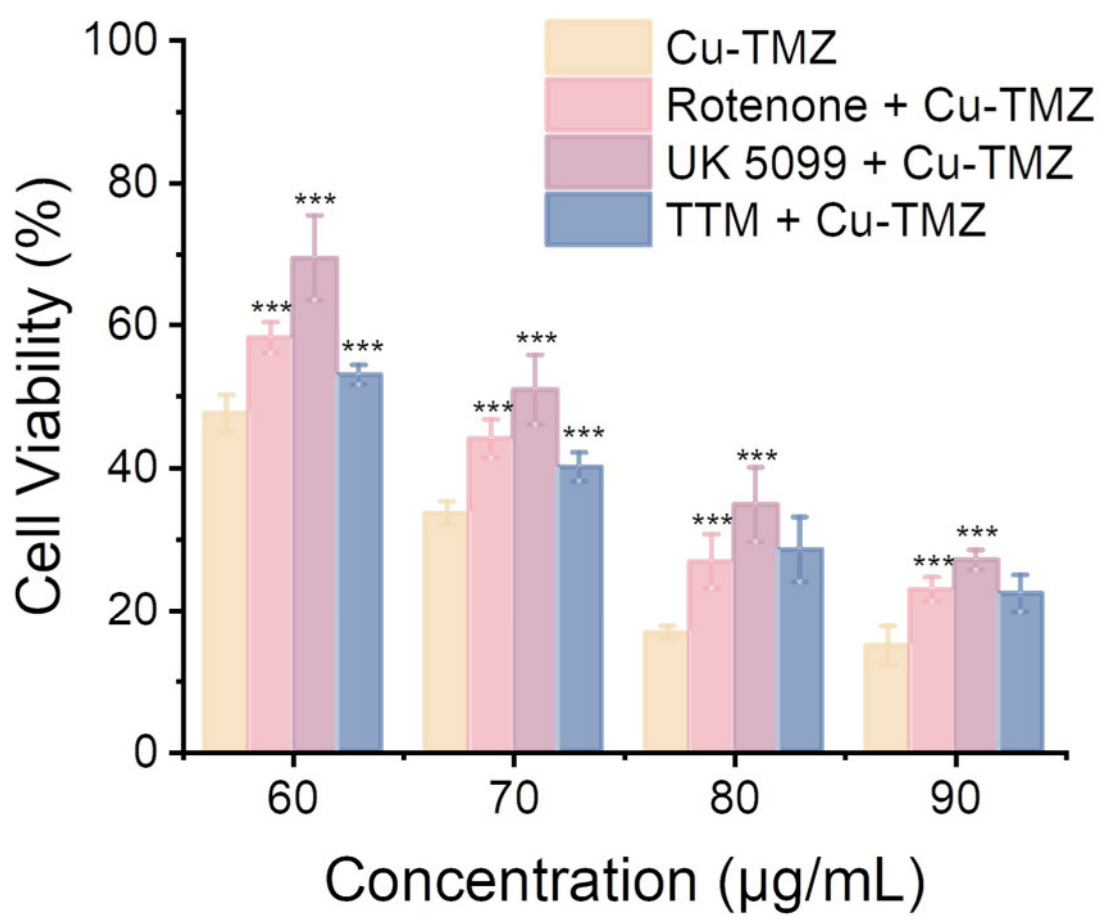

U87		Cu-TMZ				Rotenone + Cu-TMZ				UK 5099 + Cu-TMZ				TTM + Cu-TMZ			
	Concentration (μg/mL)	60	70	80	90	60	70	80	90	60	70	80	90	60	70	80	90
Cu-TMZ	60		***	***	***												
	70			***	***												
	80				ns												
Rotenone + Cu-TMZ	60						***	***	***								
	70							***	***								
	80								*								
UK 5099 + Cu-TMZ	60										***	***	***				
	70											***	***				
	80												**				
TTM + Cu-TMZ	60														***	***	***
	70															***	***
	80																*

Table S6. Statistical analysis of U87/TR cells pretreated with the indicated compounds, including rotenone, UK 5099, or TTM, and then treated with Cu-TMZ prodrugs, derived from the data in Figure 2c (n=5). Statistical significances are calculated *via* one-way ANOVA test. $^{**}p < 0.01$, and $^{***}p < 0.001$, ns, not significant.

U87/TR		Cu-TMZ				Rotenone + Cu-TMZ				UK 5099 + Cu-TMZ				TTM + Cu-TMZ			
	Concentration (μg/mL)	60	70	80	90	60	70	80	90	60	70	80	90	60	70	80	90
Cu-TMZ	60		***	***	***												
	70			***	***												
	80				***												
Rotenone + Cu-TMZ	60						ns	***	***								
	70							***	***								
	80								**								
UK 5099 + Cu-TMZ	60										**	***	***				
	70											***	***				
	80												**				
TTM + Cu-TMZ	60														***	***	***
	70															***	***
	80																***

Figure S17. Representative CLSM images showing the mitochondrial membrane potentials of JC-1 stained U87/TR cells after indicated treatments.

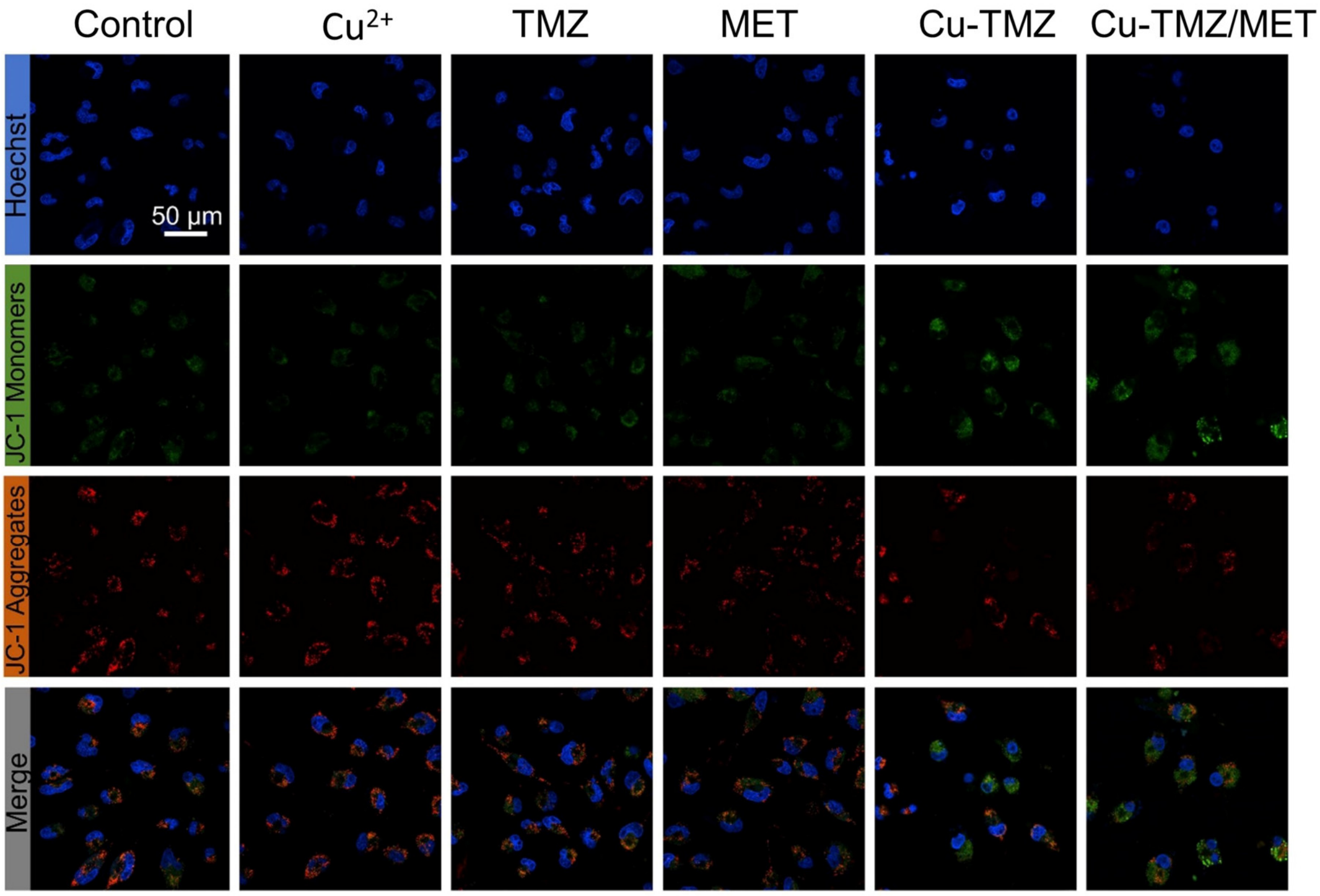

Figure S18. (a) UV-vis absorption spectra of MET, Cu-TMZ prodrugs, Cu-TMZ/MET-1 h, Cu-TMZ/MET-2 h, Cu-TMZ/MET-3 h, and Cu-TMZ/MET-4 h prodrugs. Viability (b), fitted cell viability curves, as well as corresponding IC50 values and AUC values (c) of U87 cells treated with Cu-TMZ, Cu-TMZ/MET-2 h, and Cu-TMZ/MET-3 h prodrugs (n=5). (d) Figure 2j fitted viability curves, as well as corresponding IC50 values and AUC values of U87/TR cells treated with Cu-TMZ, Cu-TMZ/MET-2 h, and Cu-TMZ/MET-3 h prodrugs (n=5). Cu-TMZ/MET-1 h, Cu-TMZ/MET-2 h, Cu-TMZ/MET-3 h, and Cu-TMZ/MET-4 h refer to the duration of 1 h, 2 h, 3 h, and 4 h for loading MET into Cu-TMZ prodrugs, respectively. Data are presented as mean ± SD. Statistical significances are calculated *via* one-way ANOVA test. $*p < 0.05$, and $***p < 0.001$.

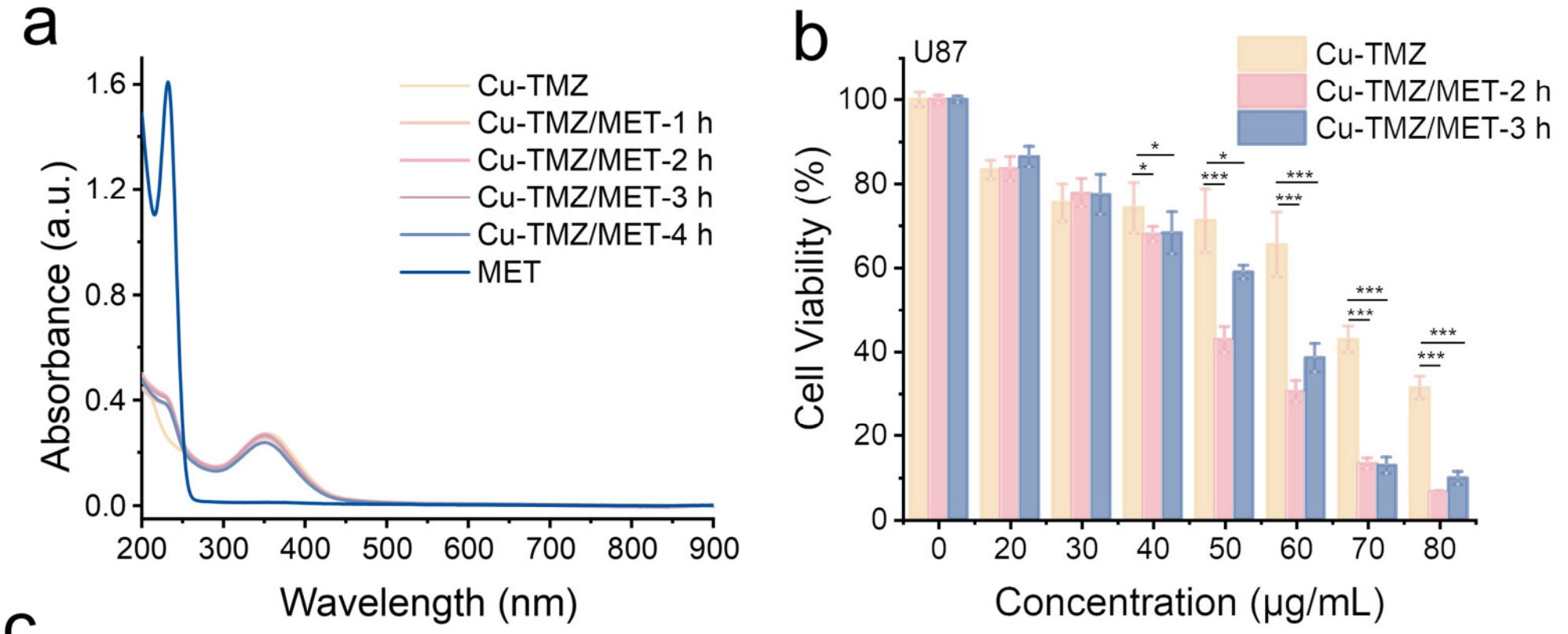

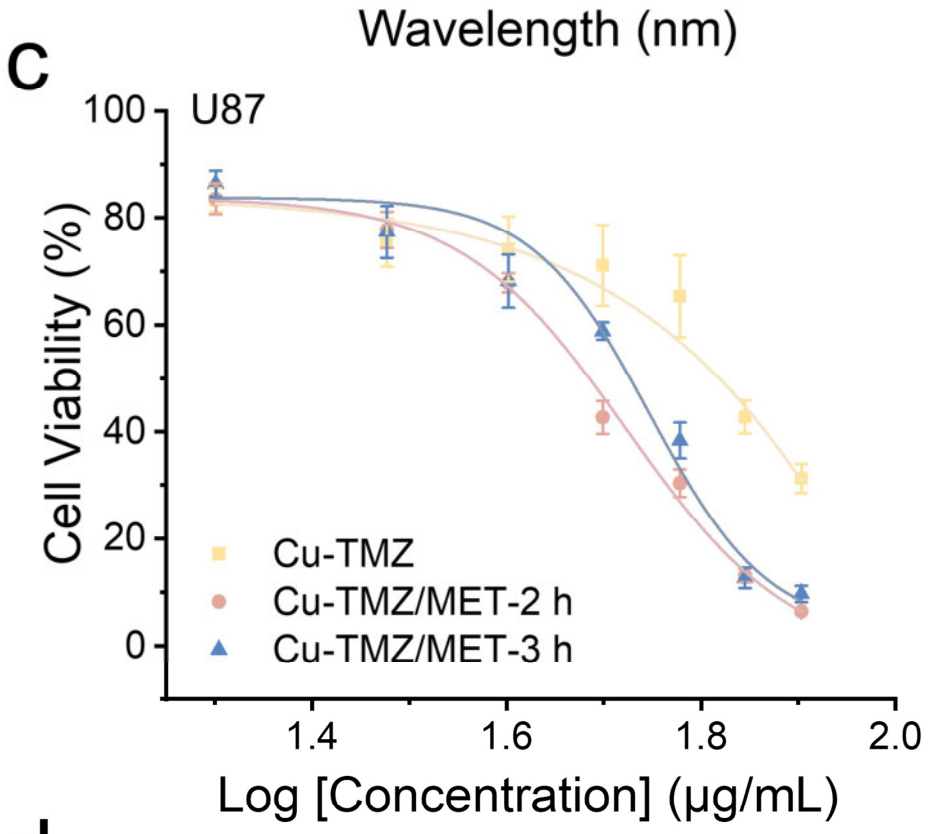

U87	Cu-TMZ	Cu-TMZ/MET-2 h	Cu-TMZ/MET-3 h
IC50 (μg/mL)	66.1	49.0	53.3
AUC	42.4	34.1	37.4

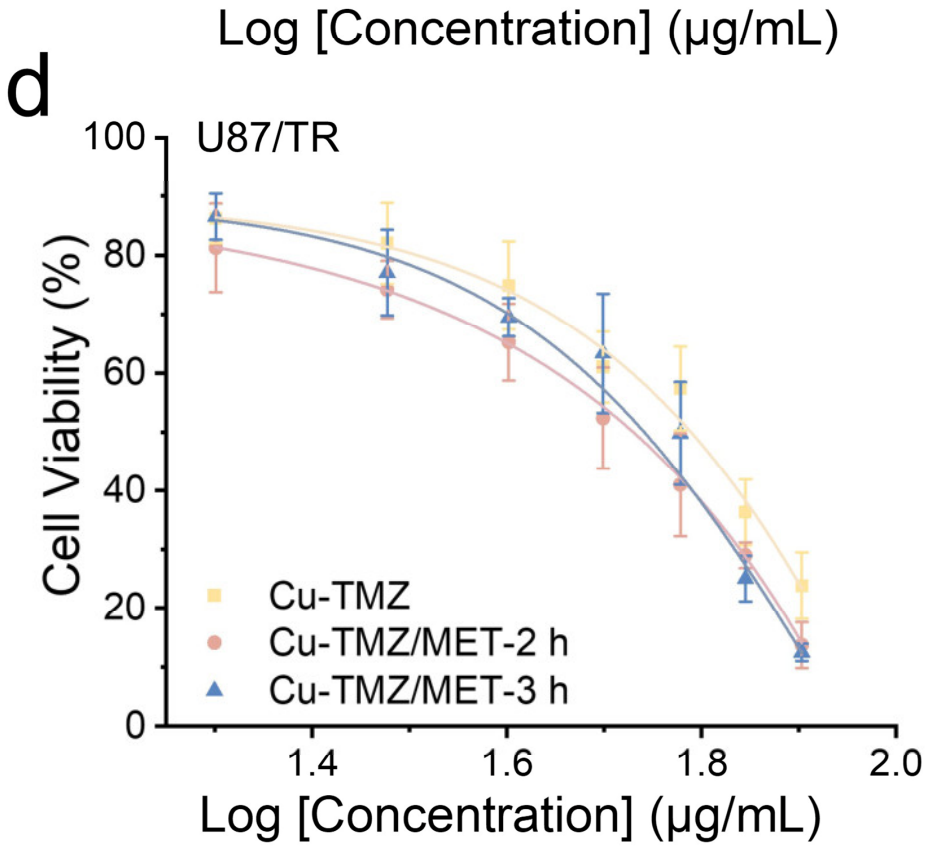

U87/TR	Cu-TMZ	Cu-TMZ/MET-2 h	Cu-TMZ/MET-3 h
IC50 (μg/mL)	61.7	53.7	55.0
AUC	40.8	35.8	37.8

Table S7. Statistical analysis of U87 cells treated with Cu-TMZ, Cu-TMZ/MET-2 h, and Cu-TMZ/MET-3 h prodrugs, derived from the data in Figure S18b (n=5). Statistical significances are calculated *via* one-way ANOVA test. *p < 0.05, **p < 0.01, and ***p < 0.001, ns, not significant.

U87		Cu-TMZ								Cu-TMZ/MET-2 h								Cu-TMZ/MET-3 h							
	Concentration (μg/mL)	0	20	40	50	60	70	80	90	0	20	40	50	60	70	80	90	0	20	40	50	60	70	80	90
Cu-TMZ	0		***	***	***	***	***	***	***																
	20			**	**	**	***	***	***																
	40				ns	ns	*	***	***																
	50					ns	*	***	***																
	60						ns	***	***																
	70							***	***																
	80								***																
Cu-TMZ/MET-2 h	0										***	***	***	***	***	***	***								
	20											**	***	***	***	***	***								
	40												***	***	***	***	***								
	50													***	***	***	***								
	60														***	***	***								
	70															***	***								
	80																***								
Cu-TMZ/MET-3 h	0																		***	***	***	***	***	***	***
	20																			**	***	***	***	***	***
	40																				*	***	***	***	***
	50																					**	***	***	***
	60																						***	***	***
	70																							***	***
	80																								*

Table S8. Statistical analysis of U87/TR cells treated with Cu-TMZ, Cu-TMZ/MET-2 h, and Cu-TMZ/MET-3 h prodrugs, derived from the data in Figure 2j (n=5). Statistical significances are calculated *via* one-way ANOVA test. **p* < 0.05, ***p* < 0.01, and ****p* < 0.001, ns, not significant.

U87/TR		Cu-TMZ								Cu-TMZ/MET-2 h								Cu-TMZ/MET-3 h							
	Concentration (μg/mL)	0	20	40	50	60	70	80	90	0	20	40	50	60	70	80	90	0	20	40	50	60	70	80	90
Cu-TMZ	0		***	***	***	***	***	***	***																
	20			*	***	***	***	***	***																
	40				*	*	***	***	***																
	50					ns	***	***	***																
	60						*	***	***																
	70							***	***																
	80								***																
Cu-TMZ/MET-2 h	0										**	**	***	***	***	***	***								
	20											ns	ns	**	***	***	***								
	40												ns	*	**	***	***								
	50													*	***	***	***								
	60														ns	*	**								
	70															ns	**								
	80																ns								
Cu-TMZ/MET-3 h	0																		***	***	***	***	***	***	***
	20																			***	***	***	***	***	***
	40																				ns	***	***	***	***
	50																					***	**	***	***
	60																						ns	***	***
	70																							*	***
	80																								***

Figure S19. Comparison of the cell viability of U87/TR, bEnd.3, and L929 cells (a), fitted cell viability curves (d), as well as corresponding IC50 values and AUC values (g) after treatment with Cu-TMZ/MET-2 h prodrugs (n=5). Comparison of the cell viability of U87/TR, bEnd.3, and L929 cells (b), fitted cell viability curves (e), as well as corresponding IC50 values and AUC values (h) after treatment with Cu-TMZ/MET-3 h prodrugs (n=5). Comparison of the cell viability of U87/TR, bEnd.3, and L929 cells (c), fitted cell viability curves (f), as well as corresponding IC50 values and AUC values (i) after treatment with Cu-TMZ prodrugs (n=5). Cu-TMZ/MET-2 h, and Cu-TMZ/MET-3 h refer to the duration of 2 h, and 3 h for loading MET into Cu-TMZ prodrugs, respectively. Note that for a better comparison, some data in Figure S19 are identical to those in Figure S18 and 2j. Data are presented as mean ± SD. Statistical significances are calculated *via* one-way ANOVA test. *$p < 0.05$, **$p < 0.01$, and ***$p < 0.001$.

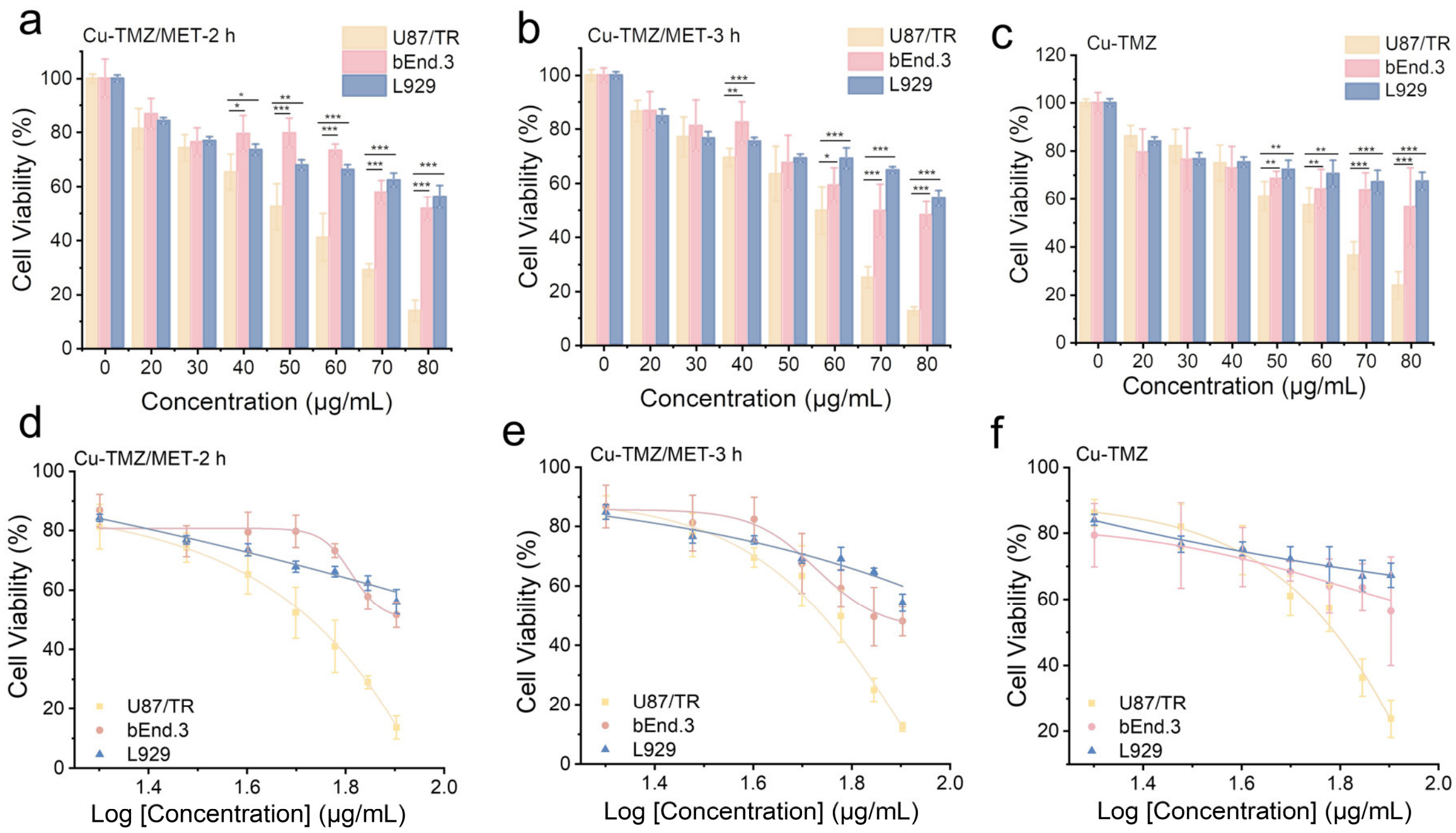

g

Cu-TMZ/MET-2 h	U87/TR	bEnd.3	L929
IC50 (μg/mL)	53.7	N/A	N/A
AUC	35.8	45.8	43.5

h

Cu-TMZ/MET-3 h	U87/TR	bEnd.3	L929
IC50 (μg/mL)	55.0	N/A	N/A
AUC	37.8	44.4	44.4

i

Cu-TMZ	U87/TR	bEnd.3	L929
IC50 (μg/mL)	61.7	N/A	N/A
AUC	40.8	43.0	45.1

Figure S20. Fluorescence spectra (a), and UV-vis absorption spectra (b) of FITC and ^{FITC}Cu-TMZ/MET prodrugs. (c) Representative CLSM images showing the internalization and lysosomal escape of ^{FITC}Cu-TMZ/MET prodrugs.

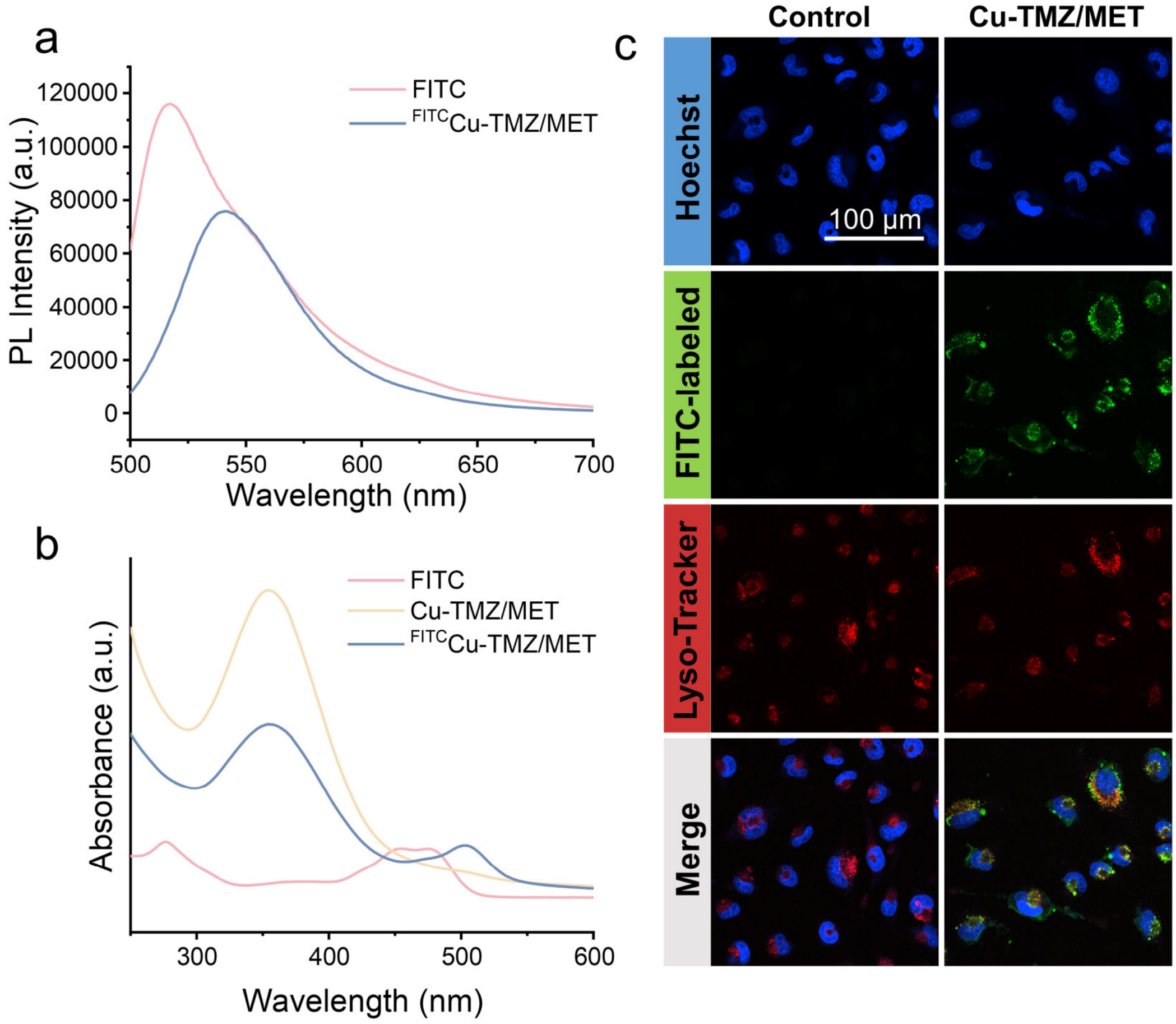

Figure S21. Representative CLSM images of U87/TR cells after calcein AM and PI staining upon different treatments.

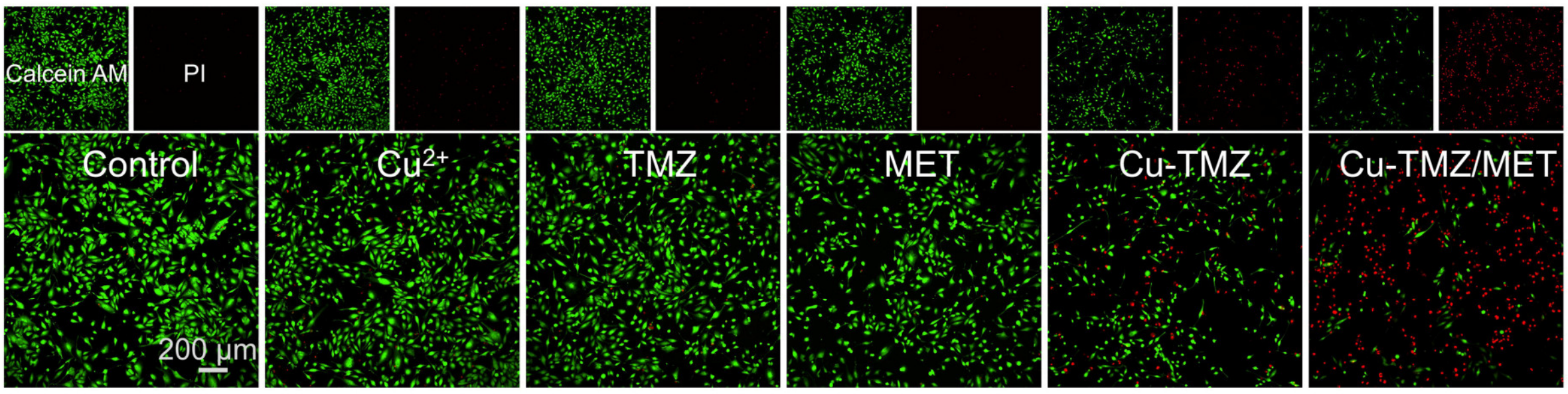

Figure S22. Viability of U87/TR cells treated with Cu-TMZ/MET prodrugs or AMPK inhibitor dorsomorphin plus Cu-TMZ/MET prodrugs (n=5). Data are presented as mean ± SD. Statistical significances are calculated *via* one-way ANOVA test. ***$p < 0.001$.

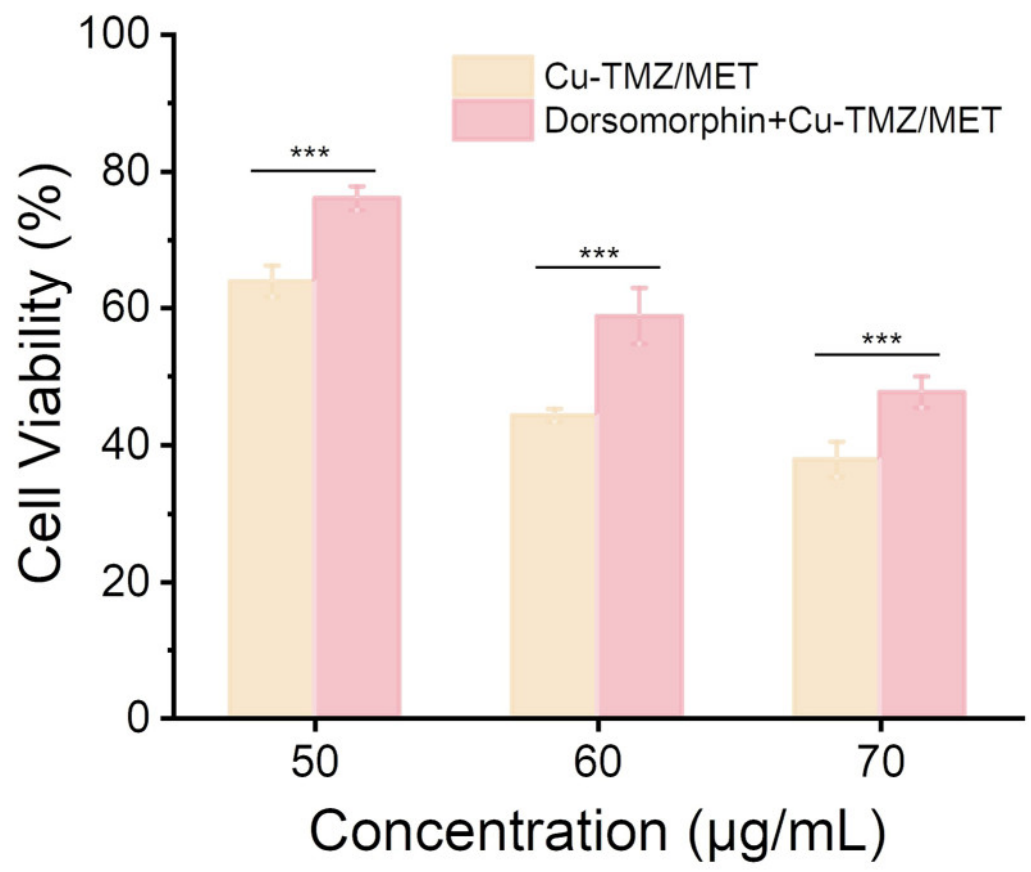

Figure S23. Representative CLSM images (a) and relative fluorescence intensity (b) showing the expression level of ATOX1 in U87/TR cells upon different treatments (n=3). Groups: (I) Control, (II) Cu^{2+}, (III) TMZ, (IV) MET, (V) Cu-TMZ, and (VI) Cu-TMZ/MET prodrugs. Data are presented as mean ± SD. Statistical significances are calculated *via* one-way ANOVA test. ***$p < 0.001$.

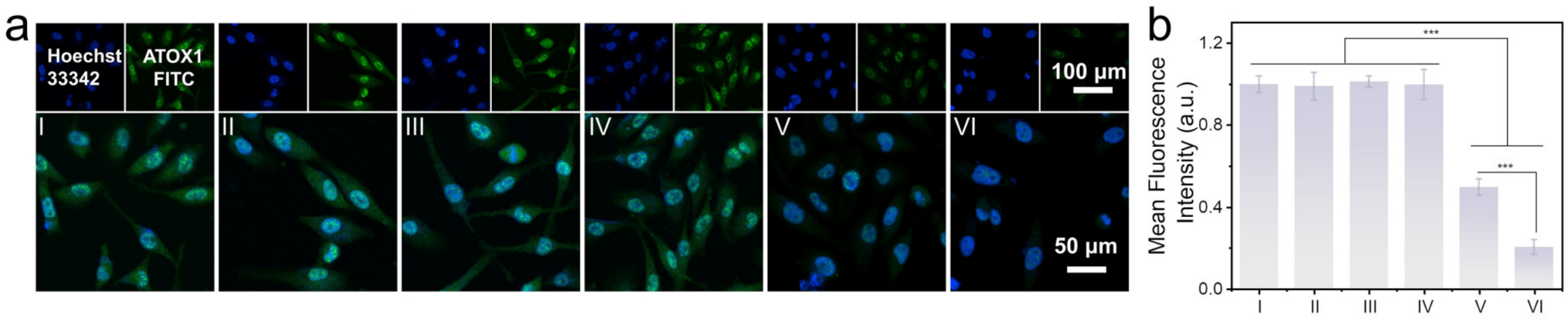

Figure S24. Quantitative analysis of tail DNA percentage in U87 (a) and U87/TR (b) cells after different treatments. Data are presented as mean ± SD. Statistical significances are calculated *via* one-way ANOVA test. ***$p < 0.001$, ns, not significant.

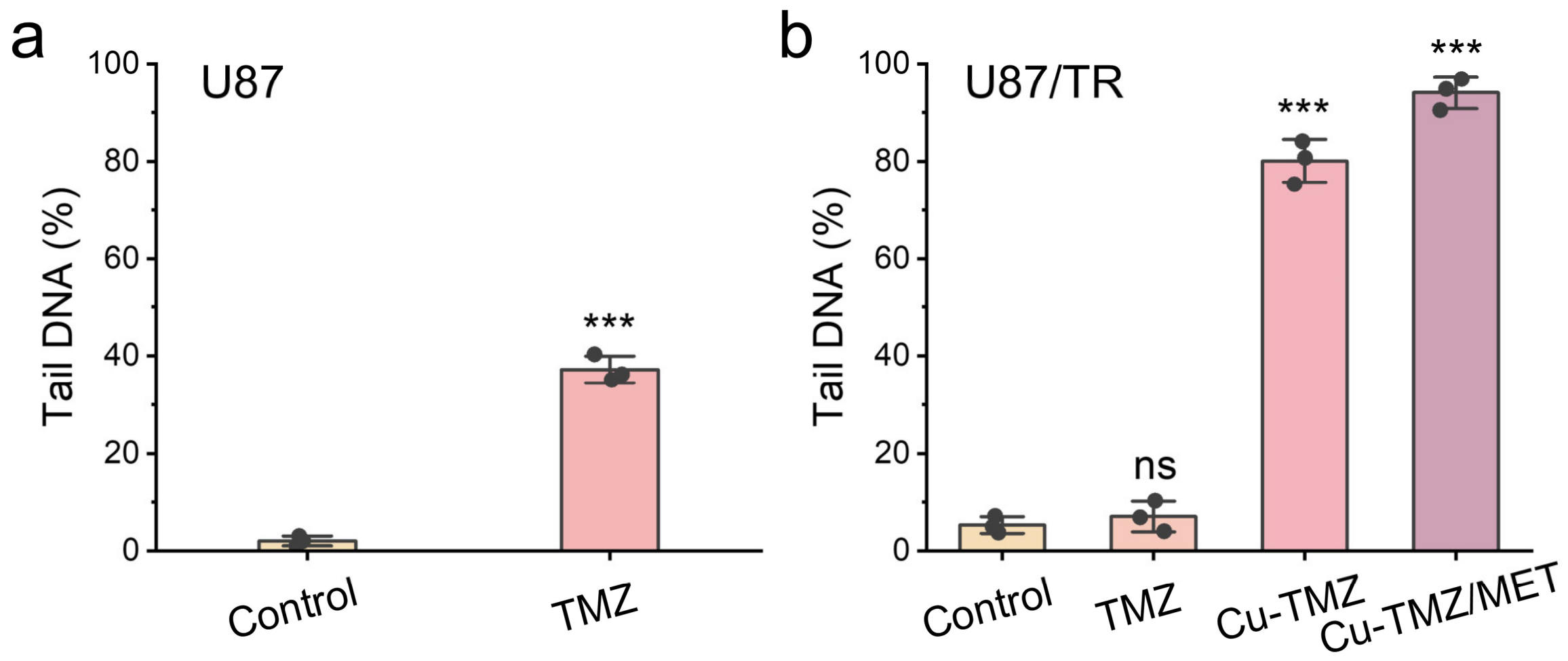

Figure S25. Flow cytometric analysis (a) and quantitative statistics (b) of cell cycle distribution in U87/TR cells after different treatments (n=3). Data are presented as mean ± SD. Statistical significances are calculated *via* one-way ANOVA test. $^{**}p < 0.01$, and $^{***}p < 0.001$.

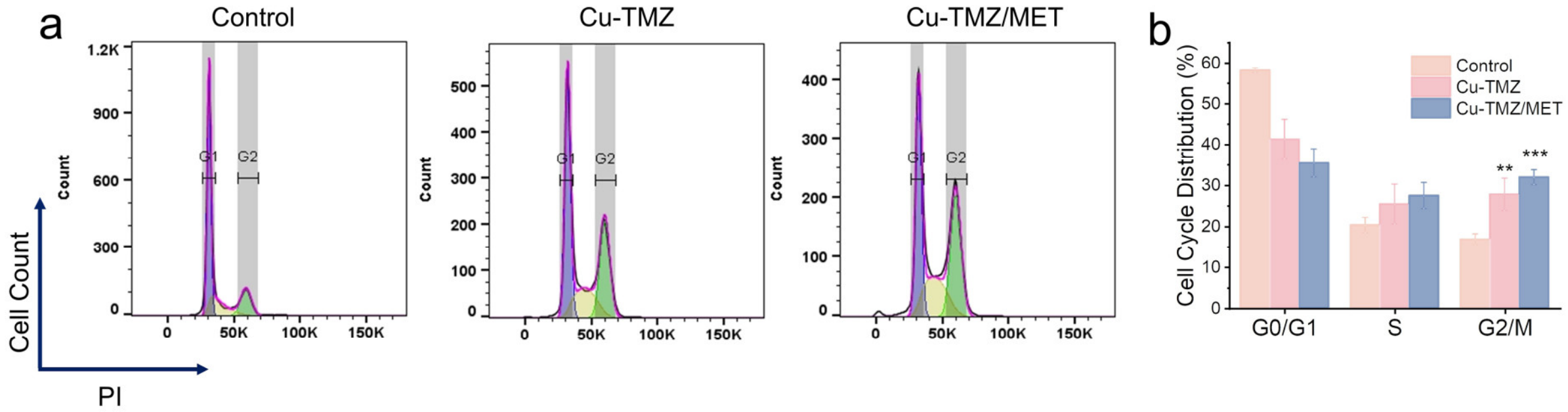

Figure S26. The expression levels of Cyclin B1, p21, and p-CDK1 in U87/TR cells by WB analysis after different treatments.

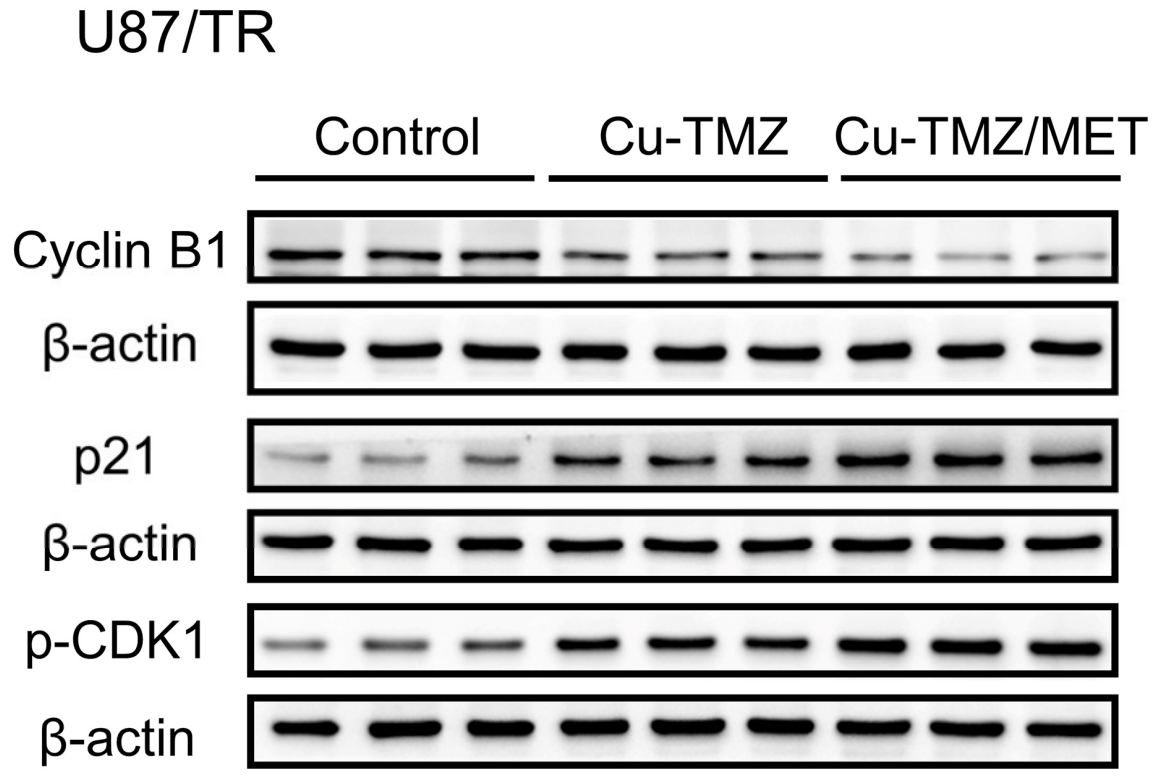

Figure S27. The expression levels of p53, p-p53, and MGMT in U87 and U87/TR cells by WB analysis.

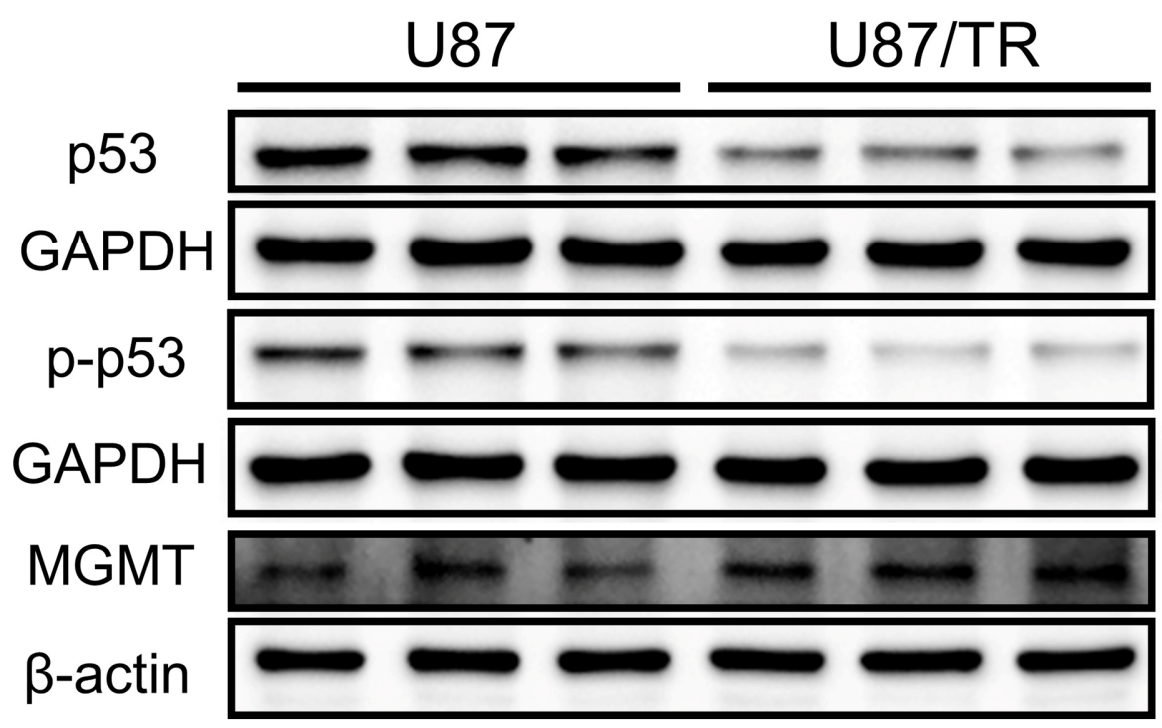

Figure S28. The expression levels of p53, and p-p53 in U87, U87/TR, and T98G cells by WB analysis.

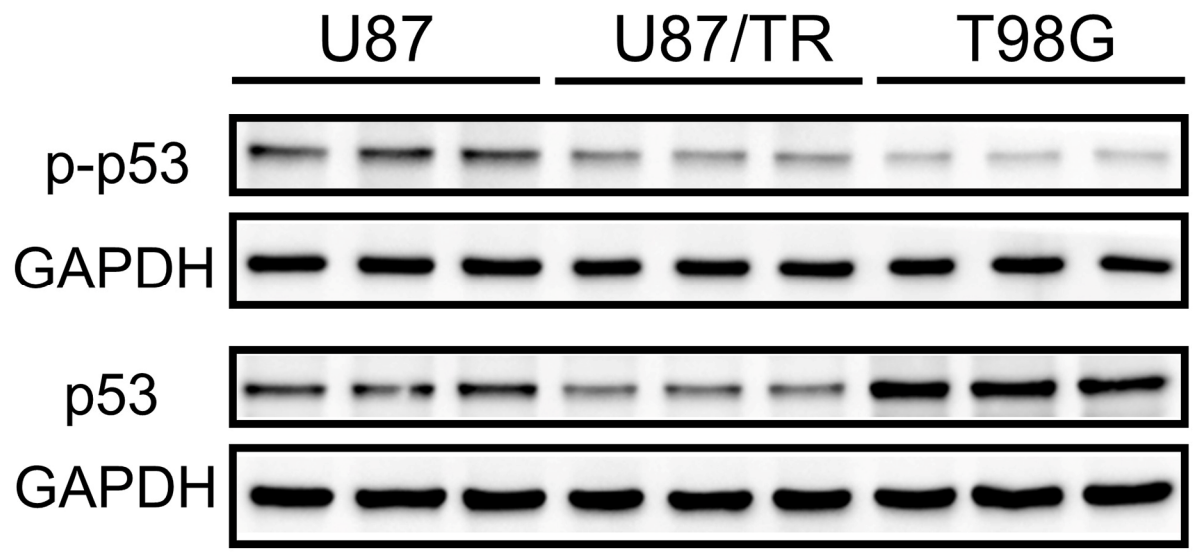

Figure S29. The expression levels of Cyclin B1, p-p53, p53, p21, p-CDK1, and MGMT in T98G cells by WB analysis after different treatments.

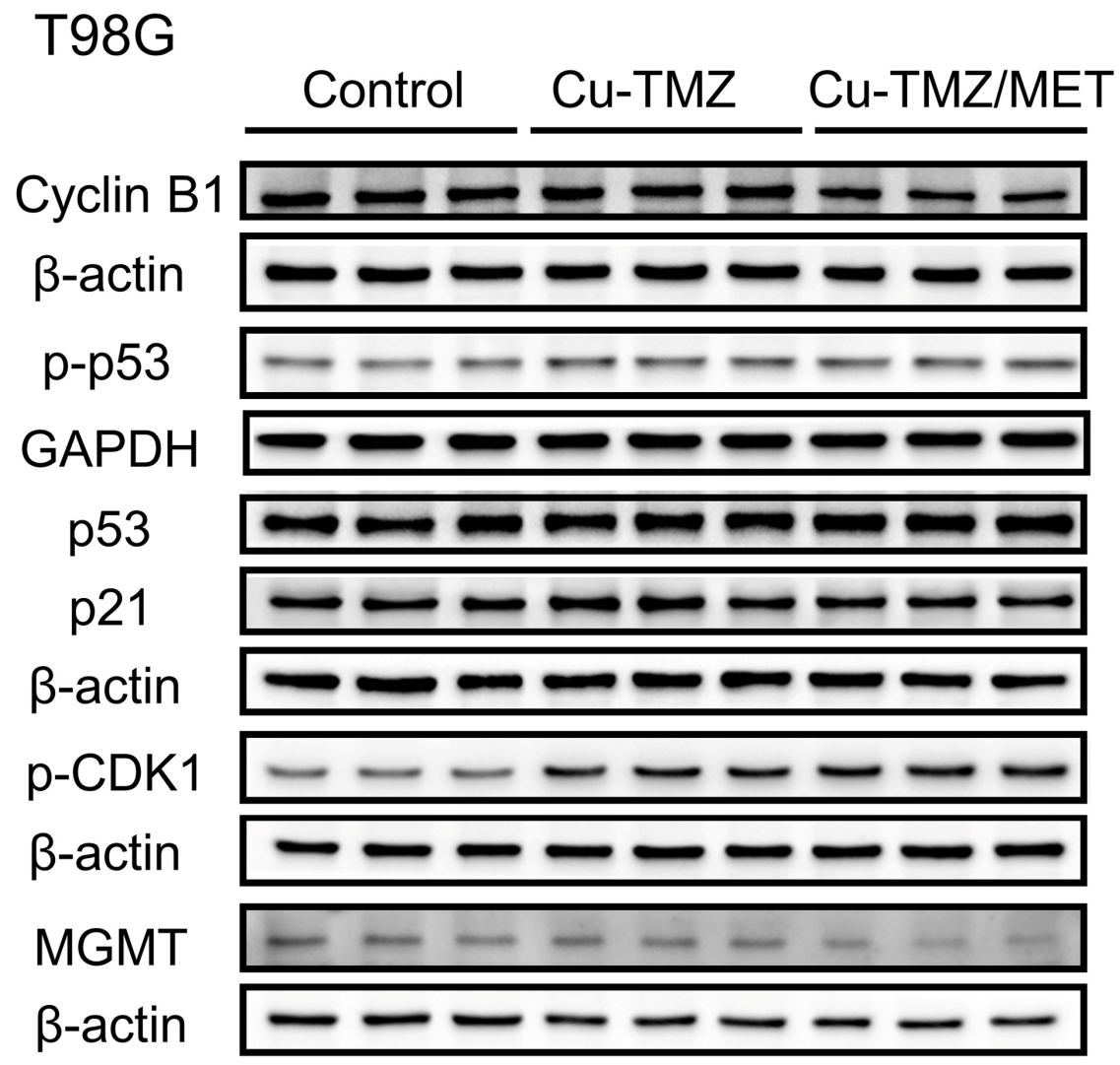

Figure S30. Viability (a), fitted cell viability curves with corresponding IC50 and AUC values (b), and statistical analysis (c) of A172 cells treated with Cu-TMZ or Cu-TMZ/MET prodrugs (n=5). Data are presented as mean ± SD. Statistical significances are calculated *via* one-way ANOVA test. **p* < 0.05, ***p* < 0.01, and ****p* < 0.001, ns, not significant.

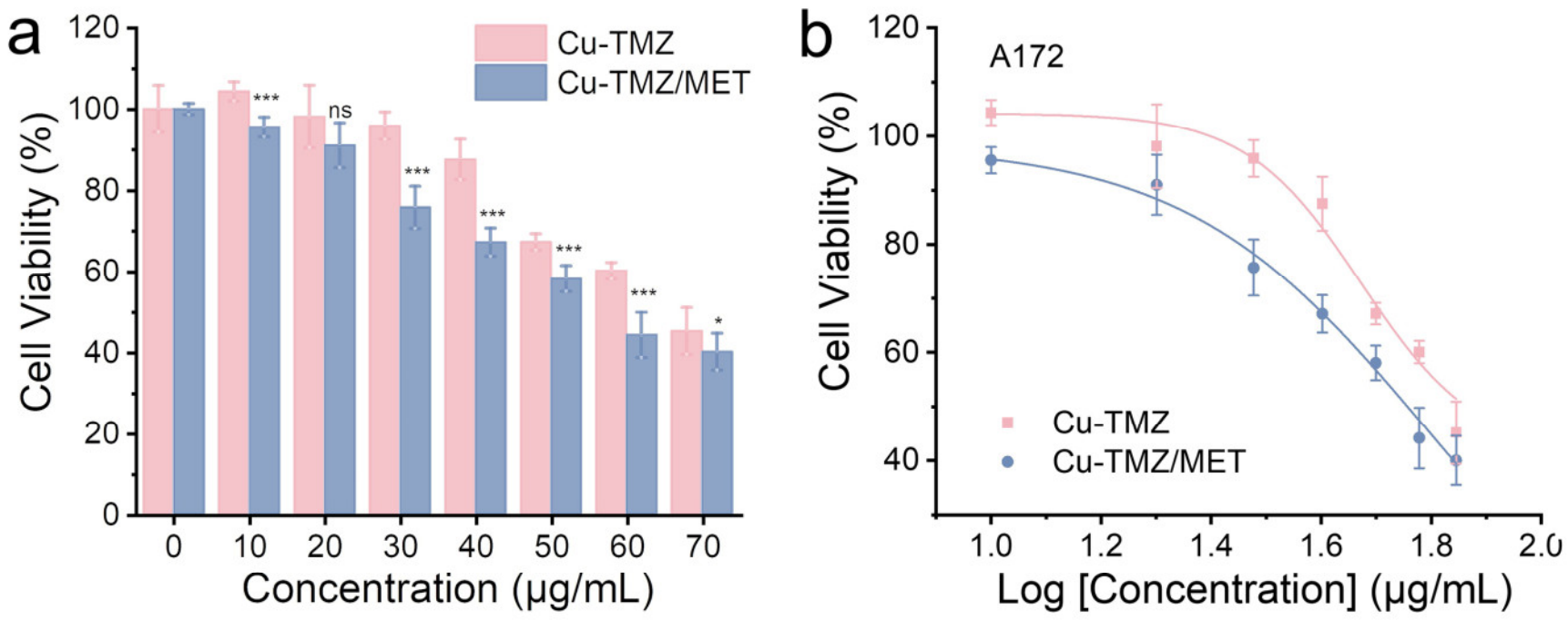

A172	Cu-TMZ	Cu-TMZ/MET
IC50 (μg/mL)	67.2	58.1
AUC	76.1	64.9

c

A172		Cu-TMZ								Cu-TMZ/MET							
	Concentration (μg/mL)	0	10	20	30	40	50	60	70	0	10	20	30	40	50	60	70
Cu-TMZ	0		ns	ns	ns	**	***	***	***								
	10			ns	**	***	***	***	***								
	20				ns	*	***	***	***								
	30					**	***	***	***								
	40						***	***	***								
	50							**	***								
	60								**								
Cu-TMZ/MET	0										**	*	***	***	***	***	***
	10											ns	***	***	***	***	***
	20												**	***	***	***	***
	30													**	***	***	***
	40														**	***	***
	50															***	***
	60																ns

Table S9. Top 50 differentially expressed genes for each contrast with the thresholds of padj < 0.05 and |log2fold change| > 0.

	Cu-TMZ vs Control		Cu-TMZ/MET vs Control		Cu-TMZ/MET vs Cu-TMZ	
	**Gene ID**	**Gene Name**	**Gene ID**	**Gene Name**	**Gene ID**	**Gene Name**
**1**	ENSG00000129244	ATP1B2	ENSG00000136925	TSTD2	ENSG00000185825	BCAP31
**2**	ENSG00000105229	PIAS4	ENSG00000070985	TRPM5	ENSG00000102158	MAGT1
**3**	ENSG00000260266	PPIAP46	ENSG00000150510	FAM124A	ENSG00000136942	RPL35
**4**	ENSG00000072182	ASIC4	ENSG00000261650	AC055717.2	ENSG00000149091	DGKZ
**5**	ENSG00000207063	SNORD116-1	ENSG00000138738	PRDM5	ENSG00000154589	LY96
**6**	ENSG00000188647	PTAR1	ENSG00000279500	AC108704.2	ENSG00000101216	GMEB2
**7**	ENSG00000162222	TTC9C	ENSG00000280670	CCDC163	ENSG00000089289	IGBP1
**8**	ENSG00000089902	RCOR1	ENSG00000005513	SOX8	ENSG00000108691	CCL2
**9**	ENSG00000269416	LINC01224	ENSG00000233766	AC098617.1	ENSG00000168615	ADAM9
**10**	ENSG00000130956	HABP4	ENSG00000164048	ZNF589	ENSG00000205060	SLC35B4
**11**	ENSG00000253309	SERPINE3	ENSG00000130649	CYP2E1	ENSG00000280077	AL353763.2
**12**	ENSG00000164530	PI16	ENSG00000229931	AL137003.1	ENSG00000078142	PIK3C3
**13**	ENSG00000184992	BRI3BP	ENSG00000237821	AC083873.1	ENSG00000089199	CHGB
**14**	ENSG00000239247	RN7SL589P	ENSG00000244038	DDOST	ENSG00000198492	YTHDF2
**15**	ENSG00000279170	AL137784.3	ENSG00000259431	THTPA	ENSG00000154719	MRPL39
**16**	ENSG00000237523	LINC00857	ENSG00000129103	SUMF2	ENSG00000167258	CDK12
**17**	ENSG00000095015	MAP3K1	ENSG00000160551	TAOK1	ENSG00000134769	DTNA
**18**	ENSG00000021645	NRXN3	ENSG00000138463	DIRC2	ENSG00000099901	RANBP1
**19**	ENSG00000226312	CFLAR-AS1	ENSG00000173093	CCDC63	ENSG00000171206	TRIM8
**20**	ENSG00000274963	RN7SL600P	ENSG00000269806	BICRA-AS1	ENSG00000100505	TRIM9
**21**	ENSG00000267391	MIR122HG	ENSG00000229097	CALM2P2	ENSG00000101608	MYL12A
**22**	ENSG00000196912	ANKRD36B	ENSG00000285928	AC103591.4	ENSG00000180879	SSR4
**23**	ENSG00000025772	TOMM34	ENSG00000163009	C2orf48	ENSG00000086475	SEPHS1
**24**	ENSG00000099331	MYO9B	ENSG00000011105	TSPAN9	ENSG00000121281	ADCY7
**25**	ENSG00000281649	EBLN3P	ENSG00000167840	ZNF232	ENSG00000074800	ENO1
**26**	ENSG00000280120	AC073857.1	ENSG00000132801	ZSWIM3	ENSG00000172943	PHF8
**27**	ENSG00000111666	CHPT1	ENSG00000111726	CMAS	ENSG00000204592	HLA-E
**28**	ENSG00000267265	AC011476.3	ENSG00000164975	SNAPC3	ENSG00000176165	FOXG1
**29**	ENSG00000163382	NAXE	ENSG00000262098	AC127496.2	ENSG00000170633	RNF34
**30**	ENSG00000177370	TIMM22	ENSG00000259366	AC108449.2	ENSG00000109180	OCIAD1
**31**	ENSG00000279080	AL022322.2	ENSG00000180346	TIGD2	ENSG00000084710	EFR3B
**32**	ENSG00000169371	SNUPN	ENSG00000137496	IL18BP	ENSG00000198753	PLXNB3
**33**	ENSG00000214827	MTCP1	ENSG00000276672	AL161891.1	ENSG00000181610	MRPS23
**34**	ENSG00000283376	AC011591.2	ENSG00000148832	PAOX	ENSG00000005483	KMT2E
**35**	ENSG00000139287	TPH2	ENSG00000276115	AC026356.2	ENSG00000169018	FEM1B
**36**	ENSG00000176531	PHLDB3	ENSG00000275888	AC132872.3	ENSG00000180448	ARHGAP45
**37**	ENSG00000176225	RTTN	ENSG00000236120	AC110995.1	ENSG00000132361	CLUH
**38**	ENSG00000102468	HTR2A	ENSG00000135930	EIF4E2	ENSG00000267325	LINC01415
**39**	ENSG00000260051	AL031600.1	ENSG00000259116	AL049869.3	ENSG00000110844	PRPF40B
**40**	ENSG00000111325	OGFOD2	ENSG00000285210	AL136382.1	ENSG00000165494	PCF11
**41**	ENSG00000173272	MZT2A	ENSG00000158486	DNAH3	ENSG00000152689	RASGRP3
**42**	ENSG00000188681	TEKT4P2	ENSG00000070886	EPHA8	ENSG00000231991	ANXA2P2
**43**	ENSG00000143742	SRP9	ENSG00000177707	NECTIN3	ENSG00000187908	DMBT1

**44**	ENSG00000113522	RAD50	ENSG00000152455	SUV39H2	ENSG00000148773	MKI67
**45**	ENSG00000155066	PROM2	ENSG00000132604	TERF2	ENSG00000134262	AP4B1
**46**	ENSG00000125347	IRF1	ENSG00000159593	NAE1	ENSG00000159882	ZNF230
**47**	ENSG00000141338	ABCA8	ENSG00000275011	AC129492.6	ENSG00000109111	SUPT6H
**48**	ENSG00000198055	GRK6	ENSG00000224609	HSD52	ENSG00000143476	DTL
**49**	ENSG00000278000	AC139100.2	ENSG00000251432	AC108062.1	ENSG00000077157	PPP1R12B
**50**	ENSG00000144677	CTDSPL	ENSG00000147421	HMBOX1	ENSG00000105221	AKT2

Figure S31. Volcano plot of the distributions of differentially expressed genes after Cu-TMZ, or Cu-TMZ/MET prodrug treatment (|$\log_2$fold change| > 2, P-value < 0.05). No differentially expressed genes are denoted as NS (n=3).

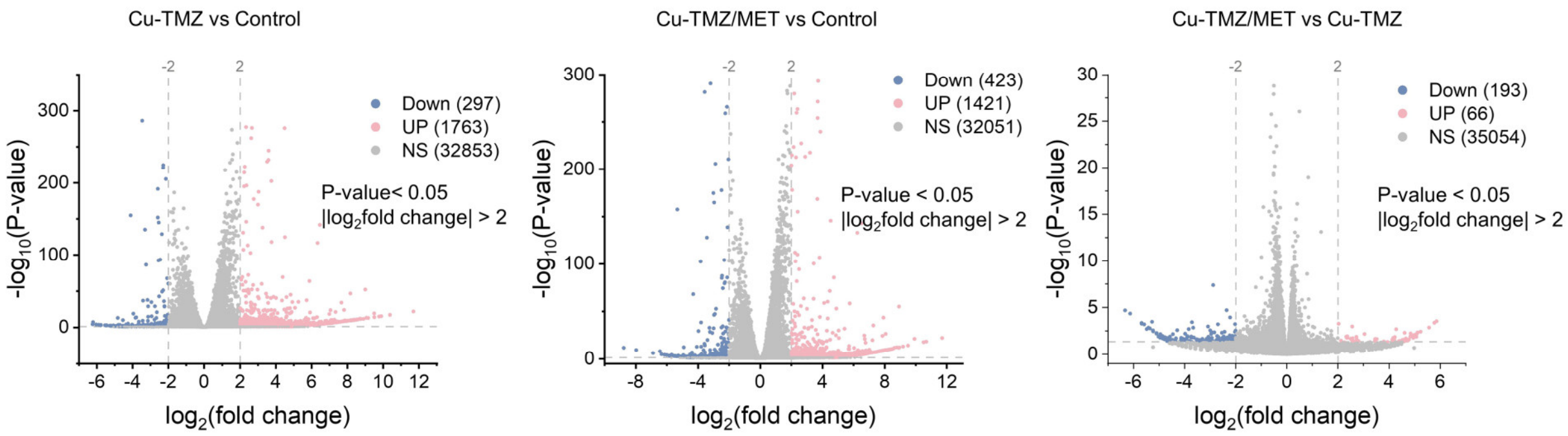

Figure S32. (a) The PPI of differentially expressed genes (Cu-TMZ/MET vs Control) in lipoic acid metabolism pathway in the STRING database. (b) Heat map analysis of differentially expressed genes in lipoic acid metabolism pathway after Cu-TMZ, or Cu-TMZ/MET prodrug treatment.

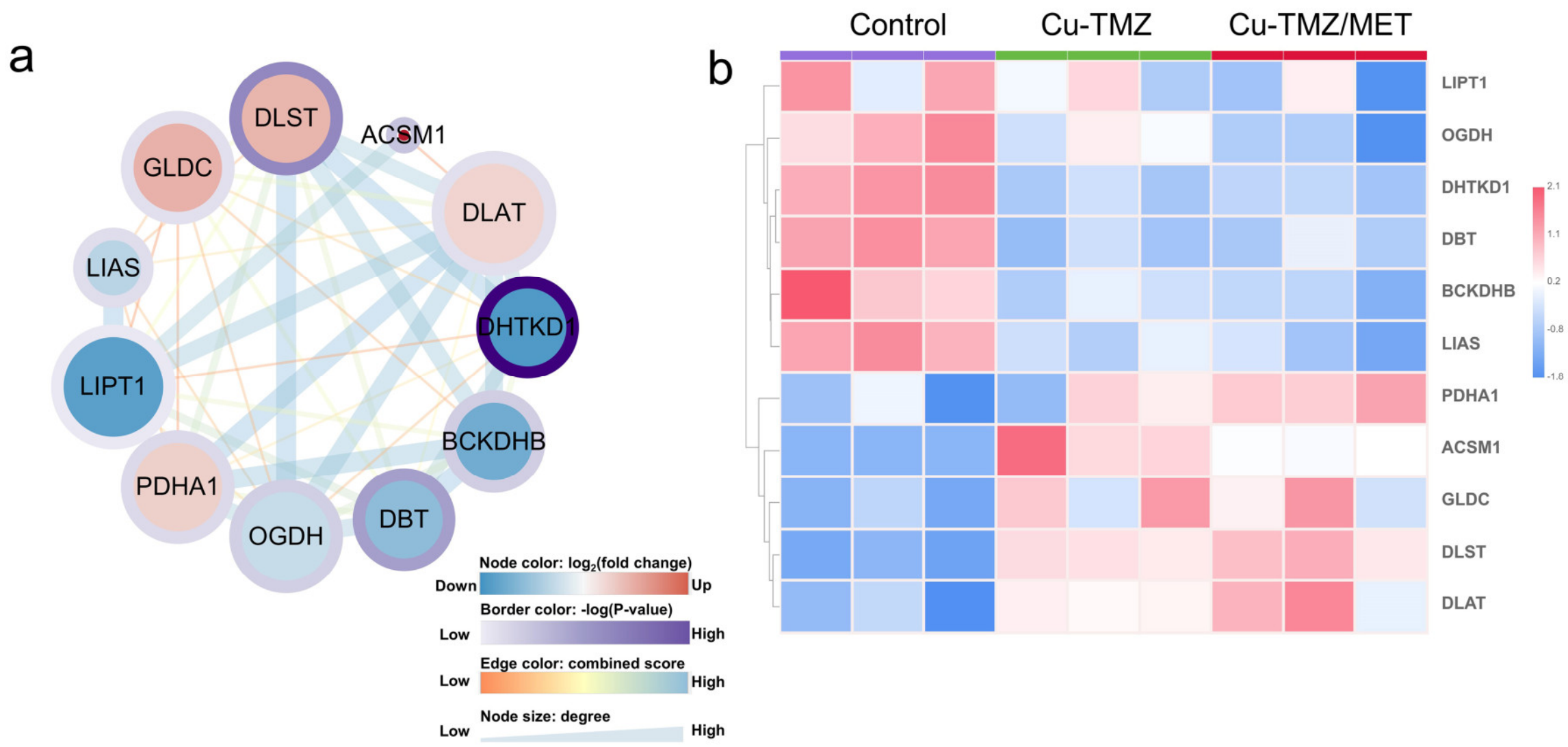

Figure S33. (a) GSEA analysis shows that the gene set of the TCA cycle pathway is enriched in Cu-TMZ/MET prodrug-treated cells. (b) The PPI of differentially expressed genes (Cu-TMZ/MET vs Control) in TCA cycle pathway in the STRING database.

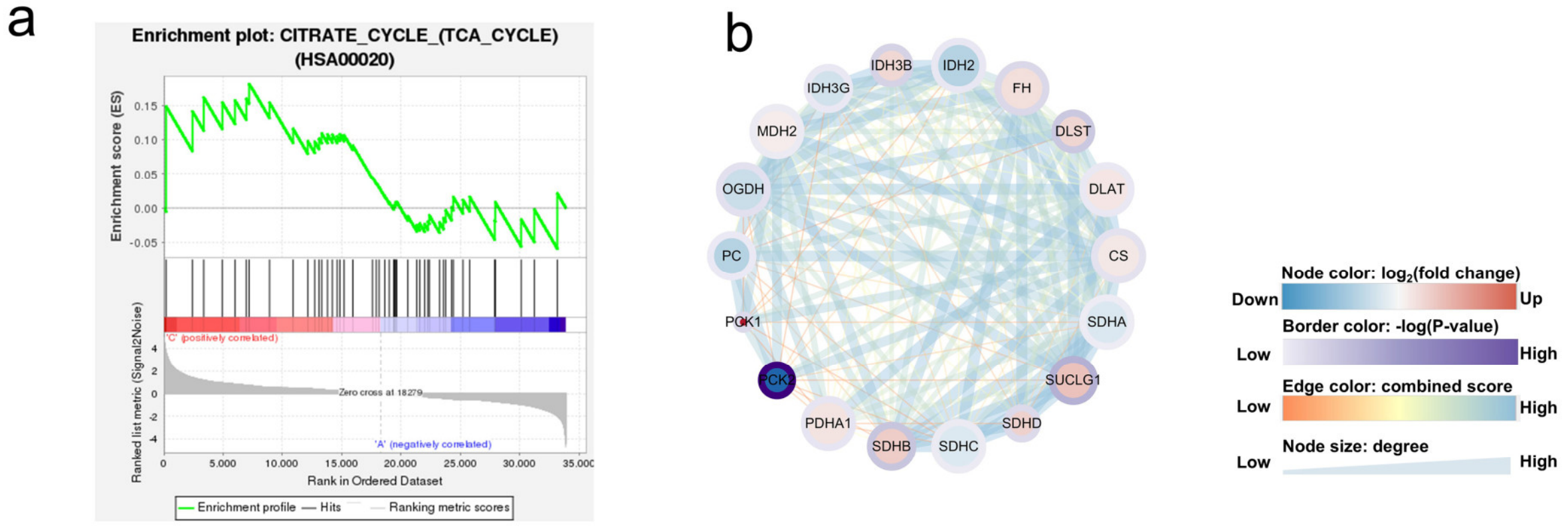

Figure S34. (a) GSEA analysis shows that the gene set of the mitochondria iron sulfur cluster biogenesis pathway is enriched in Cu-TMZ/MET prodrug-treated cells. (b) The PPI of differentially expressed genes (Cu-TMZ/MET vs Control) in mitochondria iron sulfur cluster biogenesis pathway in the STRING database. (c) Heat map analysis of differentially expressed genes in mitochondria iron sulfur cluster biogenesis pathway after Cu-TMZ, or Cu-TMZ/MET prodrug treatment.

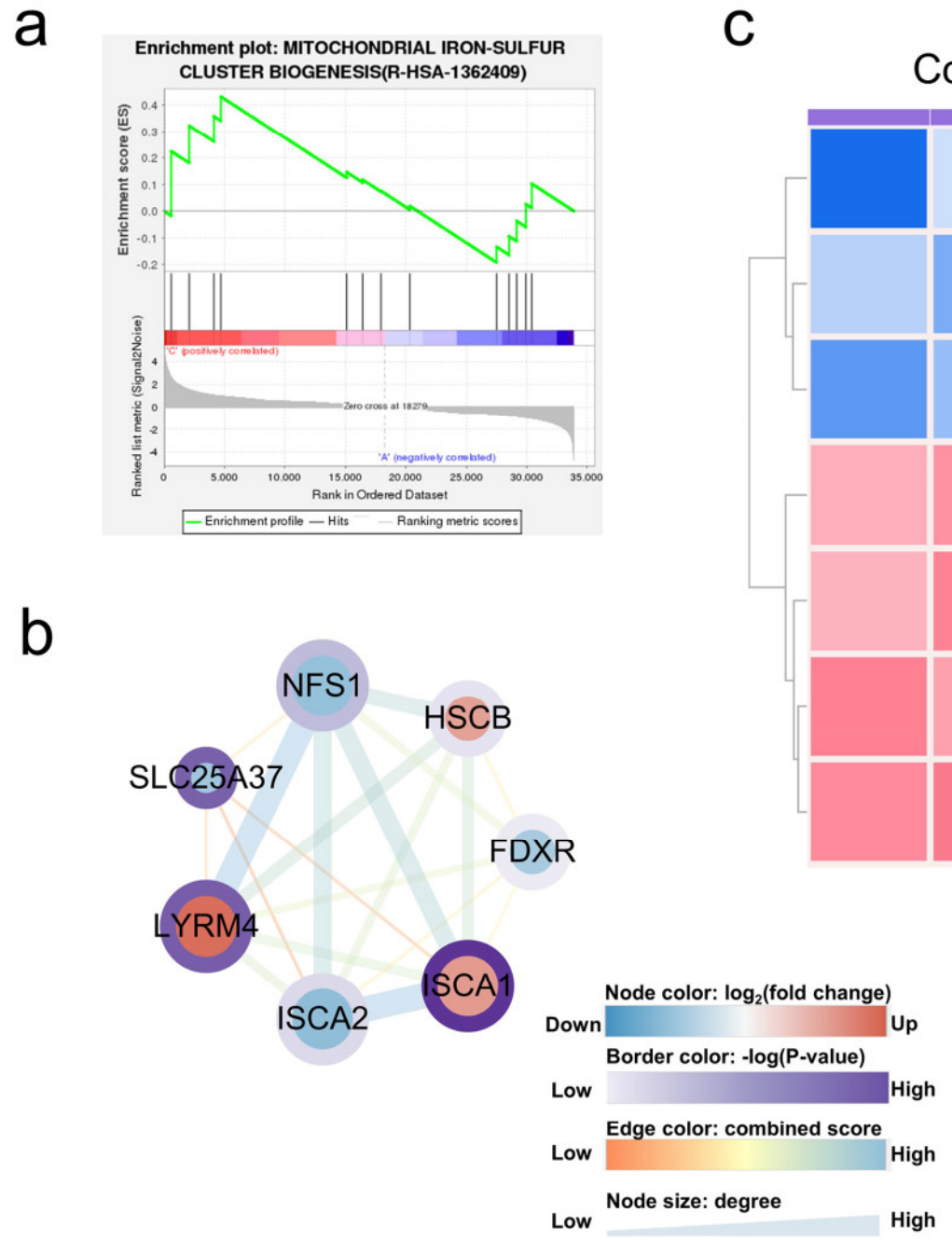

Figure S35. (a) GSEA analysis shows that the gene set of the regulation of mitochondrial membrane potential pathway is enriched in Cu-TMZ/MET prodrug-treated cells. (b) The PPI of differentially expressed genes (Cu-TMZ/MET vs Control) in regulation of mitochondrial membrane potential pathway in the STRING database. (c) Heat map analysis of differentially expressed genes in the regulation of mitochondrial membrane potential pathway after Cu-TMZ, or Cu-TMZ/MET prodrug treatment.

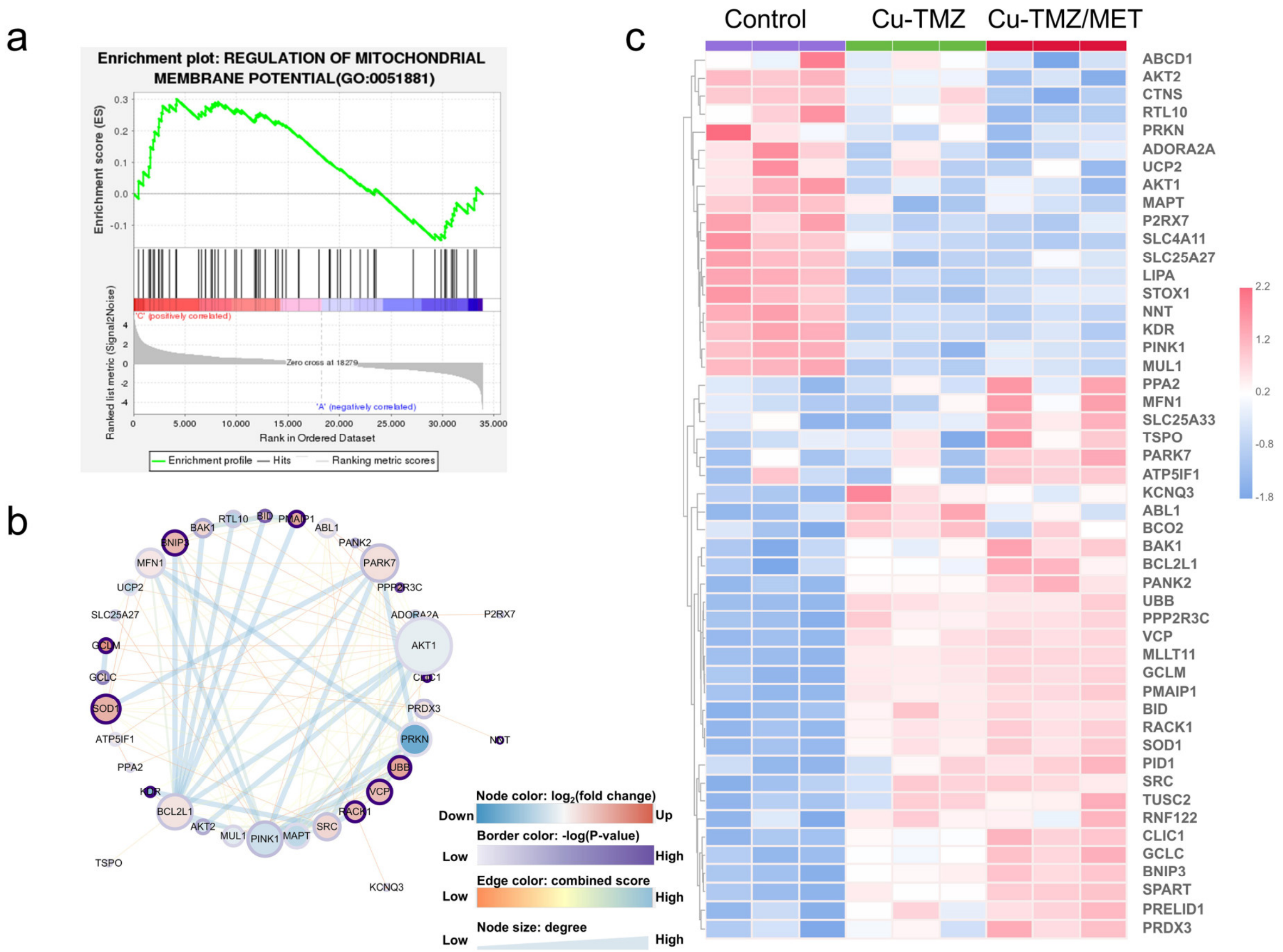

Figure S36. (a) GSEA analysis shows that the gene set of mitochondrial ATP synthesis coupled electron transport pathway is enriched in Cu-TMZ/MET prodrug-treated cells. (b) Heat map analysis of differentially expressed genes in mitochondrial ATP synthesis coupled electron transport pathway after Cu-TMZ, or Cu-TMZ/MET prodrug treatment.

a

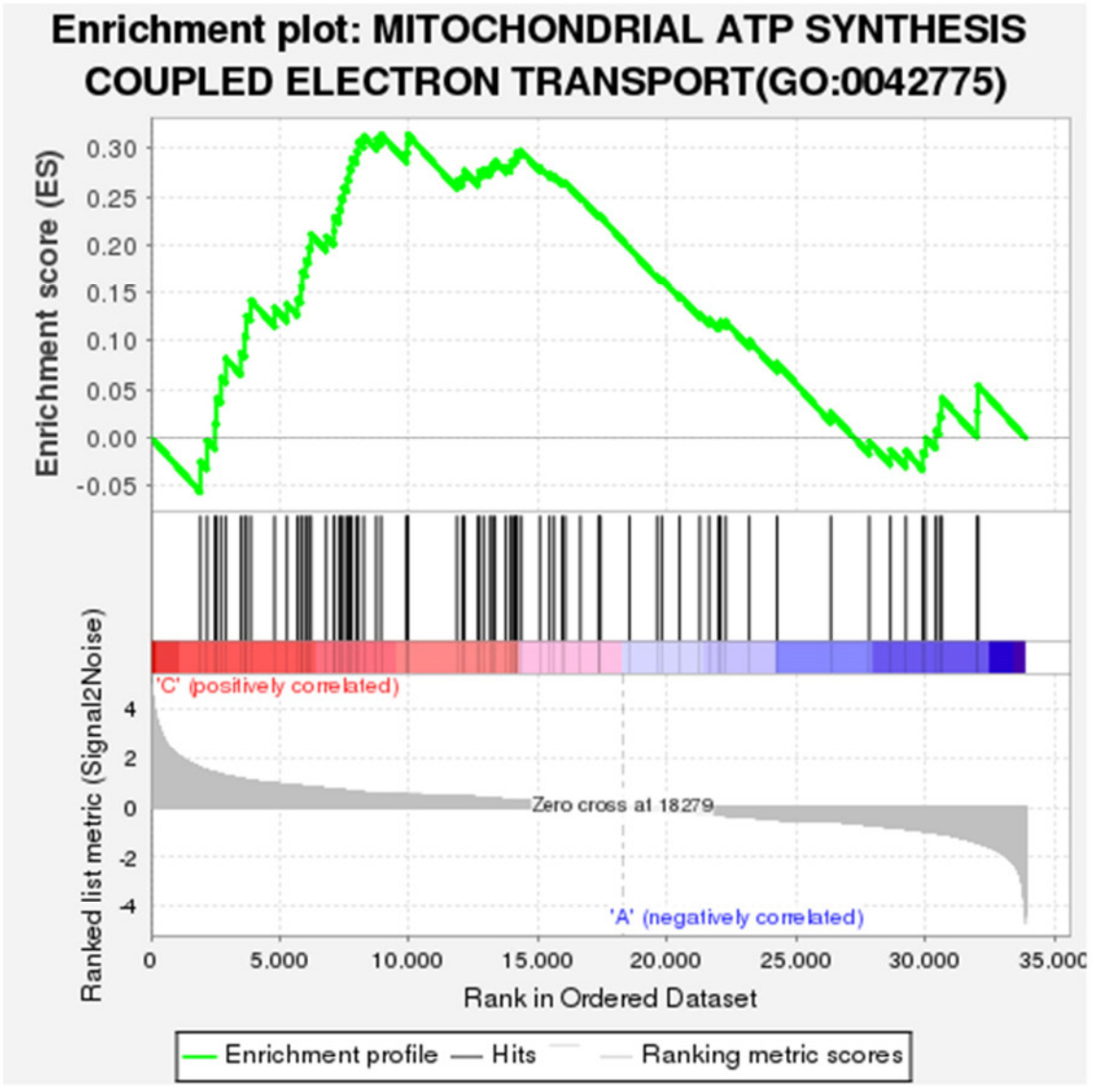

b

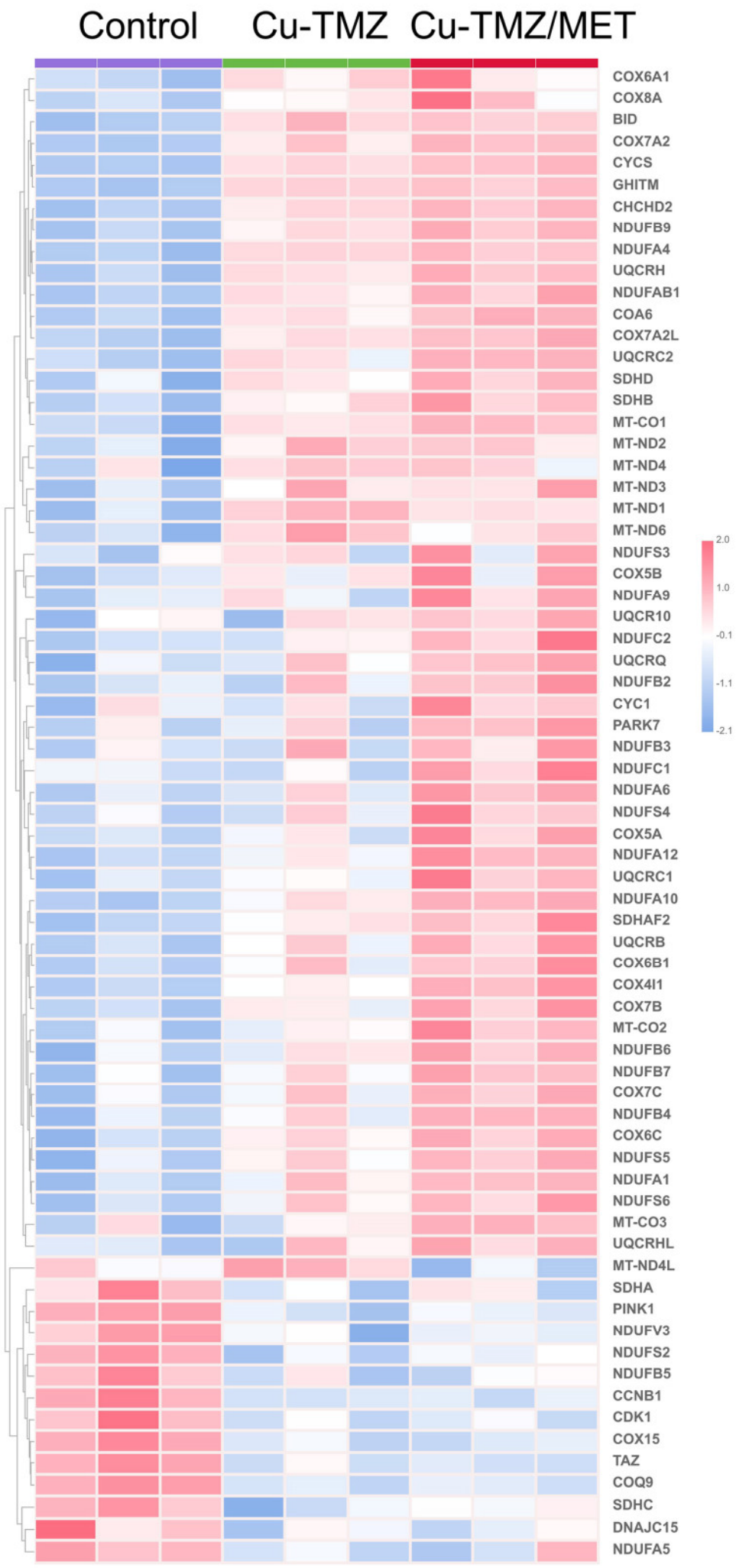

Figure S37. (a) GSEA analysis shows that the gene set of AMPK signaling pathway is enriched in Cu-TMZ/MET prodrug-treated cells. (b) The PPI of differentially expressed genes (Cu-TMZ/MET vs Control) in AMPK signaling pathway in the STRING database. (c) Heat map analysis of differentially expressed genes in AMPK signaling pathway after Cu-TMZ, or Cu-TMZ/MET prodrug treatment.

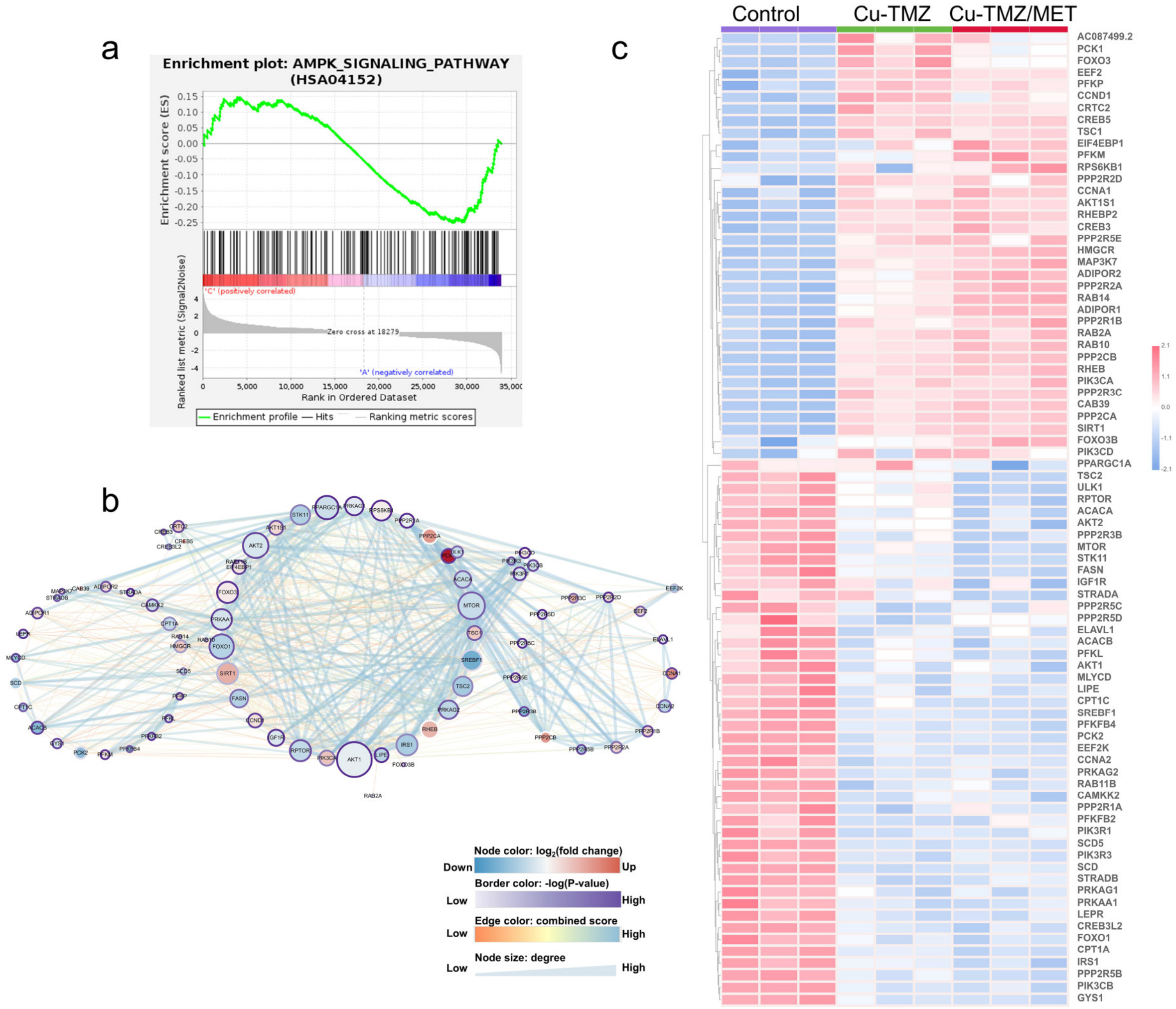

Figure S38. (a) GSEA analysis shows that the gene set of p53 signaling pathway is enriched in Cu-TMZ/MET prodrug-treated cells. (b) The PPI of differentially expressed genes (Cu-TMZ/MET vs Control) in p53 signaling pathway in the STRING database. (c) Heat map analysis of differentially expressed genes in p53 signaling pathway after Cu-TMZ, or Cu-TMZ/MET prodrug treatment.

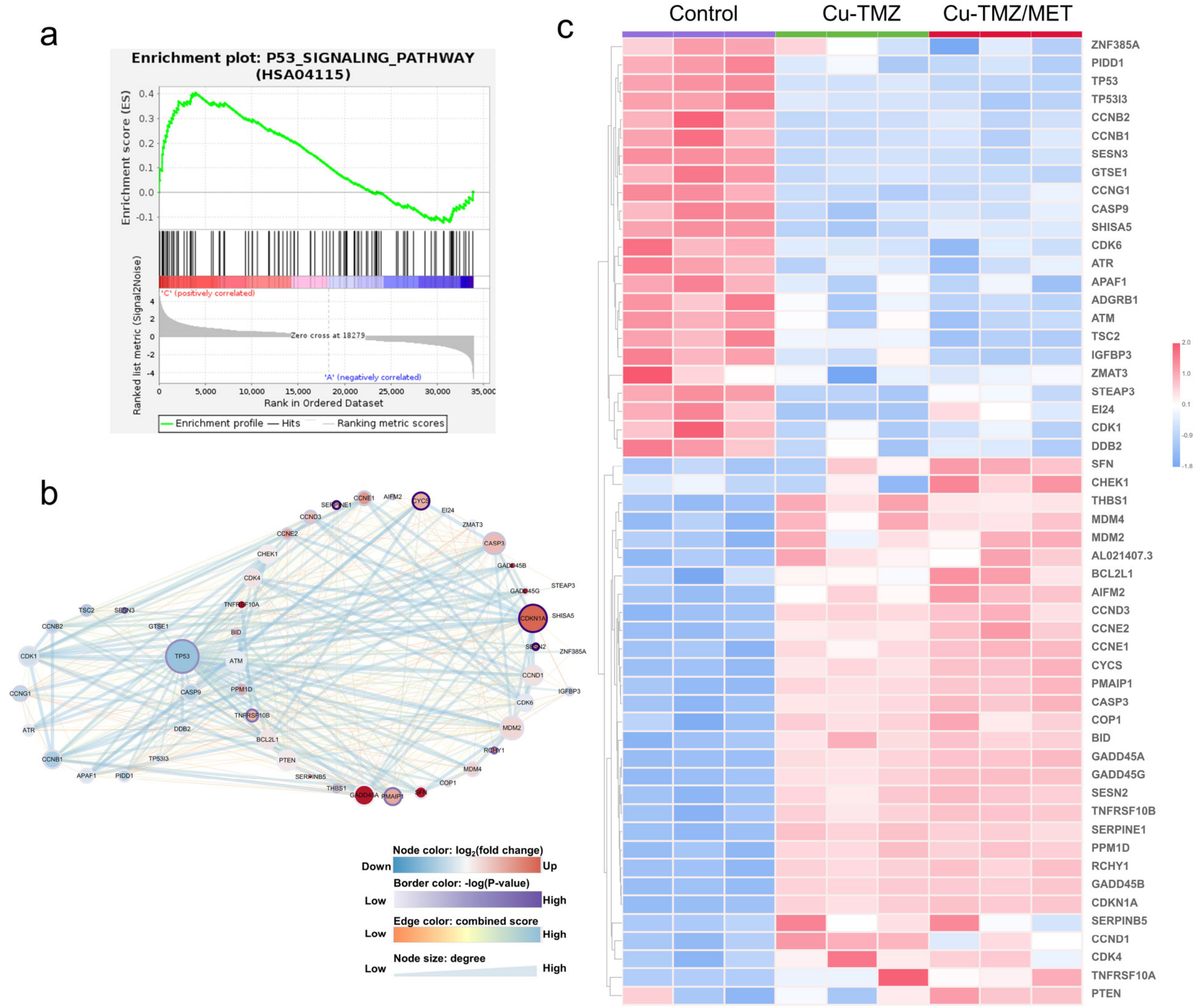

Figure S39. (a) GSEA analysis shows that the gene set of glycolytic process is enriched in Cu-TMZ/MET prodrug-treated cells. (b) The PPI of differentially expressed genes (Cu-TMZ/MET vs Control) in glycolytic process in the STRING database. (c) Heat map analysis of differentially expressed genes in glycolytic process after Cu-TMZ, or Cu-TMZ/MET prodrug treatment.

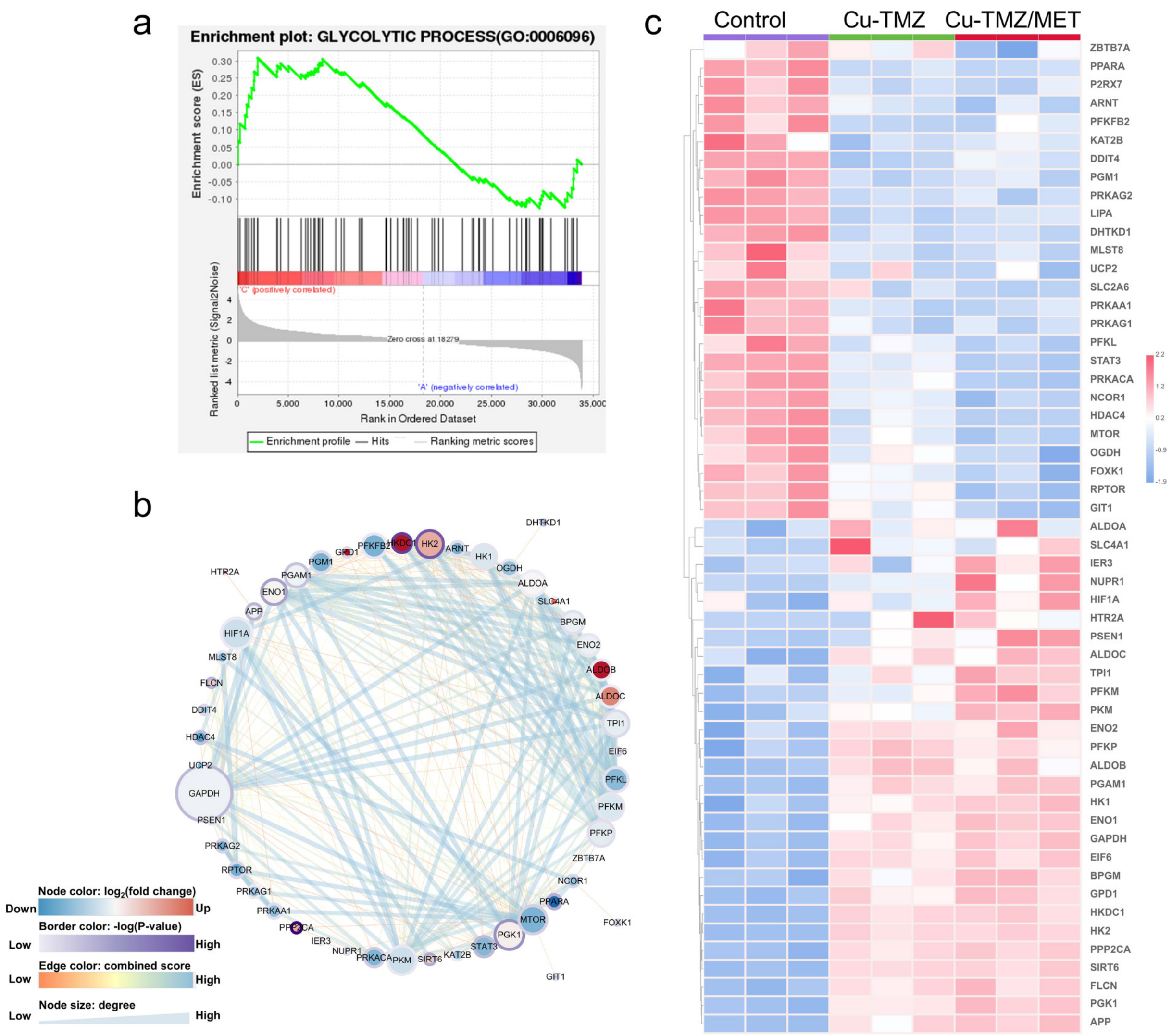

Figure S40. (a) The PPI of differentially expressed genes (Cu-TMZ/MET vs Control) in oxidative phosphorylation in the STRING database. (b) Heat map analysis of differentially expressed genes in oxidative phosphorylation after Cu-TMZ, or Cu-TMZ/MET prodrug treatment.

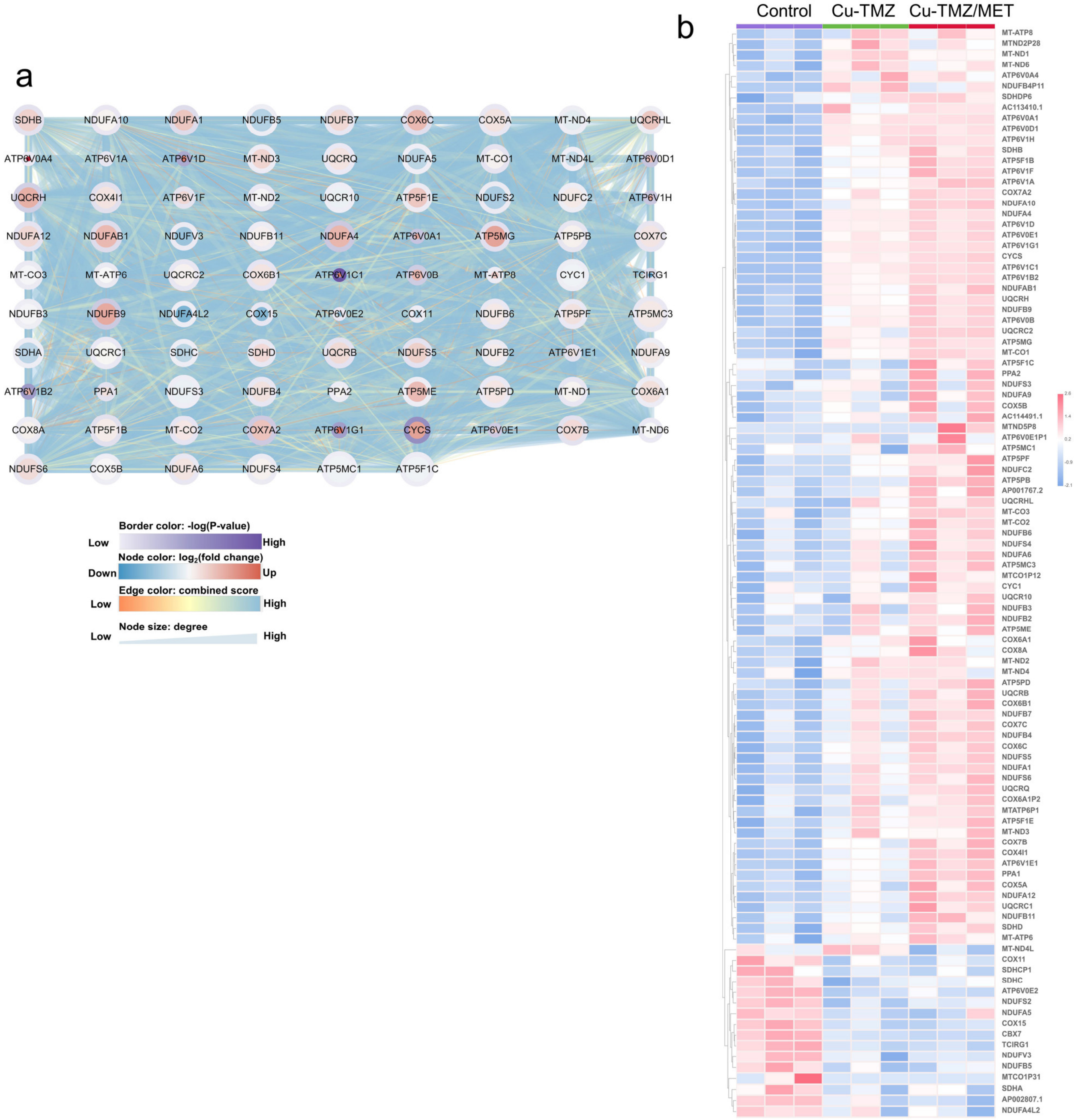

Figure S41. (a) GSEA analysis shows that the gene set of chemical carcinogenesis-ROS is enriched in Cu-TMZ/MET prodrug-treated cells. (b) The PPI of differentially expressed genes (Cu-TMZ/MET vs Control) in chemical carcinogenesis-ROS in the STRING database. (c) Heat map analysis of differentially expressed genes in chemical carcinogenesis-ROS after Cu-TMZ, or Cu-TMZ/MET prodrug treatment.

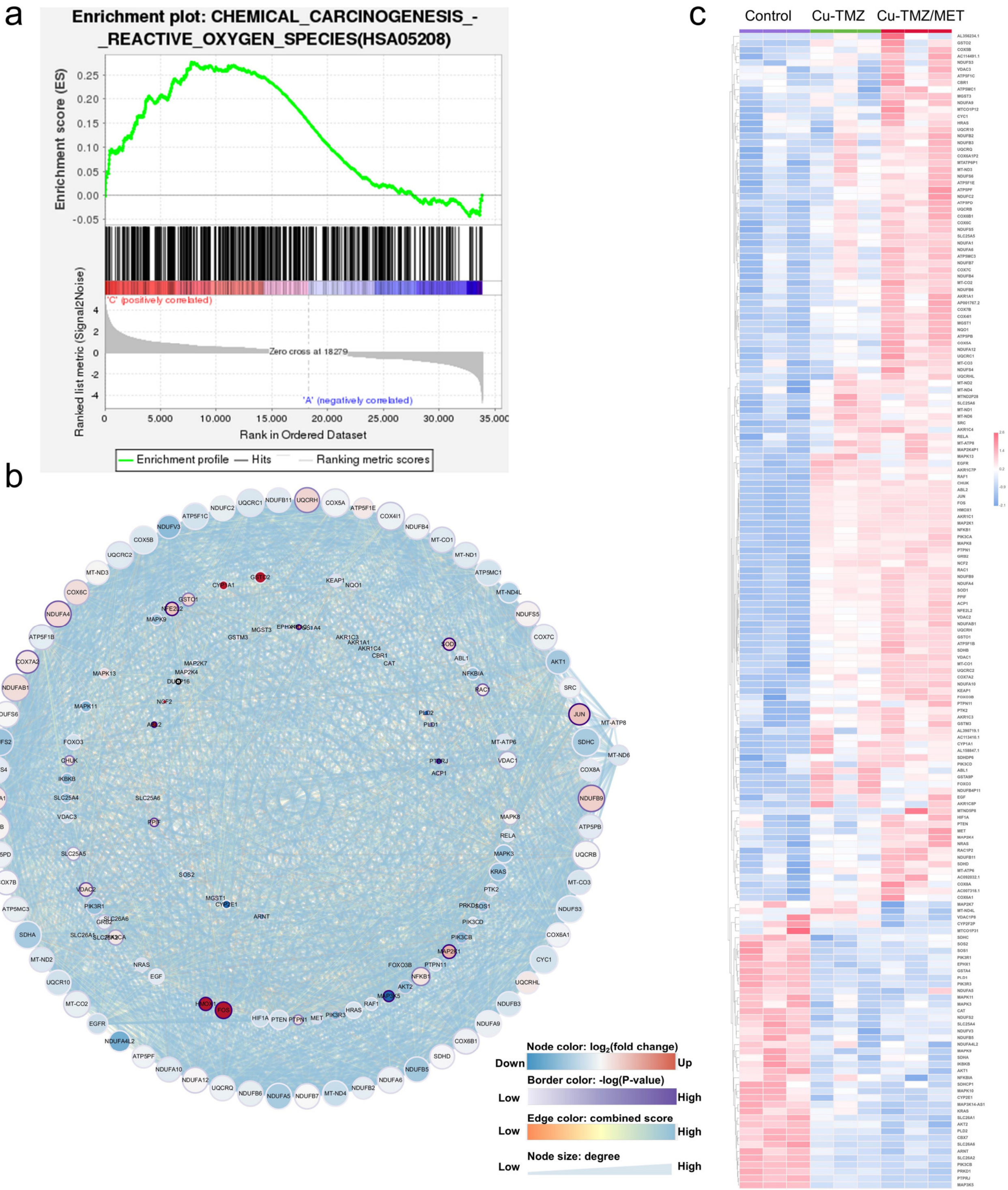

Figure S42. (a) GSEA analysis shows that the gene set of GSH metabolism process is enriched in Cu-TMZ/MET prodrug-treated cells. (b) The PPI of differentially expressed genes (Cu-TMZ/MET vs Control) in GSH metabolism process in the STRING database. (c) Heat map analysis of differentially expressed genes in GSH metabolism process after Cu-TMZ, or Cu-TMZ/MET prodrug treatment.

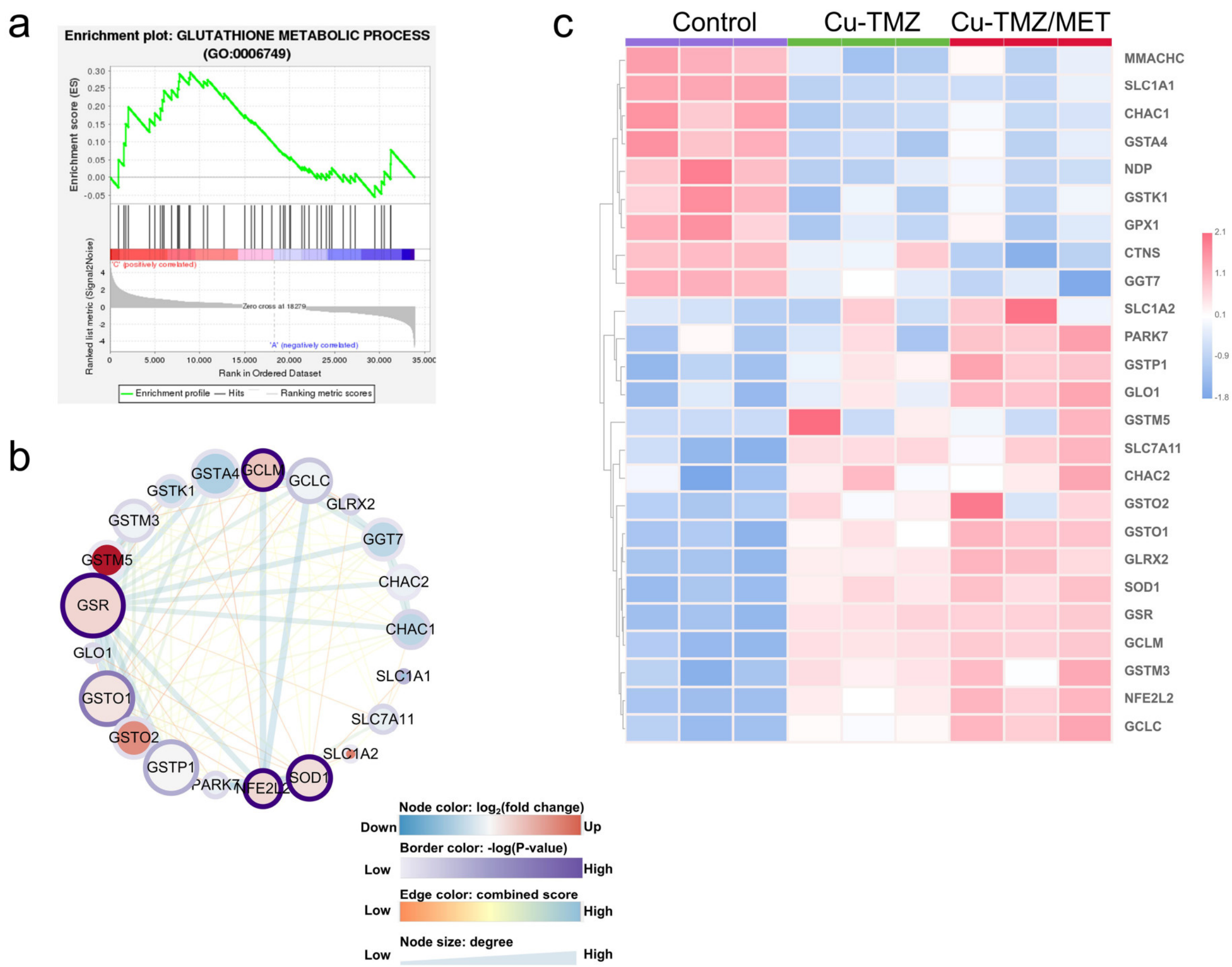

Figure S43. (a) GSEA analysis shows that the gene set of ABC transporters pathway is enriched in Cu-TMZ/MET prodrug-treated cells. (b) The PPI of differentially expressed genes (Cu-TMZ/MET vs Control) in ABC transporters pathway in the STRING database. (c) Heat map analysis of differentially expressed genes in ABC transporters pathway after Cu-TMZ, or Cu-TMZ/MET prodrug treatment.

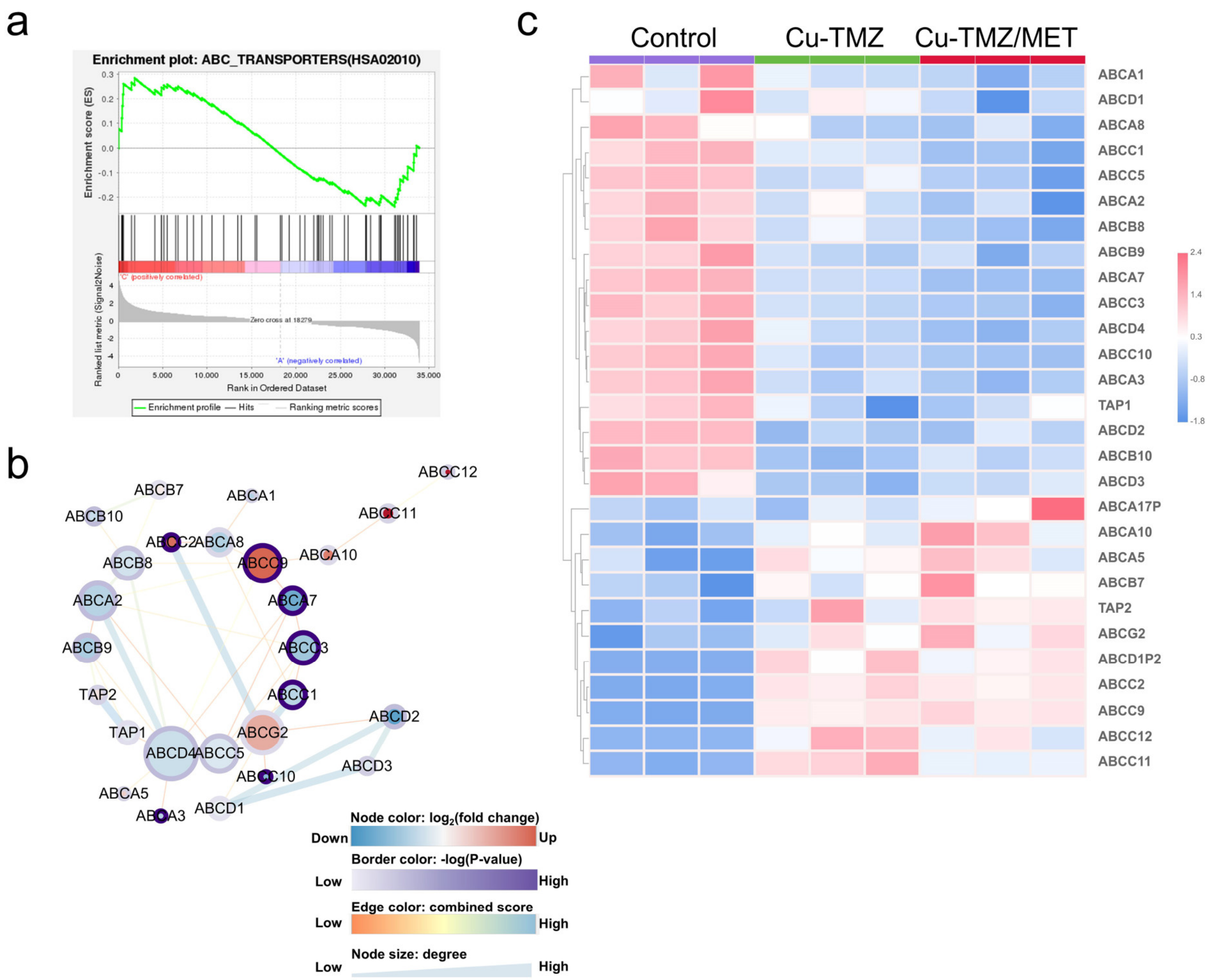

Figure S44. (a) GSEA analysis shows that the gene set of DNA damage bypass pathway is enriched in Cu-TMZ/MET prodrug-treated cells. (b) The PPI of differentially expressed genes (Cu-TMZ/MET vs Control) in DNA damage bypass pathway in the STRING database. (c) Heat map analysis of differentially expressed genes in DNA damage bypass pathway after Cu-TMZ, or Cu-TMZ/MET prodrug treatment.

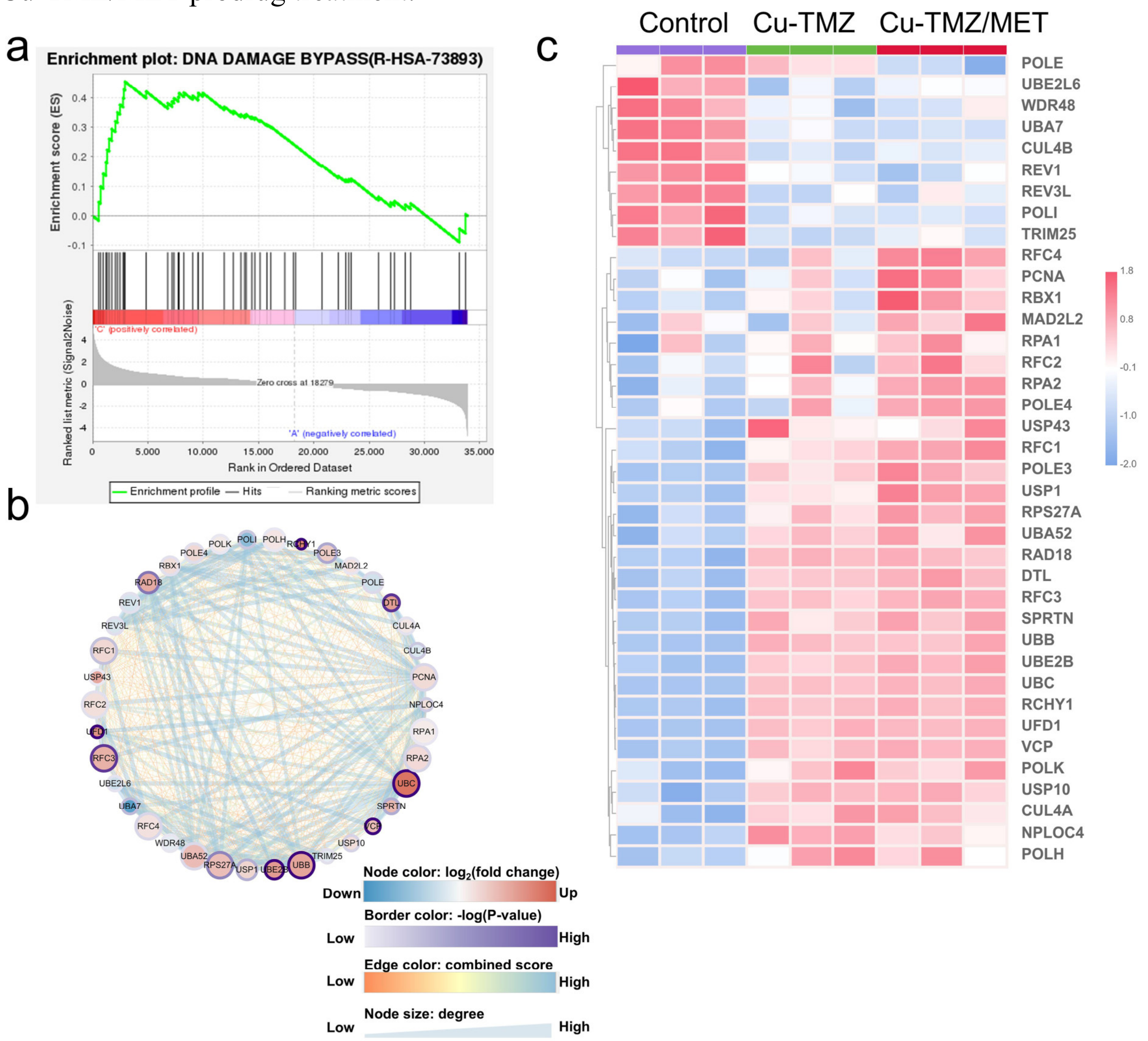

Figure S45. The PPI of differentially expressed genes (Cu-TMZ/MET vs Control) in DNA repair pathway in the STRING database.

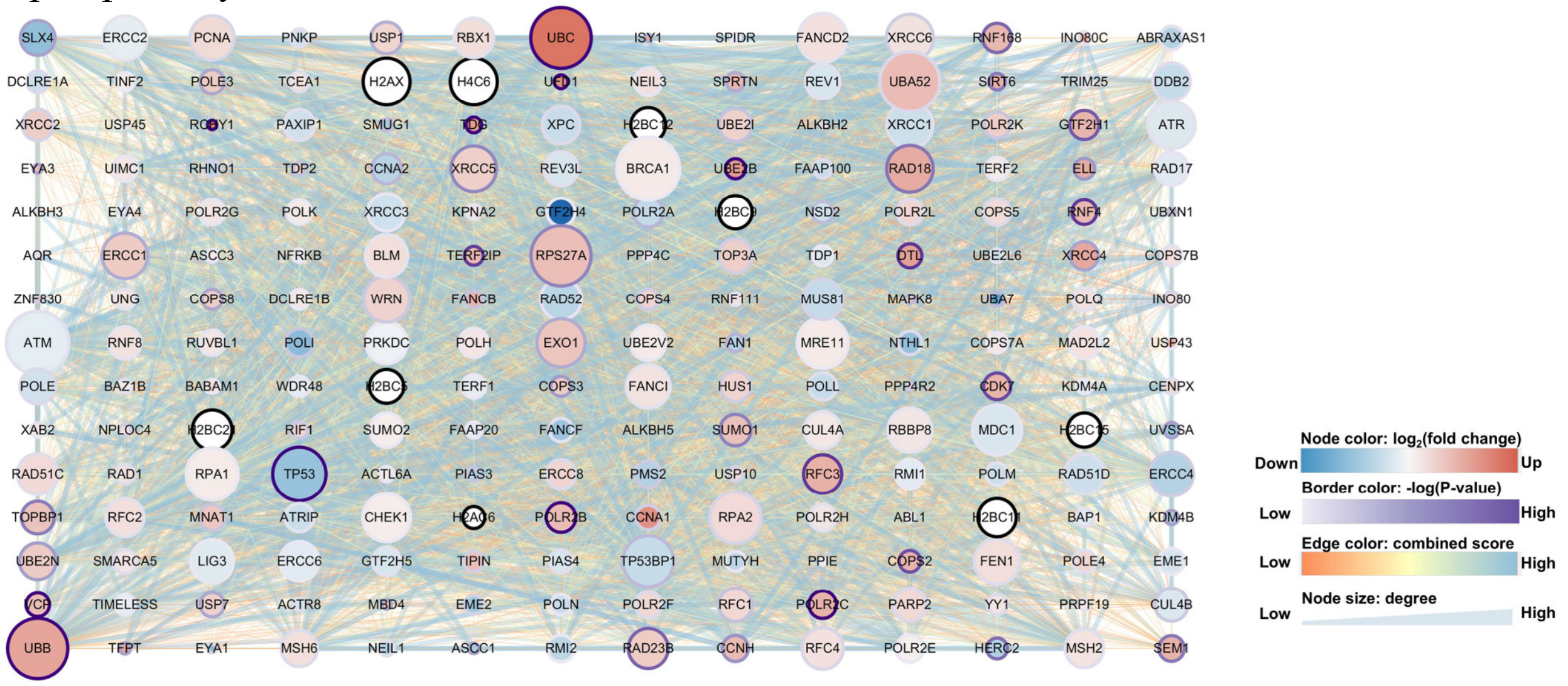

Figure S46. Heat map analysis of differentially expressed genes in DNA repair pathway after Cu-TMZ, or Cu-TMZ/MET prodrug treatment.

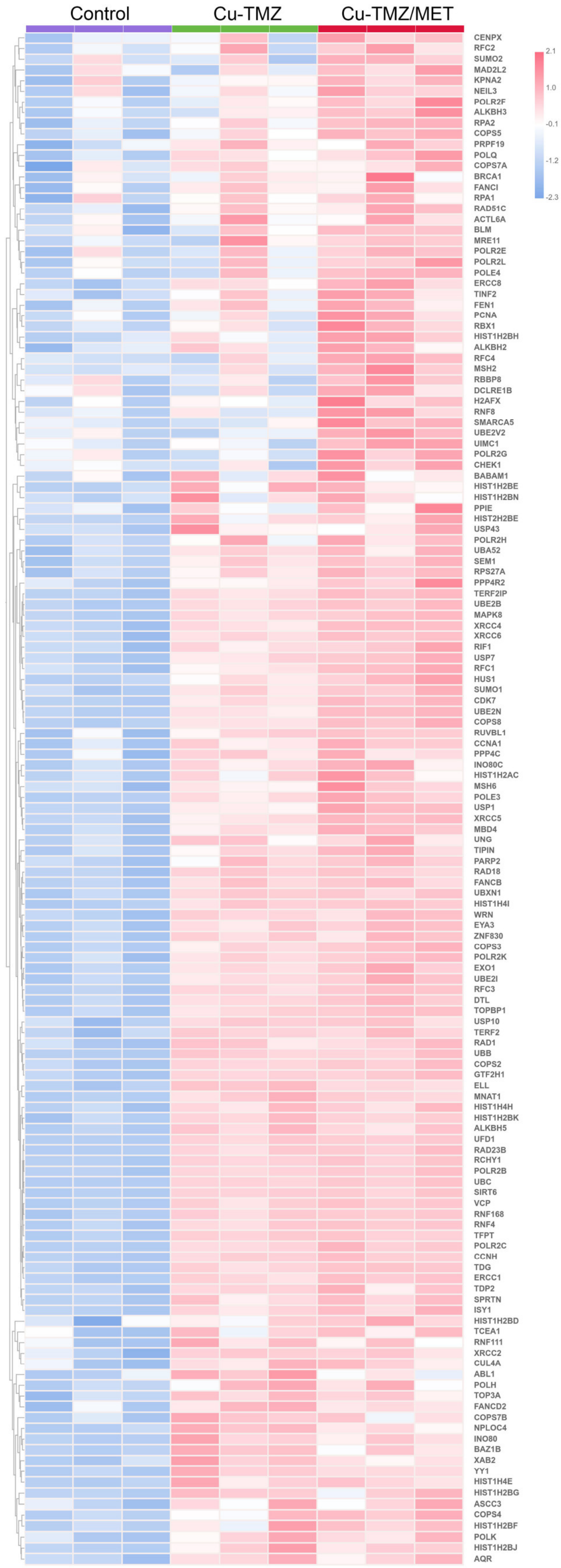

Figure S47. The PPI of differentially expressed genes (Cu-TMZ/MET vs Control) in DNA repair, p53 signaling pathway, GSH metabolic process, chemical carcinogenesis-ROS, glycolytic process, regulation of mitochondrial membrane potential, and ABC transporters in the STRING database.

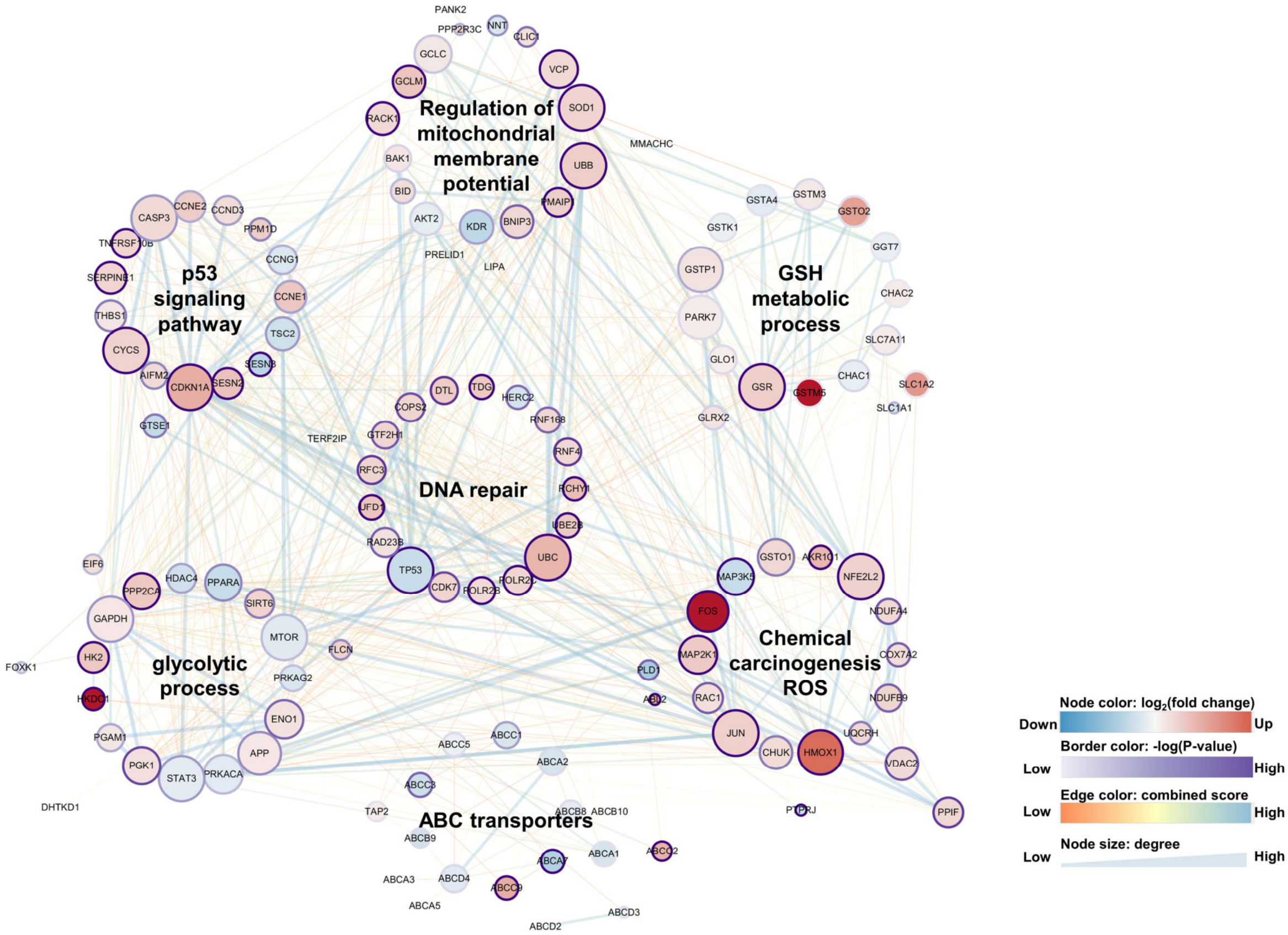

Figure S48. The pictures of individual tumor (a), individual tumor volume curve (b), and H&E-stained tumor slice (c) of U87 bearing mice in group I, and II. Groups: (I) Control, and (II) TMZ.

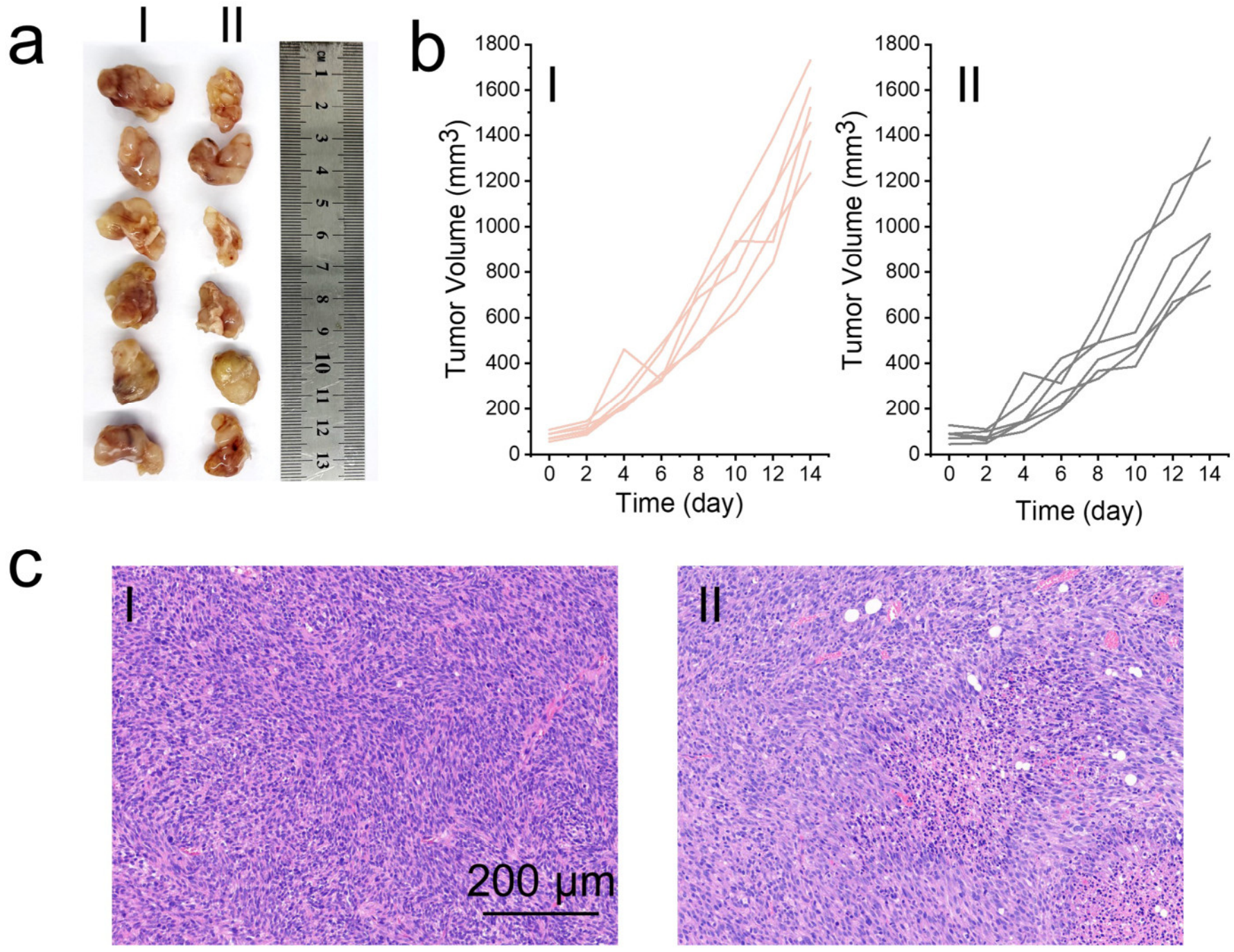

Figure S49. The pictures of individual tumor (a), and individual tumor volume curve (b) of U87/TR bearing mice in group I-group VI. Groups: (I) Control, (II) TMZ, (III) Cu^{2+}+TMZ+MET, (IV) Cu-TMZ, (V) Cu-TMZ/MET, and (VI) Cu-TMZ/MET + TTM.

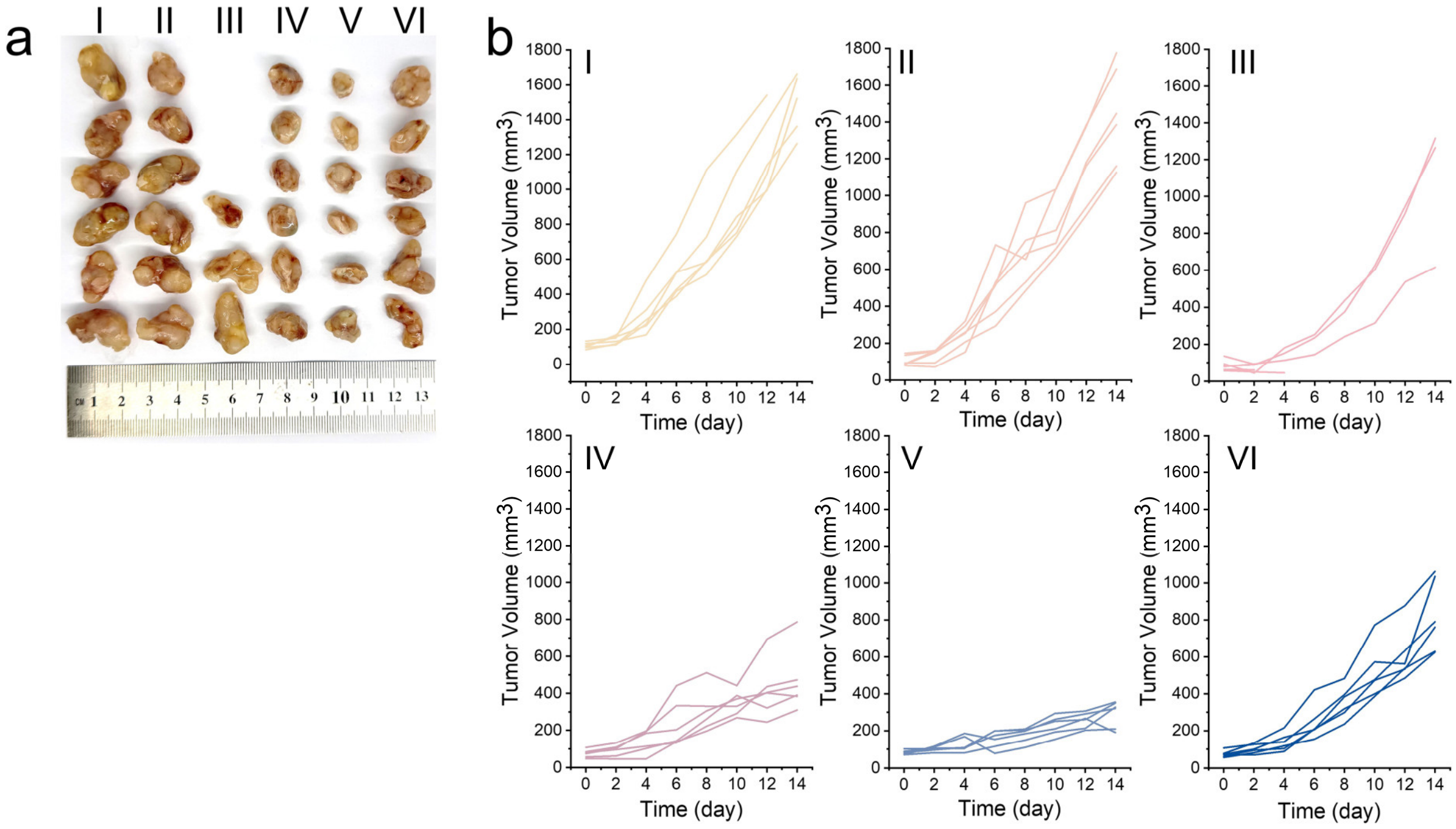

Figure S50. The expression level of DLAT in tumor tissue by WB analysis in group I-group VI. Groups: (I) Control, (II) TMZ, (III) Cu^{2+}+TMZ+MET, (IV) Cu-TMZ, (V) Cu-TMZ/MET, and (VI) Cu-TMZ/MET+TTM.

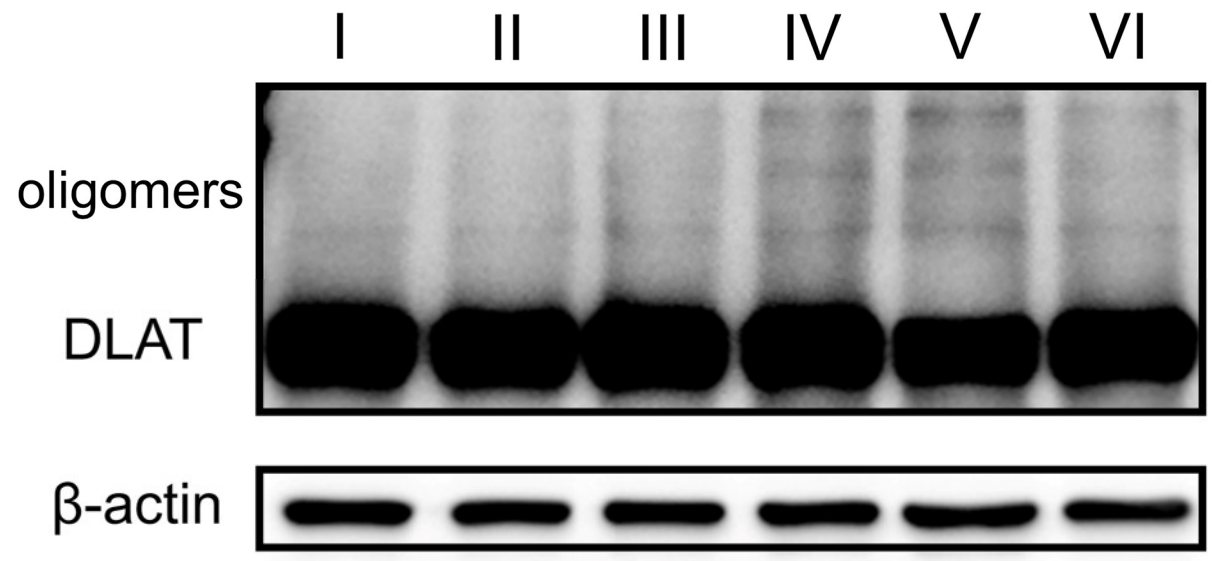

Figure S51. Fluorescence images (a) and fluorescence intensity (b) of *in vivo* orthotopic GBM bearing mice after intravenous injection of ^{IR780}Cu-TMZ/MET prodrugs from 0 to 24 h (n=3). (c) Fluorescence intensity of *ex vivo* brain and major organs dissected from mice after intravenous injection for 24 h (n=3). Inset in Figure S51c: fluorescence images of *ex vivo* brain and major organs. Data are presented as mean ± SD.

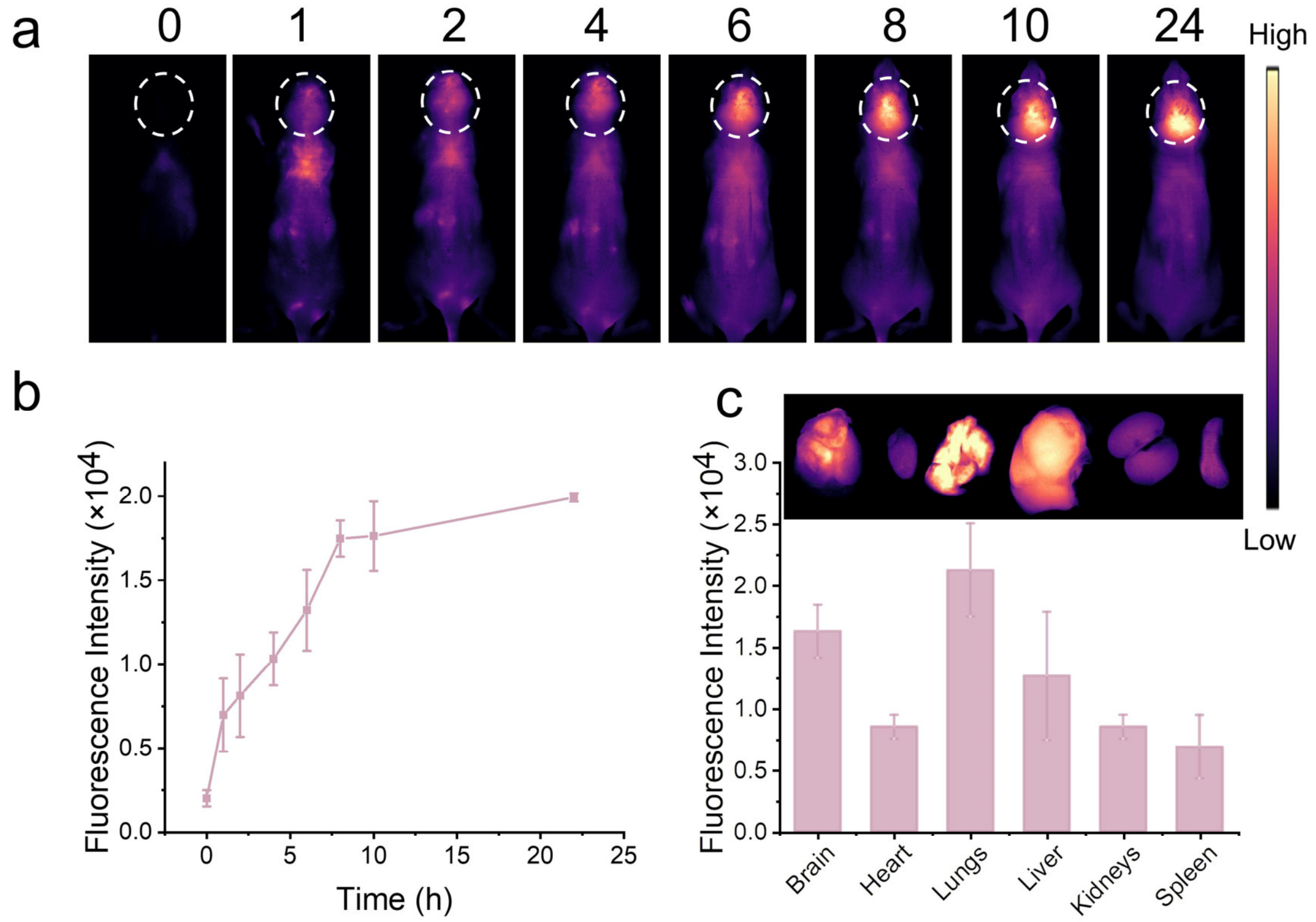

Figure S52. (a) Fluorescence images of blood fetched from mice after intravenous injection of ^{IR780}Cu-TMZ/MET prodrugs from 0 to 5 h. (b) The calculated half-life according to the fitted curve of decayed fluorescence intensity in Figure S52a.

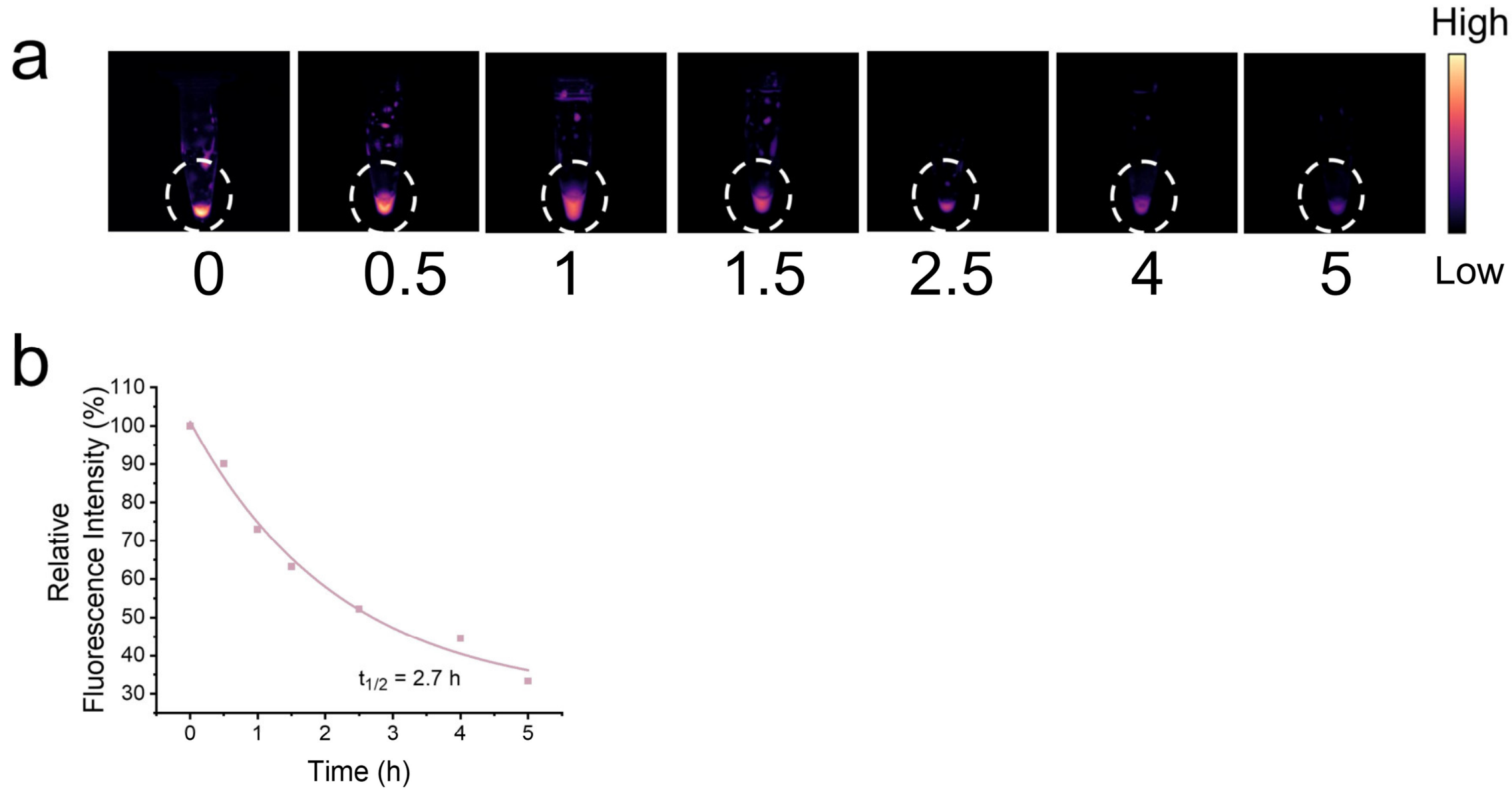

Figure S53. Individual bioluminescence intensity curve of GBM-Luc bearing mice in group I-group VI. Groups: (I) Control, (II) TMZ, (III) Cu^{2+}+TMZ+MET, (IV) Cu-TMZ, (V) Cu-TMZ/MET, and (VI) Cu-TMZ/MET + TTM.

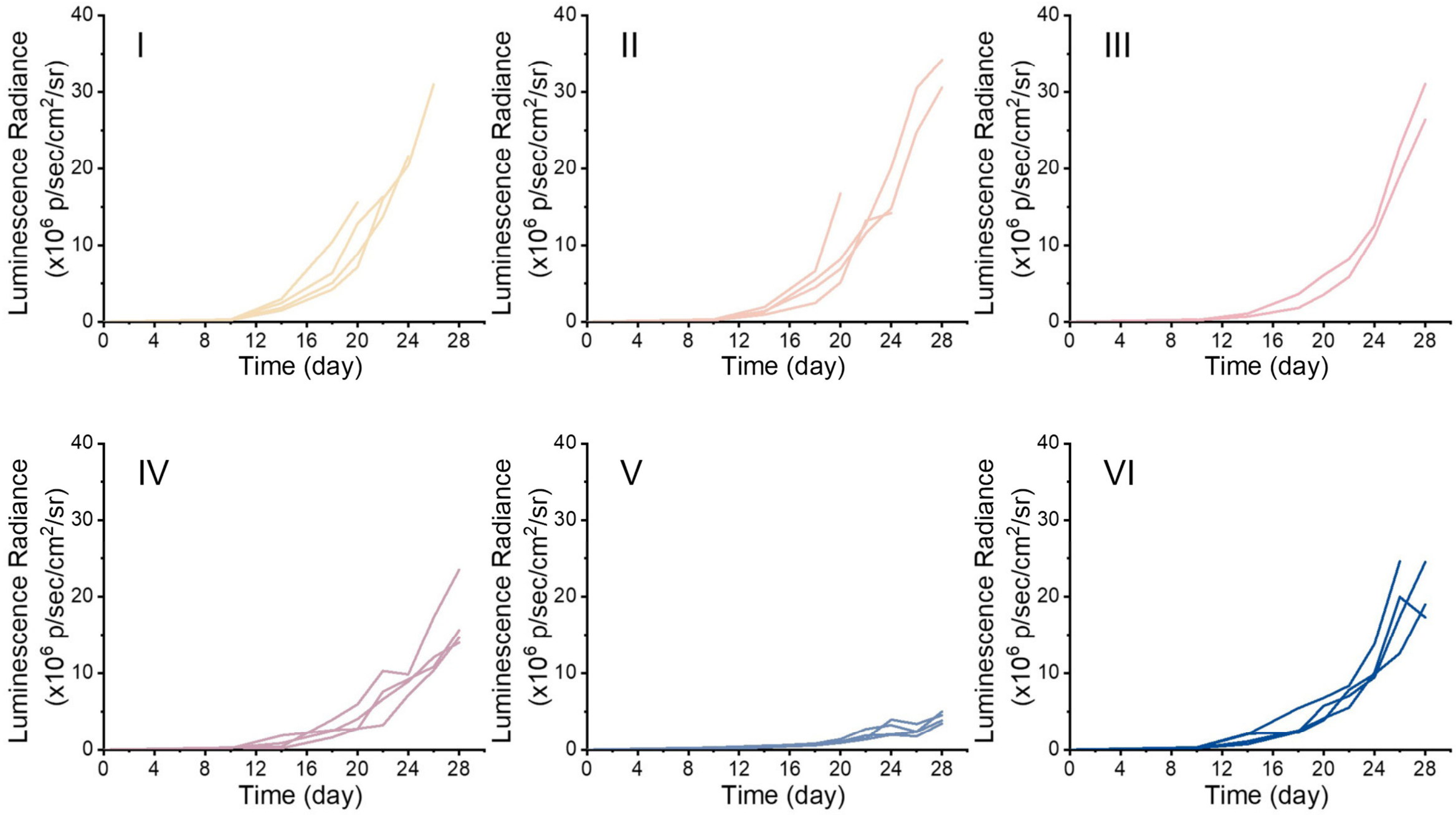

Figure S54. The hemolysis assay of Cu-TMZ (a) and Cu-TMZ/MET (b) prodrugs (n=3). Data are presented as mean ± SD.

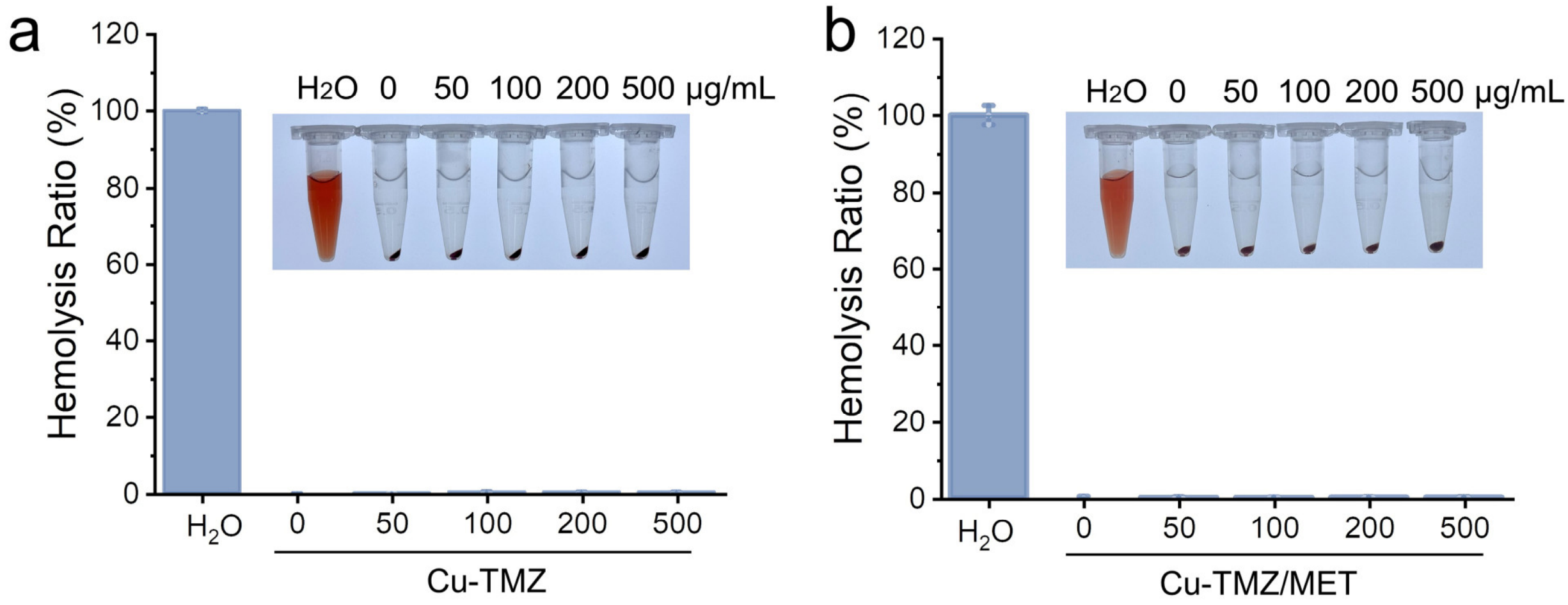

Figure S55. The H&E-stained slices of major organs of the mice in group I-group VI. Groups: (I) Control, (II) TMZ, (III) Cu^{2+}+TMZ+MET, (IV) Cu-TMZ, (V) Cu-TMZ/MET, and (VI) Cu-TMZ/MET+TTM.

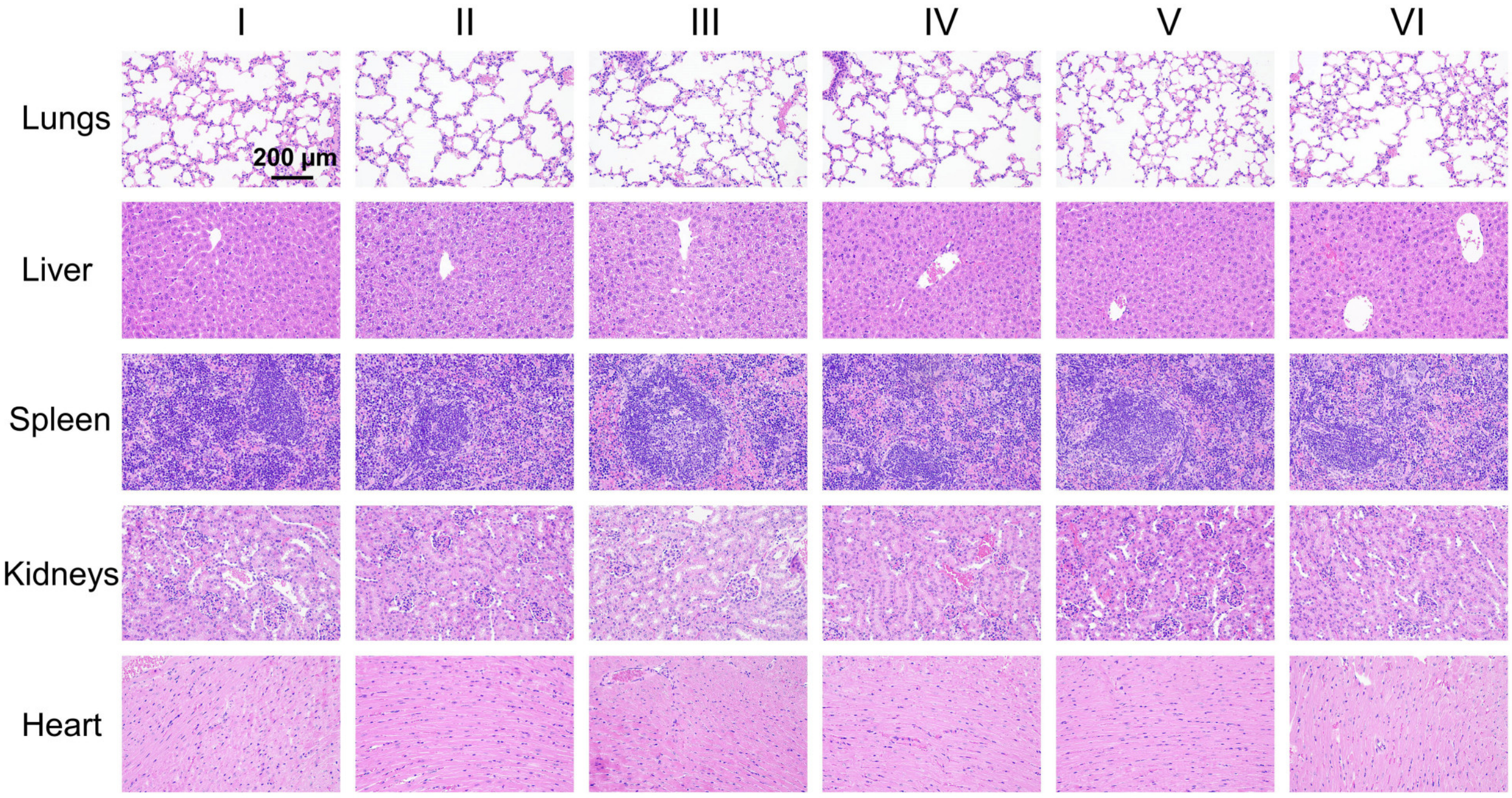

Figure S56. The H&E-stained slices of brain tissue in mice after intravenous administration of Cu-TMZ and Cu-TMZ/MET prodrugs.

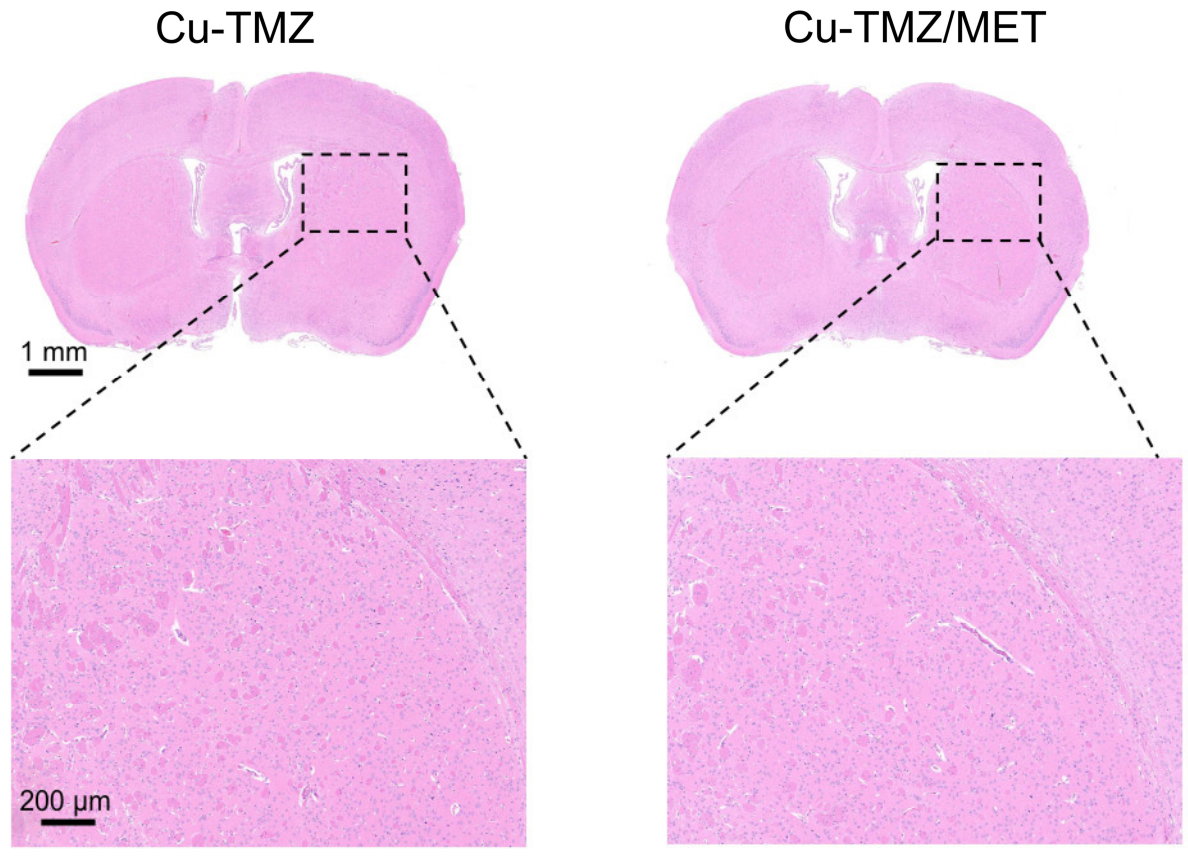

Figure S57. The indexes of liver function (a), including ALT, AST, TBIL, DBIL, ALB, ALP, γ-GT, and TBA, and kidney function (b), including UREA, CREA, and UA, of the mice in group I-group VI (n=3). Groups: (I) Control, (II) TMZ, (III) Cu^{2+}+TMZ+MET, (IV) Cu-TMZ, (V) Cu-TMZ/MET, and (VI) Cu-TMZ/MET+TTM. Data are presented as mean ± SD.

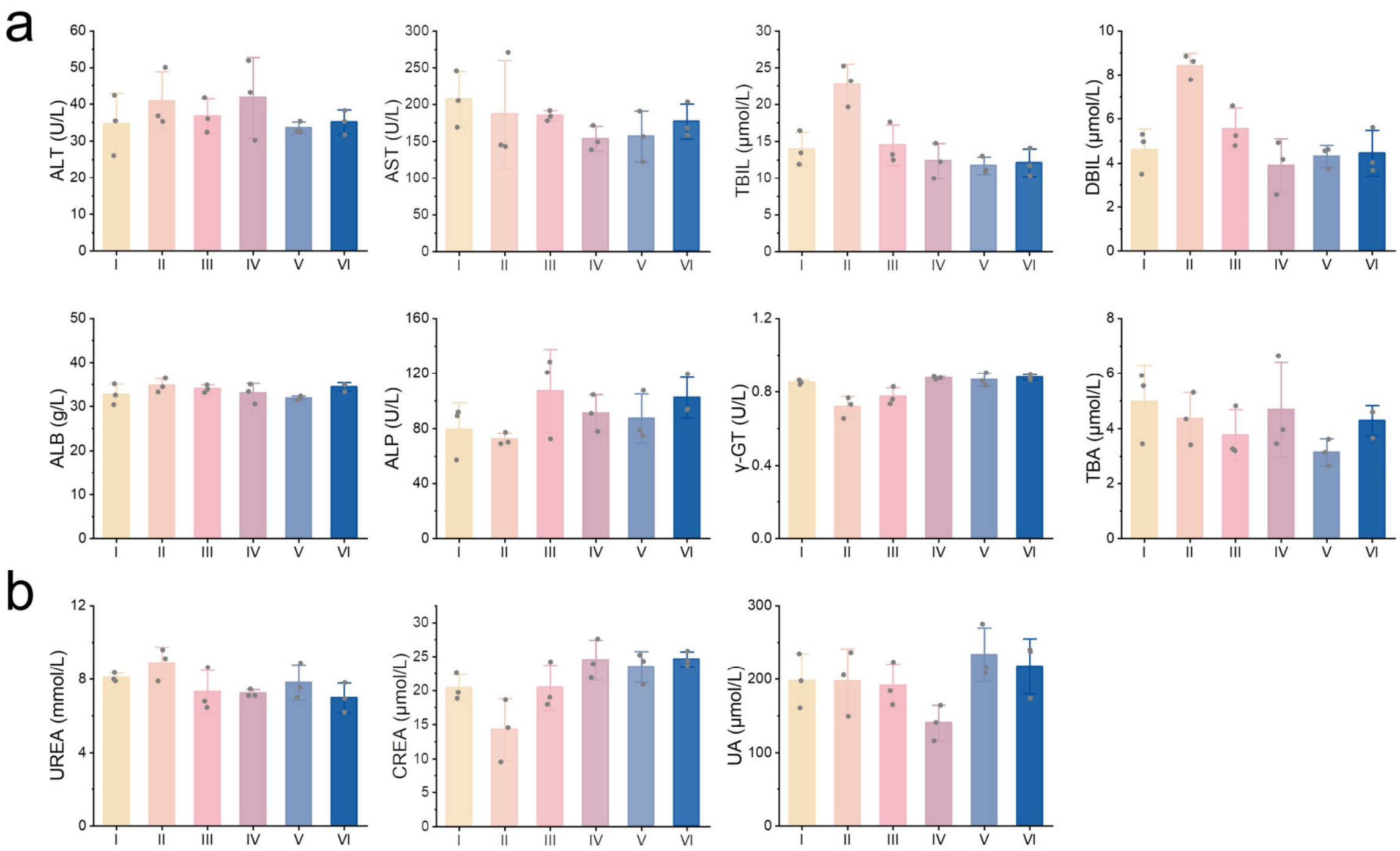

Supplement: Supplementary file 1 [file ADVS-13-e76677-s001.pdf]
